# Unveiling the chemical kinetics of aminomethanol (NH_2_CH_2_OH): insights into $\dot{O}$ H and O_2_ photo-oxidation reactions and formamide dominance

**DOI:** 10.3389/fchem.2024.1407355

**Published:** 2024-05-30

**Authors:** Naga Venkateswara Rao Nulakani, Mohamad Akbar Ali

**Affiliations:** ^1^ Department of Chemistry, Khalifa University of Science and Technology, Abu Dhabi, United Arab Emirates; ^2^ Center for the Catalyst and Separations, Khalifa University of Science and Technology, Abu Dhabi, United Arab Emirates

**Keywords:** aminomethanol, photooxidalion, DFT, transition state theory, formamide, rate constant

## Abstract

Aminomethanol is released into the atmosphere through various sources, including biomass burning. In this study, we have expounded the chemical kinetics of aminomethanol in the reaction pathways initiated by the hydroxyl radical ( $\dot{O}$ H) with the aid of *ab initio*//density functional theory (DFT) *i.e.*, coupled-cluster theory (CCSD(T))//hybrid-DFT (M06-2X/6-311++G (3df, 3pd). We have explored various possible directions of the $\dot{O}$ H radical on aminomethanol, as well as the formation of distinct pre-reactive complexes. Our computational findings reveal that the H transfer necessitates activation energies ranging from 4.1 to 6.5 kcal/mol from the –CH_2_ group, 3.5–6.5 kcal/mol from the –NH_2_ group and 7–9.3 kcal/mol from the –OH group of three rotational conformers. The H transfer from –CH_2_, –NH_2_ and –OH exhibits an estimated total rate constant (*k*_OH_) of approximately 1.97 × 10^−11^ cm^3^ molecule^−1^ s^−1^ at 300 K. The branching fraction analysis indicates a pronounced dominance of C-centered NH_2_ $\dot{C}$ HOH radicals with a favorability of 77%, surpassing the N-centered $\dot{N}$ HCH_2_OH (20%) and O-centered NH_2_CH_2_ $\dot{O}$ (3%) radicals. Moreover, our investigation delves into the oxidation of the prominently favored carbon-centered NH_2_ $\dot{C}$ HOH radical through its interaction with atmospheric oxygen molecules. Intriguingly, our findings reveal that formamide (NH_2_CHO) emerges as the predominant product in the NH_2_ $\dot{C}$ HOH + ^3^O_2_ reaction, eclipsing alternative outcomes such as amino formic acid (NH_2_COOH) and formimidic acid (HN = C(H)-OH). At atmospheric conditions pertinent to the troposphere, the branching fraction value for the formation of formamide is about 99%, coupled with a rate constant of 5.5 × 10^−12^ cm^3^ molecule^−1^ s^−1^. Finally, we have scrutinized the detrimental impact of formamide on the atmosphere. Interaction of formamide with atmospheric hydroxyl radicals could give rise to the production of potentially perilous compounds such as HNCO. Further, unreacted $\dot{N}$ HCH_2_OH radicals may initiate the formation of carcinogenic nitrosamines when reacting with trace N-oxides (namely, NO and NO_2_). This, in turn, escalates the environmental risk factors.

## Graphical Abstract

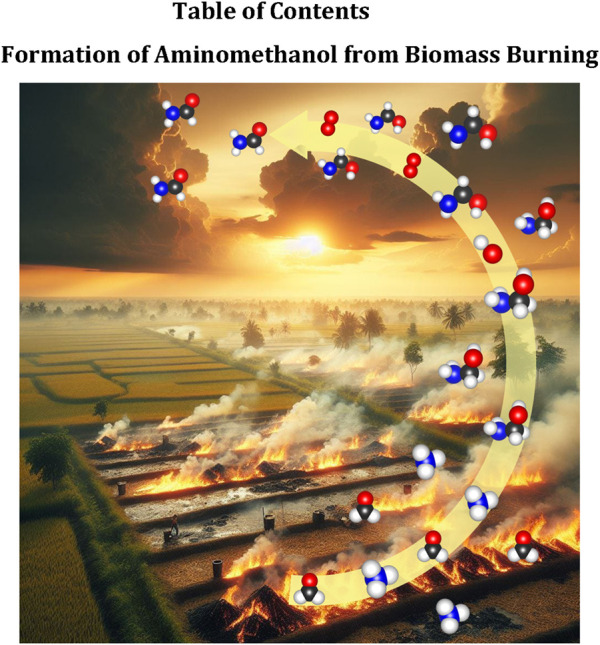

## 1 Introduction

Amines, especially those bearing the –NH_2_ functional group (RNH_2_), find extensive applications in the chemical industry, including uses in solvents, catalysis, surfactants, pharmaceuticals, adhesives, dyes and pigments, etc. (Ge et al., 2011a). For instance, carbon capture and storage (CCS) technology utilizes amine solvents to separate the CO_2_ emission from the acid gas treatment of natural gas and fossil fuel power plants (Dai et al., 2012; Yamada, 2021). The rapid development of CCS technology consequently leads to a significant increase in alkanolamines emissions into the atmosphere due to their high vapor pressure (Kapteina et al., 2005). Additionally, the amines are also released into the atmosphere by various other sources such as thorough industrial emission, wastewater treatment, animal husbandry and automotive activities, emissions from ocean organisms, biomass combustion and the degradation of proteins, etc. (Ge et al., 2011b). These amines undergo degradation and participate in conversion reactions both in gas and aqueous phase (clouds, raindrops, fog) in the atmosphere. As a result, they have detrimental effects on air quality, leading to issues like acid rain, urban smog and tropospheric ozone. Also, the amines and alkanolamines have the potential to contribute to greenhouse gas formation, notably nitrous oxide (N_2_O) and the production of highly carcinogenic compounds and nitrosamines (Schade and Crutzen, 1995; Ge et al., 2011a; Ge et al., 2011b). Hence, elucidating the transformation, mechanisms and kinetics of amines is of potential importance.

The Strecker synthesis, involving a series of chemical reactions, has received great scholarly attention as a potential technique to synthesize chiral α-amino acids using ammonia (NH_3_), hydrogen cyanide (HCN) and aldehyde (RCHO) (Strecker, 1850; Gröger, 2003; Nájera and Sansano, 2007; Wang et al., 2011). However, aminomethanol (NH_2_CH_2_OH), a pivotal intermediate for the synthesis of the simplest amino acid namely, glycine, has not been observed in the laboratories (Nielsen et al., 1979; Schutte et al., 1993). This is may be due to the decomposition of aminomethanol to methanimine (CH_2_NH) and water (H_2_O). Previous theoretical investigations have indicated that aminomethanol is kinetically stable in the gas phase with a substantial barrier of 230 kJ/mol toward dehydration to methanimine (Feldmann et al., 2005). Our recent study also supports that the formation of methanimine and water from aminomethanol is negligibly small and forbidden by an energy barrier of 234 kJ/mol (Ali, 2019). Bossa et al., have observed aminomethanol at low temperatures through the thermal reaction between ammonia and formaldehyde (Bossa et al., 2009). They have also pointed out that aminomethanol may exist in hot corinos in gas phase. The recent experimental study on aminomethanol in astrophysical-like conditions also validates that aminomethanol could be generated from amines on ice grains in proto-stellar cores or protoplanetary disks (Singh et al., 2022). These studies strongly corroborate the existence of aminomethanol in the atmosphere.

**FIGURE 1:**
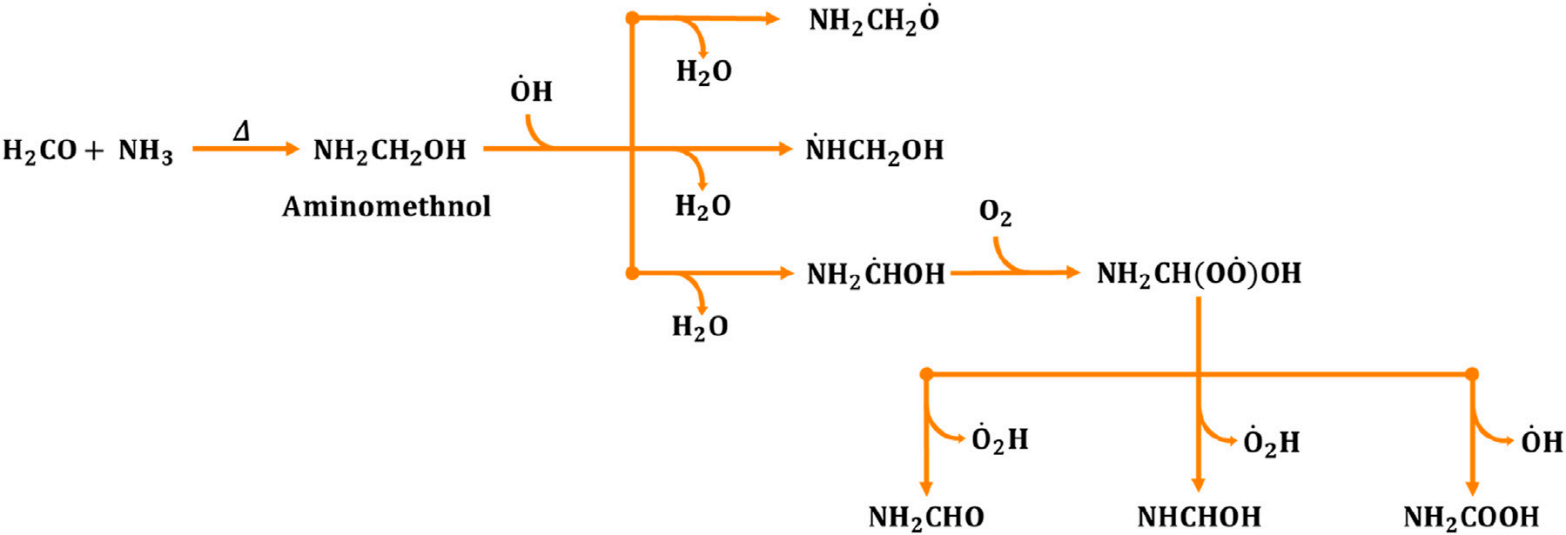
Generalized reaction mechanism for the formation of formamide (NH_2_CHO), formimidic acid (NHCHOH) and amino formic acid (NH_2_COOH) during the aminomethanol + $\dot{O}$ H radical reaction (Ali, 2019).

In our recent study (Ali, 2019), the calculated rate constant of CH_2_O + NH_3_ has suggested that aminomethanol could potentially form at higher temperatures rather than under atmospheric conditions, as illustrated in Figure 1. Additionally, the significant lifetime (∼4 days) of aminomethanol could facilitate the initiation of various chemical reactions with other atmospheric species (Ali, 2019). Nevertheless, the atmospheric chemistry of aminomethanol remains largely unexplored to date, primarily due to the considerable challenges associated with experimental synthesis. Therefore, it is crucial to delve into the reaction mechanisms and kinetics of aminomethanol to evaluate the potential formation of various compounds, including toxicants or carcinogenic byproducts. However, there has been a lack of experimental studies specifically examining the reaction kinetics of the photo-oxidation of aminomethanol. In light of this gap, theoretical approaches, coupled with state-of-the-art computational methodologies and advanced statistical rate theories, are essential for exploring the intricate atmospheric chemistry of aminomethanol, particularly in extreme environmental conditions.

The fate of aminomethanol, in general, is greatly influenced by its gas-phase oxidation by various oxidizing agents. Hydroxyl radicals ( $\dot{O}$ H) are widely present in the atmosphere and the reactions initiated by them are of significant importance among with by various other potential oxidizing agents. It is noteworthy to mention that the higher-order analogue of aminomethanol, namely, monoethanolamine (MEA) (NH_2_CH_2_CH_2_OH), is commercially available and the corresponding gas-phase species are easily formed. Numerous experimental studies have been conducted to characterize MEA and to determine its rate constants (*k*_OH_) with the $\dot{O}$ H radical, along with the resulting products. For example, Murphy and co-workers have evaluated the rate constant, *k*_OH_ of MEA by reacting with the $\dot{O}$ H radical at room temperature. They determined the rate constant to be 7.02 ± 0.46 × 10^−11^ cm^3^ molecule^−1^ s^-1^ (Borduas et al., 2013). Similarly, Onel et al., reported the *k*_OH_ of MEA reacting with $\dot{O}$ H as 7.61 ± 0.76 × 10^−11^, while Karl et al., obtained a value of 9.2 ± 1.1 × 10^−11^ cm^3^ molecule^−1^ s^-1^ (Karl et al., 2012; Onel et al., 2012). These studies demonstrate a rapid reaction between MEA and the $\dot{O}$ H radical, resulting in formamide and formaldehyde as major products, along with the potential formation of hazardous HNCO species. These experimental findings suggest that the initial reaction of MEA with the $\dot{O}$ H, followed by MEA radicals reacting with O_2_, plays a crucial role in determining the atmospheric fate of MEA. In a parallel fashion, the abstraction of a hydrogen atom from NH_2_CH_2_OH by the $\dot{O}$ H radical leads to the formation of three different aminomethanol radicals, including a carbon-centered NH_2_ $\dot{C}$ HOH radical, a nitrogen-centered $\dot{N}$ HCH_2_OH radical and an oxygen-centered NH_2_CH_2_ $\dot{O}$ radical as well as H_2_O, as depicted in Figure 1. It is evident from previous studies on analogous electronic systems that carbon-centered radicals are more prevalent than nitrogen- and oxygen-centered radicals (Xie et al., 2014; Franco et al., 2021). These carbon-centered radicals are subsequently targeted by atmospheric oxygen, leading to the formation of $\dot{O}$ H radical initiated products of aminomethanol namely, formamide (NH_2_CHO), formimidic acid (HN = C(H)-OH) and amino formic acid (NH_2_COOH) in the atmosphere.

Among these product species, formamide is simplest and naturally occurring amide molecule. It is a potential precursor for the synthesis of a broad range of organic molecules (Saladino et al., 2012a; Saitta and Saija, 2014), which are vital for life as it poses all the important elements including carbon C), hydrogen H), oxygen O) and nitrogen N) except the heavy elements like sulphur S) and phosphorus P) (Saladino et al., 2005; 2007; 2012b). Additionally, formamide has an amide functional (-N-C (=O)-) group, which is essential for the formation of the chains of amino acids in order to build up proteins. It promotes the synthesis of four nucleobases namely, adenine, guanine, cytosine and uracil of ribonucleic acid (RNA) (Saladino et al., 2005; Ferus et al., 2015). It also serves as the pre-cursor for carboxylic acids, amino acids and sugars (Botta et al., 2018). These points clearly demonstrating the potential role of formamide in biochemical processes, that are essential for life’s sustenance and evolution.

Also, formamide is a highly ubiquitous molecule in the Universe. It was first detected in space dates back to 1971 when Rubin et al., utilized the 140-foot telescope at the National Radio Astronomy Observatory (NRAO) to scrutinize the Sagittarius B2 region (Sgr B2), situated proximate to the Galactic center (Rubin et al., 1971). Their observations distinctly revealed the three hyperfine components (∆F = 0) of the 2_1,1_–2_1,2_ rotational transition of NH_2_CHO, resonating at 4.62 GHz (6.5 cm). Sgr B2, renowned as the most prodigious star formation region within our Milky Way and marked the maiden identification of an interstellar compound harboring carbon C), hydrogen H), oxygen O), and nitrogen N). Subsequent detections of formamide have been made in diverse astronomical locales, including the vicinity of the young stellar entity W33A (Schutte et al., 1999), within cometary bodies such as C/1995 O1 (Bockelée-Morvan et al., 2000) and C/1996 B2 (Lis et al., 1997) and within the broader expanse of the interstellar medium (Solomon, 1973).

A plethora of experimental and theoretical inquiries have been undertaken to elucidate the formation of formamide along with other organic compounds in the interstellar medium (ISM) by different methods (Woon, 2002; Navarro-González and Raulin, 2004; Pastorek et al., 2019; Ferus et al., 2023). These investigations spanning from the proton irradiation (PI) of gas mixtures containing methane and nitrogen (Koike et al., 2003), ultraviolet irradiation (UV) of ice mixtures containing hydrogen cyanide (HCN), water (H_2_O) and ammonia (NH_3_) (Gerakines et al., 2004), pyrolysis of mixtures comprising carbon monoxide (CO), NH_3_, and H_2_O (Takano et al., 2004) and the photolysis of ices (Bernstein et al., 1997). Similarly, under terrestrial conditions, formamide (NH_2_CHO) can be synthesized from combinations of low molecular weight compounds such as NH_3_, formic acid (HCOOH), formic ester derivatives (HCOOR), CO and alcohols, under both catalyzed and uncatalyzed experimental setups (Deschamps, 1931).

In the current investigation, our focus lies in exploring the intricate mechanistic aspects and branching ratios corresponding to the pivotal stages of the $\dot{O}$ H-initiated oxidation reactions of aminomethanol. The primary objectives of this study are described as follows, 1) unveiling the intricate details about the reaction pathways, energetics and kinetics involved in the interaction of aminomethanol with $\dot{O}$ H radicals and identifying the potential aminomethanol-derived radicals, 2) exploring the reaction pathways, energetics and kinetics involved in the further reactions of aminomethanol-derived radicals with molecular oxygen (O_2_). 3) analyzing the atmospheric implications arising from the exclusive product molecules resulting from the reactions of aminomethanol with OH and O_2_ and 4) conducting a comparative analysis of the pre-reactive complexes, transition states, intermediates, other reactive species and rate constants derived from this study with those reported in previous investigations involving the atmospheric reactions of analogous electronic systems to validate our methodology.

## 2 Theoretical methodology

### 2.1 Electronic structure calculations

All gas-phase *ab initio*/density functional theory (DFT) calculations were performed using the Gaussian 16 suite of programs (Frisch et al., 2019). The stationary points of all molecular systems, including reactants, products, intermediates and transition states on the potential energy surface (PES) were optimized using the Minnesota 2006 exchange correlation functional such as M06-2X, in conjunction with the Pople’s split-valence 6-311++G (3df, 3pd) basis set (Frisch et al., 1984; Zhao and Truhlar, 2008). The long-range van der Waals interactions between the reactive species were accounted using Grimme’s empirical dispersion (GD3) corrections (Grimme et al., 2010). Previous studies have demonstrated that the current level of theory exhibits a reliable performance in addressing noncovalent interactions between gaseous molecules and in locating the transition states of atmospheric and combustion reactions (Ali et al., 2016; 2018; 2022; Ali, 2019; 2020; Dash and Ali, 2022; 2023; Ali and Balaganesh, 2023). Tight convergence criteria were applied during the wave function optimization of the reactive species, complexes, products and transition states. Unscaled vibrational frequencies at the same level of theory (M06-2X/6-311++G (3df, 3pd)) were utilized to compute zero-point energy (ZPE) corrections to the total energies of all molecular systems, to characterize the stationary points on the PES and for rate-constant calculations. Vibrational frequency analysis confirmed all positive frequencies for the reactants, complexes, intermediates and products, while a single imaginary frequency was observed for the transition states.

Additionally, single-point energy calculations were conducted at a higher-level of theory on the molecular structures optimized at a lower-level of theory to ensure an accurate description of the energetic parameters. Specifically, the CCSD(T)/6-311++G (3df, 3pd) level of theory was utilized to estimate the single-point energies of the gas-phase molecular geometries, which were initially optimized at the M06-2X/6-311++G (3df, 3pd) level of theory (Raghavachari et al., 1989). The basis set superposition error (BSSE) calculations were also performed using the counterpoise (CP) corrected method (Boys and Bernardi, 1970; Simon et al., 1996). The <*Ŝ* ^2^> eigenvalues were monitored to evaluate the spin contamination for the wavefunction of the open-shell radicals. The T1-diagnostic values obtained at the CCSD(T)/6-311++G (3df, 3pd) level of theory were analyzed to validate the single-reference method and were found to be within the acceptable range (i.e., ≤0.02) for all important species (Lee and Taylor, 1989). Overall, the combination of CCSD(T)//M06-2X functionals has been employed in numerous research studies, providing a reasonably accurate description of the thermochemistry and chemical kinetics of many atmospheric reactions (Ali et al., 2016; Ali et al., 2018; Ali, 2019; Ali, 2020; Ali et al., 2022; Dash and Ali, 2022; Ali and Balaganesh, 2023; Dash and Ali, 2023).

### 2.2 Chemical kinetic analysis

Comprehensive chemical kinetic calculations for the $\dot{O}$ H initiated oxidation reaction of aminomethanol (AM) using MultiWell suite of codes (Barker, 2001; Barker, 2009; Barker et al., 2023) were performed. This oxidation reaction involves a fast pre-equilibrium between the reactive species such as H_2_NCH_2_OH + $\dot{O}$ H and the pre-reactive complex ${\left[H_{2}\text{NC}H_{2}\text{OH}\cdots \text{OH}\right]}^{•}$ stabilized by the van der Waals forces followed by a second step leading to the respective products as follows,

$$H_{2}{\text{NCH}}_{2}\text{OH}+\dot{O}H\;\begin{array}{c} k_{-1}\\ ⇆\\ k_{1} \end{array}\;{\left[H_{2}\text{NC}H_{2}\text{OH}\cdots \text{OH}\right]}^{•}$$ (1)

$${\left[H_{2}\text{NC}H_{2}\text{OH}\cdots \text{OH}\right]}^{•}\;\vec{k_{2}}\;H_{2}N\dot{C}\text{HOH}+H_{2}O$$ (2)

where, *k*_1_ and *k*_-1_ are the forward and reverse rate constant for the first bimolecular reaction and the *k*_2_ is the rate constant for the second unimolecular reaction. The kinetic rate constants for these bimolecular (*k*, in cm^3^ molecule^−1^ s^-1^) and unimolecular (*k*_uni_, s^-1^) reactions in the high-pressure limit defined by transition state theory are represented as follows,

$$k_{2}=\Gamma \left(T\right)\times \frac{\sigma k_{B}T}{h}\times \frac{Q_{TS}}{Q_{IM}}\times \exp\;\left(-\frac{{∆E}_{0}}{RT}\right)$$ (3)

Assuming that the pre-reactive complex was in equilibrium with the reactants and was at a steady state, then the overall rate constants is expressed as;

$$k=\frac{k_{1}}{k_{-1}+k_{2}}k_{2}$$ (4)

If k_-1_ >> k_2_, the rate constant is rewritten as

$$k=\frac{k_{1}}{k_{-1}}k_{2}$$ (5)

$$k=K_{e}k_{2}$$ (6)

This kinetic model is reasonably correct at the high-pressure limit, where the pre-reactive complex can be stabilized by collisions with other atmospherics species. This approach was widely used in literature for the water-assisted reaction and the predicted rate coefficients are reasonably good agreement with the experimental values (Ali, 2019; Ali et al., 2022, Ali and Balaganesh 2023).

The different parameters of Eqs 3, 6 were breakdown and elaborately discussed the specifics of each component in Supplementary Material to prevent redundancy from earlier research. The *k* and *k*_2_ for the other plausible oxidation reactions of $\dot{O}$ H initiated oxidation reaction of AM such as for H_2_NCH_2_OH+ $\dot{O}$ H→H $\dot{N}$ CH_2_OH + H_2_O and H_2_NCH_2_OH+ $\dot{O}$ H→H_2_NCH_2_ $\dot{O}$ H + H_2_O were also computed.

The temperature- and pressure-dependent microscopic rate constants *k*(E) have also been computed for the O_2_ addition reaction to the aminomethanol radicals generated in Eq. 2. This was accomplished using the Rice−Ramsperger−Kassel−Marcus (RRKM)/master equation (ME) theory, implemented in the MultiWell suite of programs. The MultiWell code facilitates the computation of non-steady-state effects including unimolecular decomposition processes, isomerization, collision energy transfer and chemical activation for the complex rate-constant calculations. To perform these calculations, molecular and energetic parameters such as vibrational frequencies, moments of inertia and reaction barriers are required as input data. Using this data, the MultiWell suite computes sum and density-of-states, followed by the evaluation of microscopic rate-constant *k*(E)*.* The RRKM/ME microscopic rate-constant *k*(E) is defined as follows,

$$k\left(E\right)=\left[\frac{m^{\neq }}{m}\frac{\sigma_{ext}}{\sigma_{ext}^{\neq }}\right]\frac{g_{e}^{\neq }}{g_{e}}\frac{1}{h}\frac{G^{\neq }\left(E-E_{0}\right)}{\rho \left(E\right)}.$$ (7)

The details of each term can be found in the Supplementary Material. Temperature and pressure-dependent rate constants and branching ratios of the products were evaluated by incorporating N_2_ gas as the bath gas. The collisional energy transfer process was addressed using the conventional temperature-dependent exponential-down model with a <ΔE > _down_ parameter (which represents the average energy loss per the collision of the active compound with the bath gas molecule), with an approximate value of ∼200*(T/300)^0.85^ cm^−1^ (Goldsmith et al., 2012). Lennard-Jones (L-J) parameters were employed to account for the frequency of collisions between the active compound and the bath gas (N_2_) collider. The L-J parameters for N_2_ gas, specifically σ (N_2_) = 3.74 Å and ε/k_B_ (N_2_) = 82 K, were sourced from the literature, while the same parameters for all wells were adopted from our previous study (Dash and Ali, 2022).

For the barrierless reactions i.e., $\dot{\text{AM}}$ +O_2_→AM-O $\dot{O}$ and product complexes to individual product molecules Inverse Laplace Transform (ILT) method was incorporated to determine the rate-coefficients (Robertson et al., 1995). Consistent with studies on numerous analogous reactions, this method has proven effective, with the Arrhenius’s activation energy equating to the critical energy of the reaction (*E*_0_) (Firaha et al., 2018). Additionally, statistical rate theories that neglect non-statistical effects were applied, including slow intramolecular vibrational energy redistribution (IVR) as discussed in previous study (Mazarei and Barker, 2022).

The pressure-dependent total rate constants *k* ^*bimol*^ (*T, M*) for aminomethanol radical ( $\dot{\text{AM}}$ ) + O_2_ have been computed using,

$$k^{bimol}\left(T,M\right)={\Gamma \;K}_{eq}\times k_{\infty }^{uni}\left(1-f_{\dot{\text{AM}}+O_{2}}\right)$$ (8)

where, Γ represents the quantum mechanical tunneling corrections, $f_{\dot{\text{AM}}+O_{2}}$ is the branching fraction ( *f* ) of the chemical reaction returning to the respective reactive species and $k_{\infty }^{uni}$ is the rate constant at the high-pressure limit. The tunneling was incorporated for the chemical activation distribution in all our chemical kinetic calculations using the keywords “CHEMACT” and “TUN”.

Finally, the calculated rate constants were fitted at the high-pressure limit ( $k_{\infty }$ ) in the temperature range of 200–400 K to the modified Arrhenius expression, which is as follows,

$$k_{\infty }\left(T\right)=A\times T^{n}\times \exp\left(\frac{-E_{a}}{RT}\right)$$ (9)

where A is the pre-exponential factor, T is the temperature, *n* is the temperature exponent and *E*_a_ is the activation energy. The coordinates of equilibrium geometries, vibrational harmonic frequencies and rational constants of all important species involved in the $\dot{O}$ H initiated oxidation reaction of AM are listed in the Supplementary Material.

## 3 Results and discussion

### 3.1 Rotational conformers of aminomethanol

**FIGURE 2:**
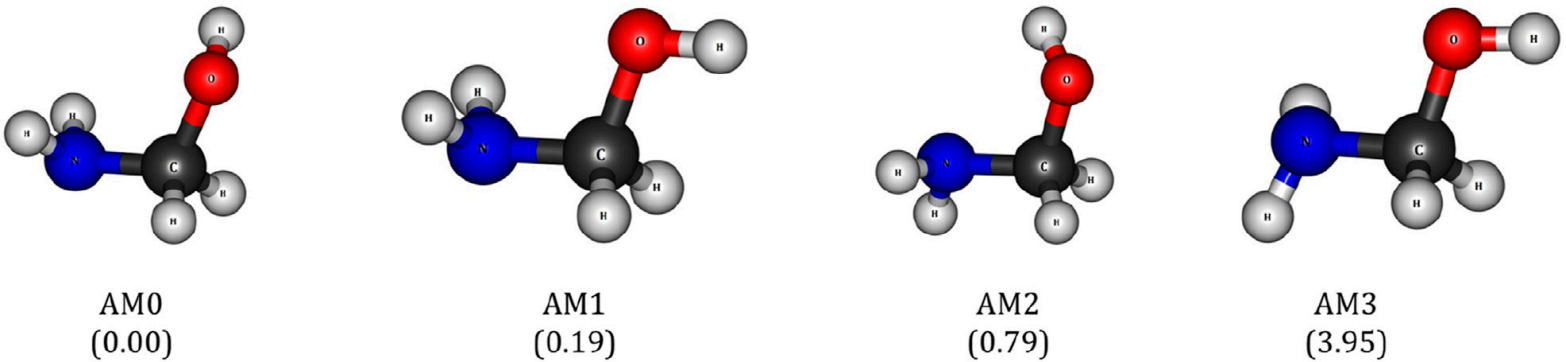
Different rotational conformations of aminomethanol (AM). The values in the parenthesis indicate the relative energies in kcal/mol.

The oxidation reaction between the aminomethanol (AM) and $\dot{O}$ H radical occur through the abstraction of H-atom from three different H-bearing functional groups (*i.e.*, –CH_2_, –NH_2_ and –OH) of AM. Generally, the H atom of –CH_2_, –NH_2_ and –OH groups along with the presence of lone pair of electrons on the N and O atoms facilitate the hydrogen bonding (H-bonding) between the AM and the $\dot{O}$ H radical. Interestingly, the –NH_2_ and –OH groups of AM can freely rotate around the single bonds to form the inter molecular H-bonding with the $\dot{O}$ H radical based on its attacking direction. It leads to different rotational conformations for the AM- $\dot{O}$ H radical complex. In addition, formation of such intermolecular H-bonding in the pre-reactive and the transitions state (TS) structures has a great effect on the energetics of the reaction. Hence, we have comprehensively explored the different reaction pathways and the corresponding energy barriers using different rotational conformations of AM. It is evident from previous studies that the AM exhibits four different rotational conformations, which we named as AM0, AM1, AM2 and AM3 as presented in Figure 2 (Hays et al., 2013). It shows that the AM0 is energetically more stable and can be consider as ground state conformation of AM. The geometrical features of ground state conformation of AM are good in agreement with that of previous study (Hays et al., 2013). Other rotational conformations such as AM1, AM2 and AM3 exhibit ∼0.2, 0.8 and 4 kcal/mol higher energy compared to the AM0, respectively. The trend in the conformational stabilities of AM is in accordance with the study of Weaver and co-workers (Hays et al., 2013). Hence, the AM0 is used as a reference to provide the further intricate details about the mechanistic pathways of the AM oxidation reaction with the $\dot{O}$ H radical.

### 3.2 Potential energy surface of aminomethanol + $\dot{O}$ H

**FIGURE 3:**
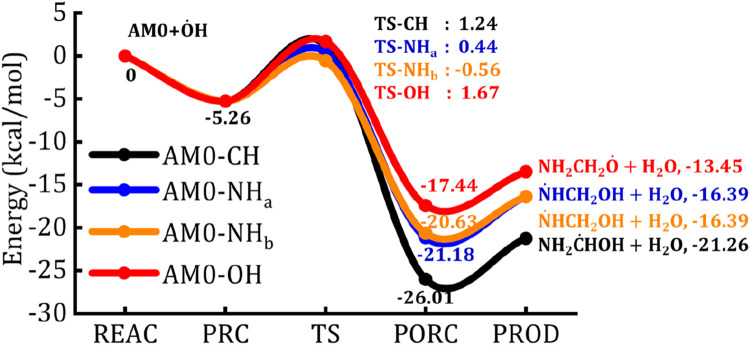
The zero-point energy corrected potential energy surface of AM0 + $\dot{O}$ H radical.

**FIGURE 4:**
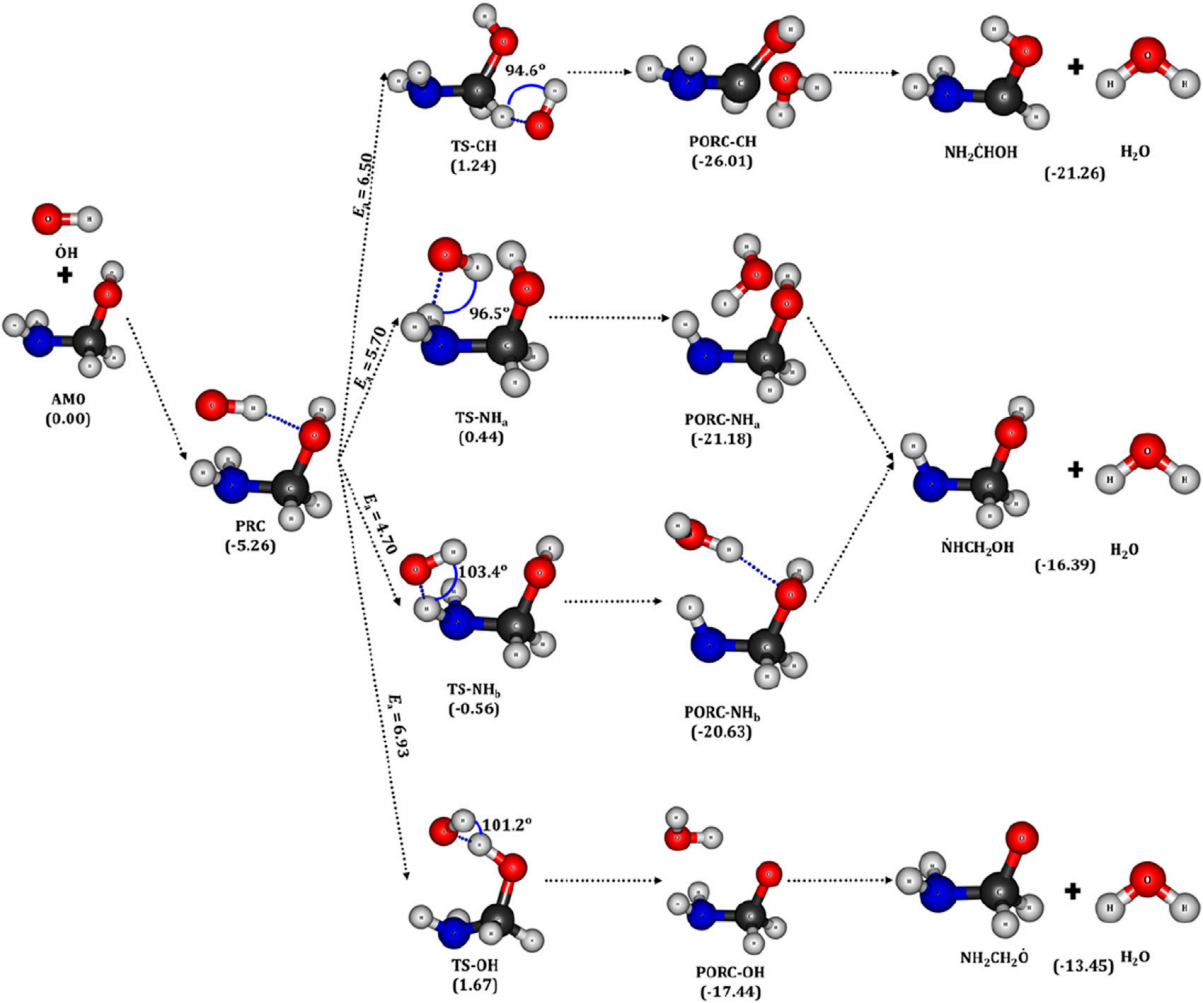
Optimized geometries of aminomethanol (AM0), PRC, TSs, PORCs and the radicals of $\dot{O}$ H initiated AM0 oxidation reaction. All the values in the parenthesis are in kcal/mol.

The zero-point energy (ZPE) corrected potential energy surface (PES) for the H-abstraction reaction of the most stable conformation of aminomethanol (AM0) by the $\dot{O}$ H radical is depicted in Figure 3. In this reaction, the $\dot{O}$ H radical abstracts the H-atom from three different H-bearing moieties ( i . e ., –CH_2_, –NH_2_ and –OH) of AM0, leading to the formation of three distinct AM0 radicals, namely, the carbon-centered NH_2_ $\dot{C}$ HOH radical, nitrogen-centered $\dot{N}$ HCH_2_OH radical and oxygen-centered NH_2_CH_2_ $\dot{O}$ radical. These radicals are obtained through an energetically favorable pre-reactive complex (PRC), followed by the transition states (TSs) for the transfer of the H-atom. The optimized geometries of AM0, $\dot{O}$ H radical, PRC, TSs, post-reactive complexes (PORCs) and the final radical species of $\dot{O}$ H initiated AM oxidation reaction are presented in Figure 4. In detail, the $\dot{O}$ H radical attacks the AM0 and forms a PRC, which is stabilized by the formation of H-bonding between the H atom of $\dot{O}$ H radical and the O atom of AM0 as shown in Figure 4. The PRC is stabilized with respect to the reactants by −5.26 kcal/mol. Subsequently, the H-bonding stabilized $\dot{O}$ H radical rearranges in the reactive space of AM0 in a way to form a bond angle between the H- atom of –CH_2_, –NH_2_ and –OH groups and the $\dot{O}$ H radical close to that of H_2_O molecule, to viably eliminate as a H_2_O molecule through a transition state.

For example, the bond angle between the H atom of the –CH_2_ group and $\dot{O}$ H is found to be 94.6° ( i . e ., ∡HC-H··· $\dot{O}$ H = 94.6°) in the TS-CH (see Figure 4). The presence of two inequivalent H-atoms on –NH_2_ group form two different TSs. The TSs associated with the back and front H-atoms are designated as TS-NH_a_ and TS-NH_b_, respectively. The bond angles are found to be ∡H_b_N-H_a_··· $\dot{O}$ H = 96.5°, ∡H_a_N-H_b_··· $\dot{O}$ H = 103.4° and ∡O-H··· $\dot{O}$ H = 101.2° in the TS-NH_a_, TS-NH_b_ and TS-OH, respectively. Computed results reveal that the abstraction of H-atom from the –NH_2_ group through the transition state, TS-NH_b_ (TS-NH_a_) is more favorable with an energy barrier of 4.7 (5.7) kcal/mol followed by the abstraction from the –CH_2_ (6.5 kcal/mol) and –OH (6.93 kcal/mol) groups. The H transfer through the transition states namely, TS-CH, TS-NH_a_, TS-NH_b_ and TS-OH forms the corresponding post-reactive complexes of water molecule and AM0 radicals, which are stabilized by −26, −21, −21 and −17 kcal/mol with respect to the reactants, respectively. Finally, these post-reactive complexes separate into the AM0 radical and water molecules in each reaction pathway.

To account for the effect of other rotational conformers on the oxidation reaction, the energies of PRCs, TSs and PORCs have been computed for the abstraction of H atom from –CH_2_, –NH_2_ and –OH groups of other rotational conformations of AM (*i.e.*, AM1 and AM2). The complete reaction profile for the H-abstraction reaction of AM1 along with the energies is presented Supplementary Figure S1. The H abstraction from the –CH_2_ group of AM1 proceeds *via* three TSs such as TS-CH_a_ TS-CH_b_ and TS-CH_c_. Among these TSs, TS-CH_a_ is linked to the PRC1 while TS-CH_b_ and TS-CH_c_ are linked to the PRC2 (see Supplementary Figure S2). However, all these–CH TSs are linked to the same product complex and lead to the formation of a single NH_2_ $\dot{C}$ HOH radical conformation. On the other hand, the abstraction of H from –NH and –OH groups of AM1 occurs *via* the TSs namely, TS-NH and TS-OH, which are linked to the pre-reactive complex, PRC1. These TSs of AM1 subsequently form the N- and O- centered radicals similar to that of AM0+ $\dot{O}$ H radical reaction. Similarly, we evaluated the energetics of all important species of AM2+ $\dot{O}$ H oxidation reaction and the reaction profile is shown in Supplementary Figure S3. The optimized geometries of the reactive species, intermediates, TSs, post-reactive complexes and C-, N-, O-centered radicals of AM2+ $\dot{O}$ H reactions are presented in Supplementary Figure S4.

Finally, the energetics and the barrier height values of H abstraction reactions from AM0, AM1 and AM2 by $\dot{O}$ H radical have been compared and presented in Supplementary Table S1. It shows that the energies of PRCs of AM0, AM1 and AM2 are in the range of −4.6 to −5.8 kcal/mol. These values are in good agreement with the PRC energies of similar electronic systems. For example, Franco et al., have investigated the abstraction of H atom from the different conformers of methanediol (Franco et al., 2021). They showed that, the PRC between OHCH_2_OH and $\dot{O}$ H is stabilized by −5.22 kcal/mol with respect to the reactants in the most stable conformation of methanediol. Ali et al., have studied the $\dot{O}$ H + CH_3_OH reaction profile under tropospheric conditions (Ali et al., 2021). They showed that the H abstraction occurred through a hydrogen bond stabilized PRC, which exhibits a relative energy of −4.97 kcal/mol. Du and co-workers have explored the gas-phase reaction of ethanol with $\dot{O}$ H radical (Xu et al., 2019). Results of this study reveal that the PRC between $\dot{O}$ H radical and CH_3_CH_2_OH is stabilized by the H-bonding. It exhibits a relative energy of −5.2 kcal/mol. On the other hand, González et al. have explored the rate constants for the $\dot{O}$ H radical reaction with CH_3_NH_2_ using experimental and theoretical methods (González et al., 2022). They have found that the PRC between $\dot{O}$ H radical and CH_3_NH_2_ is stabilized through formation of H-bond between the N(CH_3_NH_2_) and H(OH). It exhibits a relative energy of −6.69 kcal/mol similar to that of PRC1 (−5.8 kcal/mol) formed with the conformer AM1. Clearly, the energies of PRCs formed between $\dot{O}$ H radical and different rotational conformers of AM are in close agreement with those of previous studies. The computed barrier height values for the abstraction of H-atom from the different moieties of AM by $\dot{O}$ H radical are closely corroborating with that of abstraction of H from the respective moieties of similar electronic systems. For example, Franco et al., have reported the barrier height values in the range of 6.03–6.95 and 8.25–10.22 kcal/mol for the abstraction hydrogen from the –CH_2_ and –OH groups of methanediol, respectively (Franco et al., 2021). Similarly, Ali et al., and Xu et al., have reported the barrier height values around 5.8 (7.5) and 5.8 (6.8) kcal/mol for H abstraction from –CH (–OH) groups of methanol and ethanol, respectively (Xu et al., 2019; Ali et al., 2021). Onel et al., and Tian et al., have reported the barrier height values for –NH abstraction of alkylamines around 4.4-9.5 kcal/mol (Tian et al., 2009; Onel et al., 2013). Our computed results show that the barrier height values for the abstraction of H from –CH_2_, –NH_2_ and –OH groups are in the range of 4.12–6.50, 3.76–5.50 and 6.5–9.25 kcal/mol, respectively. These results also corroborate that the barrier heights are good in agreement with the previous studies. This analysis not only provides more confidence but also substantiate our choice of *ab initio* and DFT methods.

It is worth to mention here that, the TS energies for the major H-abstraction pathways i.e., from the –CH_2_ and –NH_2_ channels of NH_2_CH_2_OH + $\dot{O}$ H radical reaction are found to be around 1-2 kcal/mol. Previous studies show that the $\dot{O}$ H radical initiated H-abstraction reactions from different reactants are favorable even with the slight positive TS energies. For example, Nguyen et al., have studied the H-abstraction from the CH_3_OH using *ab initio*/RRKM methods (Nguyen et al., 2019). They reported the TS energies for H-abstraction around 2.3 and 0.5 kcal/mol. Further, they satisfactorily reproduced the experimental rate constants using these TS energies. Baidya et al., have explored the H-abstraction reaction of CHF_2_CH_2_OH with $\dot{O}$ H radical to unveil the atmospheric implications of chlorofluorocarbons (Baidya et al., 2018). All the TSs reported in this study exhibit positive energies in the range of 0.8–3.1 kcal/mol. However, they reported that their calculated *k*_OH_ value is in good agreement with the experimental value. Rahbar et al., have investigated the kinetics and mechanism of the $\dot{O}$ H radical initiated atmospheric oxidation of catechol over the temperature range 200-400 K (Rahbar et al., 2021). However, catechol shows high positive TS values (1.27–8.8 kcal/mol) for the H-abstraction reaction. The authors pointed out that the computed rate constants are close to that of experimental value. In light of these analogous studies, we believe that the slight positive energies (∼1-2 kcal/mol) for the TSs are acceptable within the troposphere, which is the focal point of interest in our present investigation.

**FIGURE 5:**
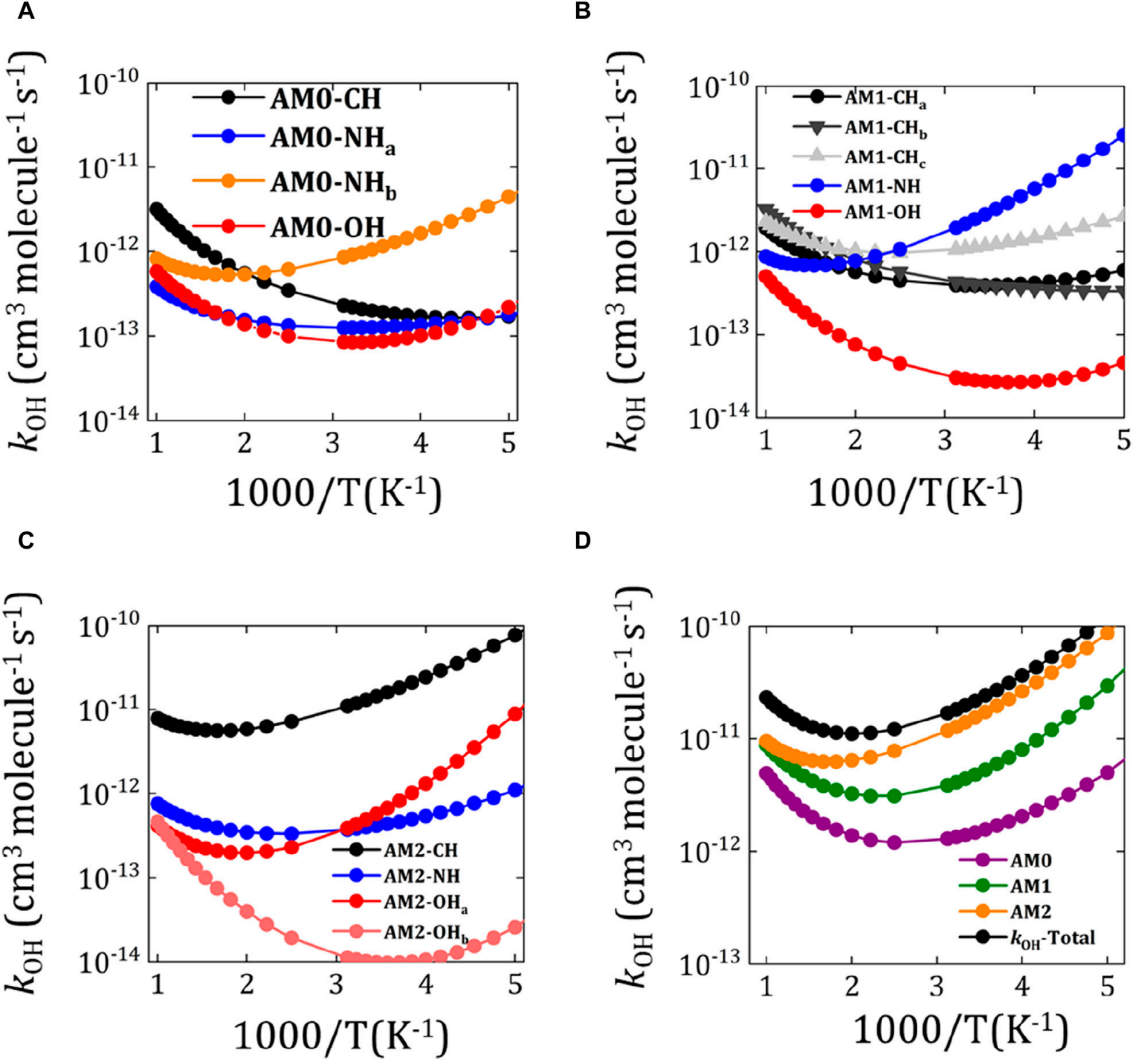
Calculated rate constants ( $k_{\text{OH}}$ ) for the abstraction of H from –CH_2_, –NH_2_ and –OH groups of different rotational conformations of aminomethanol. **(A)** AM0, **(B)** AM1 and **(C)** AM2. **(D)** The total rate constants from each channel of AM0, AM1 and AM2.

The rate constant ( $k_{\text{OH}}$ ) and the branching fraction values have been computed for the hydrogen abstraction reactions at 300 K using all possible conformations. The $k_{\text{OH}}$ values for the abstraction of H from different channels (–CH_2_, –NH_2_ and –OH) of AM0 are plotted in Figure 5A and listed in Supplementary Table S2. The sum of the rate constant values from different channels of AM0 (*i.e.*, *k*_OH_-AM0) are presented in Table 1. This data indicates that the rate constants for H abstraction from different channels gradually decrease within the temperature range 200–400 K. The *k*_OH_-AM0 value for $\dot{O}$ H radical reaction with AM0 rotational conformation is found to be 1.40 × 10^−12^ cm^3^ molecule^−1^ s^−1^ at 300 K. Similarly, the $k_{\text{OH}}$ for each H abstraction channel of AM1 and AM2 has been computed and plotted in Figures 5B, C and the numerical values are presented in Supplementary Tables S3, S4. The sum of the rate constant values from different channels of AM1 and AM2 (*i.e.*, *k*_OH_-AM1 and *k*_OH_-AM1) has been found to be 4.40 × 10^−12^ and 1.39 × 10^−11^ cm^3^ molecule^−1^ s^−1^, respectively at 300 K (see Table 1). This results in an overall rate constant for the hydrogen abstraction from the all AM conformations to be around 1.97 × 10^−11^ cm^3^ molecule^−1^ s^−1^ (see Figure 5D; Table 1). The computed total *k*_OH_ value of AM is closely aligns with that of CH_3_NH_2_ (1.97 × 10^−11^ cm^3^ molecule^−1^ s^−1^) (Onel et al., 2013), CH_3_NHCH_3_ (6.27 × 10^−11^ cm^3^ molecule^−1^ s^−1^) (Onel et al., 2013), CH_3_CH_2_NH_2_ (2.50 × 10^−11^ cm^3^ molecule^−1^ s^−1^) (Onel et al., 2013), NH_2_CH_2_CH_2_OH (7.27 × 10^−11^ cm^3^ molecule^−1^ s^−1^) (Xie et al., 2014), CH_3_CH_2_OH (9.06 × 10^−11^ cm^3^ molecule^−1^ s^−1^) at 300 K. The *k*_OH_ values are also computed using basis set super position error (BSSE) corrections. It is evident from Supplementary Table S5 that the BSSE corrections does not altered the rate constants appreciably. Overall, a negative temperature dependence in the *k*_OH_ of AM has been observed over the temperature range 200–400 K as can be seen from Figure 5D, similar to that of amines and alcohol reactions. The energetic values coupled with the computed rate constant results clearly align with those of previous studies, which further corroborating the reliability of the computational methodology and the scheme adopted in this study. Additionally, the branching fraction values for the abstraction of H from –CH_2_, –NH_2_ and –OH groups to successively form product radicals NH_2_ $\dot{C}$ HOH, $\dot{N}$ HCH_2_OH and NH_2_CH_2_ $\dot{O}$ are estimated to be around 77%, 20% and 3%, respectively. Xie et al., have shown that the branching ratio is around 82%, 17% and 1% for the abstraction of H from –CH_2_, –NH_2_ and –OH groups of monoethanolamine (MEA) (Xie et al., 2014). The computed branching fraction values of AM are in good agreement with that of MEA (Xie et al., 2014). The branching fraction values clearly suggest a strong preference for the C-centered H-abstraction over N- and O- centered H-abstraction. Interestingly, the computed branching fraction values also strongly adhere to the Evans−Polanyi relationship bond enthalpy of O−H > N−H > CH relationship (Evans and Polanyi, 1938).

**TABLE 1:** Calculated temperature dependent rate constants (*k*_OH_, cm^3^ molecule^−1^ s^−1^) for the $\dot{O}$ H initiated oxidation reaction of aminomethanol using the rotational conformations, AM0, AM1 and AM2. *k*_OH_-Total is the total rate constant using all the conformations.

Temp	*k*_OH_-AM0	*k*_OH_-AM1	*k*_OH_-AM2	*k*_OH_-total
200	5.00 × 10^−12^	2.95 × 10^−11^	8.70 × 10^−11^	1.22 × 10^−10^
210	3.92 × 10^−12^	2.08 × 10^−11^	6.37 × 10^−11^	8.85 × 10^−11^
220	3.19 × 10^−12^	1.55 × 10^−11^	4.87 × 10^−11^	6.74 × 10^−11^
230	2.68 × 10^−12^	1.20 × 10^−11^	3.86 × 10^−11^	5.33 × 10^−11^
240	2.32 × 10^−12^	9.62 × 10^−12^	3.15 × 10^−11^	4.34 × 10^−11^
250	2.04 × 10^−12^	7.99 × 10^−12^	2.63 × 10^−11^	3.64 × 10^−11^
260	1.84 × 10^−12^	6.81 × 10^−12^	2.25 × 10^−11^	3.11 × 10^−11^
270	1.69 × 10^−12^	5.94 × 10^−12^	1.95 × 10^−11^	2.71 × 10^−11^
280	1.56 × 10^−12^	5.29 × 10^−12^	1.72 × 10^−11^	2.41 × 10^−11^
290	1.47 × 10^−12^	4.79 × 10^−12^	1.54 × 10^−11^	2.17 × 10^−11^
300	1.40 × 10^−12^	4.40 × 10^−12^	1.39 × 10^−11^	1.97 × 10^−11^
310	1.34 × 10^−12^	4.10 × 10^−12^	1.27 × 10^−11^	1.82 × 10^−11^
320	1.29 × 10^−12^	3.86 × 10^−12^	1.18 × 10^−11^	1.69 × 10^−11^
400	1.19 × 10^−12^	3.10 × 10^−12^	7.78 × 10^−12^	1.21 × 10^−11^
450	1.26 × 10^−12^	3.09 × 10^−12^	6.86 × 10^−12^	1.12 × 10^−11^
500	1.38 × 10^−12^	3.24 × 10^−12^	6.43 × 10^−12^	1.10 × 10^−11^
550	1.55 × 10^−12^	3.49 × 10^−12^	6.26 × 10^−12^	1.13 × 10^−11^
600	1.76 × 10^−12^	3.82 × 10^−12^	6.27 × 10^−12^	1.18 × 10^−11^
650	2.01 × 10^−12^	4.22 × 10^−12^	6.40 × 10^−12^	1.26 × 10^−11^
700	2.30 × 10^−12^	4.68 × 10^−12^	6.63 × 10^−12^	1.36 × 10^−11^
750	2.63 × 10^−12^	5.21 × 10^−12^	6.94 × 10^−12^	1.48 × 10^−11^
800	3.00 × 10^−12^	5.80 × 10^−12^	7.32 × 10^−12^	1.61 × 10^−11^
850	3.41 × 10^−12^	6.45 × 10^−12^	7.77 × 10^−12^	1.76 × 10^−11^
900	3.87 × 10^−12^	7.17 × 10^−12^	8.27 × 10^−12^	1.93 × 10^−11^
950	4.38 × 10^−12^	7.95 × 10^−12^	8.83 × 10^−12^	2.12 × 10^−11^
1000	4.93 × 10^−12^	8.80 × 10^−12^	9.45 × 10^−12^	2.32 × 10^−11^

The bimolecular reactions between the aminomethanol (NH_2_CH_2_OH) + $\dot{O}$ H radical and the NH_2_ $\dot{C}$ HOH + O_2_ would also be possible in their excited electronic states. It is evident from previous studies that, the computationally characterized potential energy surface (PES) and the corresponding rate constants of (for example, ethyl alcohol (H_3_C-CH_2_-OH) (Xu et al., 2019), monoethanolamine (NH_2_CH_2_CH_2_OH) (Xie et al., 2014), methanediol (OH-CH_2_-OH) (Franco et al., 2021)) similar electronic systems agrees well with that of experimental studies. However, these studies not included any excited state photochemical interactions to obtain the rate constants. Further, Al-Hashimi and co-workers reported that the interactions between the O-Anisidine and $\dot{O}$ H radical occurs in the ground state rather than in the excited state (Abdel-Rahman et al., 2021). Priya et al., have explored the abstraction reaction mechanism of $\dot{O}$ H radical with 2-methoxyphenol (Priya and Lakshmipathi, 2017). They showed that the H-abstraction reaction can occur in ground state than the in the excited state. Similar to these studies, we believe that the interactions between the aminomethanol and the gaseous species could be corresponding to ground state rather than to excited state.

In recent decades, significant advances in atmospheric chemistry have spurred the development of new theoretical approaches for exploring intricate details of ground-state chemical reactions and their underlying mechanisms. Nevertheless, an equivalent synergy between theory and experimentation remains absent in the realm of atmospheric photochemistry involving electronically excited states. The modeling of molecular photochemistry necessitates a meticulous consideration of non-adiabatic effects, specifically, the coupling between electronic states and molecular motion. This presents formidable challenges, as it contradicts several conventional approximations in theoretical chemistry. Notably, non-adiabatic effects challenge the venerable Born-Oppenheimer approximation, while classical treatments of nuclear dynamics may prove inadequate and non-equilibrium phenomena can challenge established reaction rate theories.

A plethora of methodologies have emerged to address these challenges, including MCTDH (Multi Configuration Time Dependent Hartree) (Manthe et al., 1992; Beck, 2000; Meyer, 2012), trajectory surface hopping (TSH) (Tully and Preston, 1971; Hammes-Schiffer and Tully, 1994; Fabiano et al., 2008) and *ab initio* multiple spawning (AIMS) (Ben-Nun et al., 2000; Ben-Nun and Martínez, 2002; Yang and Martínez, 2011). However, the application of these techniques to investigate atmospheric photochemistry encounters various challenges. These encompass complexities in simulating spectroscopically relevant choices and atmospheric modeling interests, the intricate electronic architecture of multichromophoric volatile organic compounds (VOCs), the diverse excited-state dynamics triggered by solar irradiation, the protracted dynamics of excited VOCs, the prominence of intersystem crossings or collisional processes and the modulating influence of aqueous environments such as those found in atmospheric aerosols and clouds. Given that non-adiabatic dynamic approaches demand considerable computational resources beyond our current capacity and each method carries its own limitations, we envision these studies as prospects for future exploration.

### 3.3 Potential energy surface of NH_2_ $\dot{C}$ HOH radical + ^3^O_2_

**FIGURE 6:**
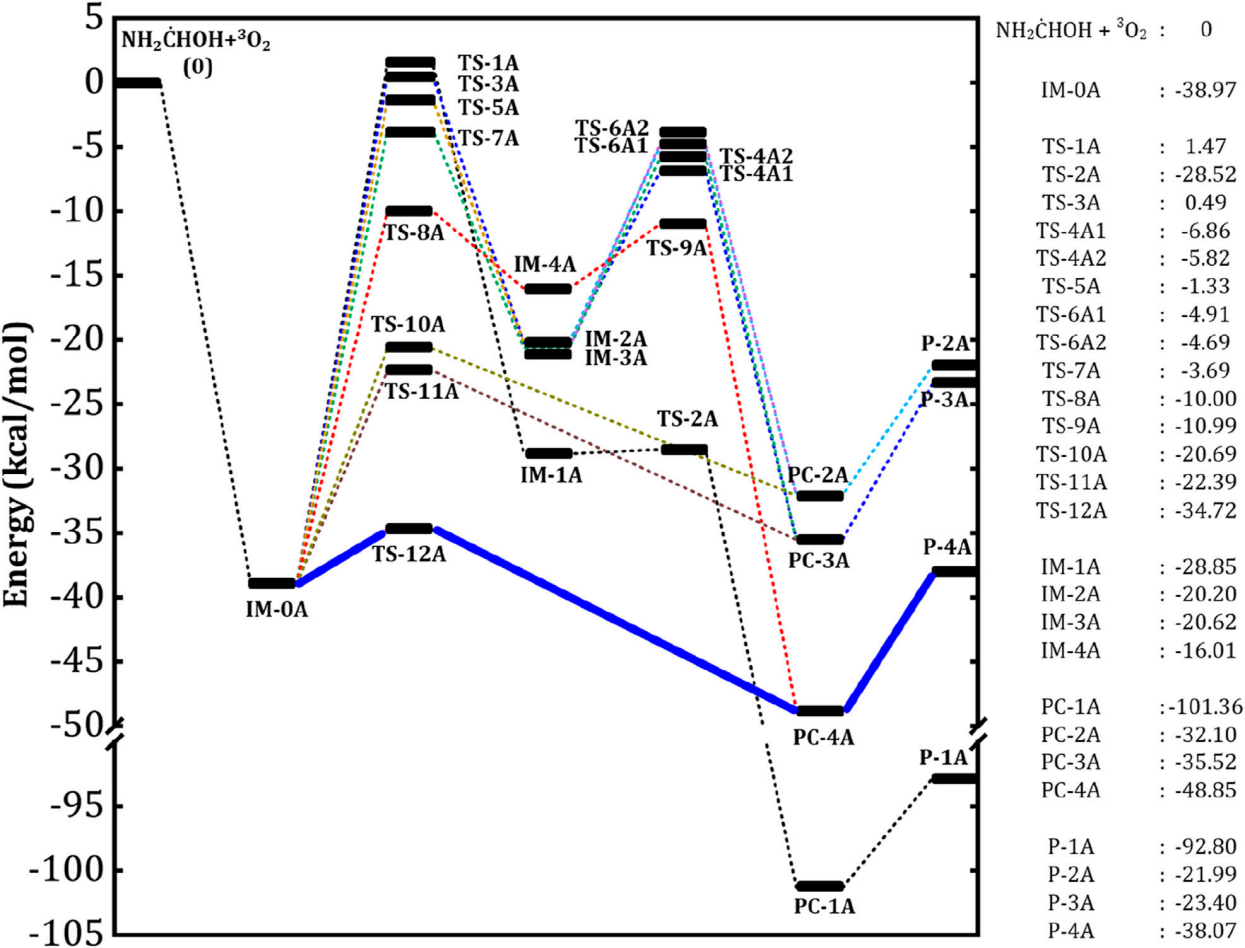
The zero-point energy (ZPE) corrected potential energy surface of AM radical (NH_2_ $\dot{C}$ HOH) + ^3^O_2_ radical. All the energy values are in kcal/mol.

**FIGURE 7:**
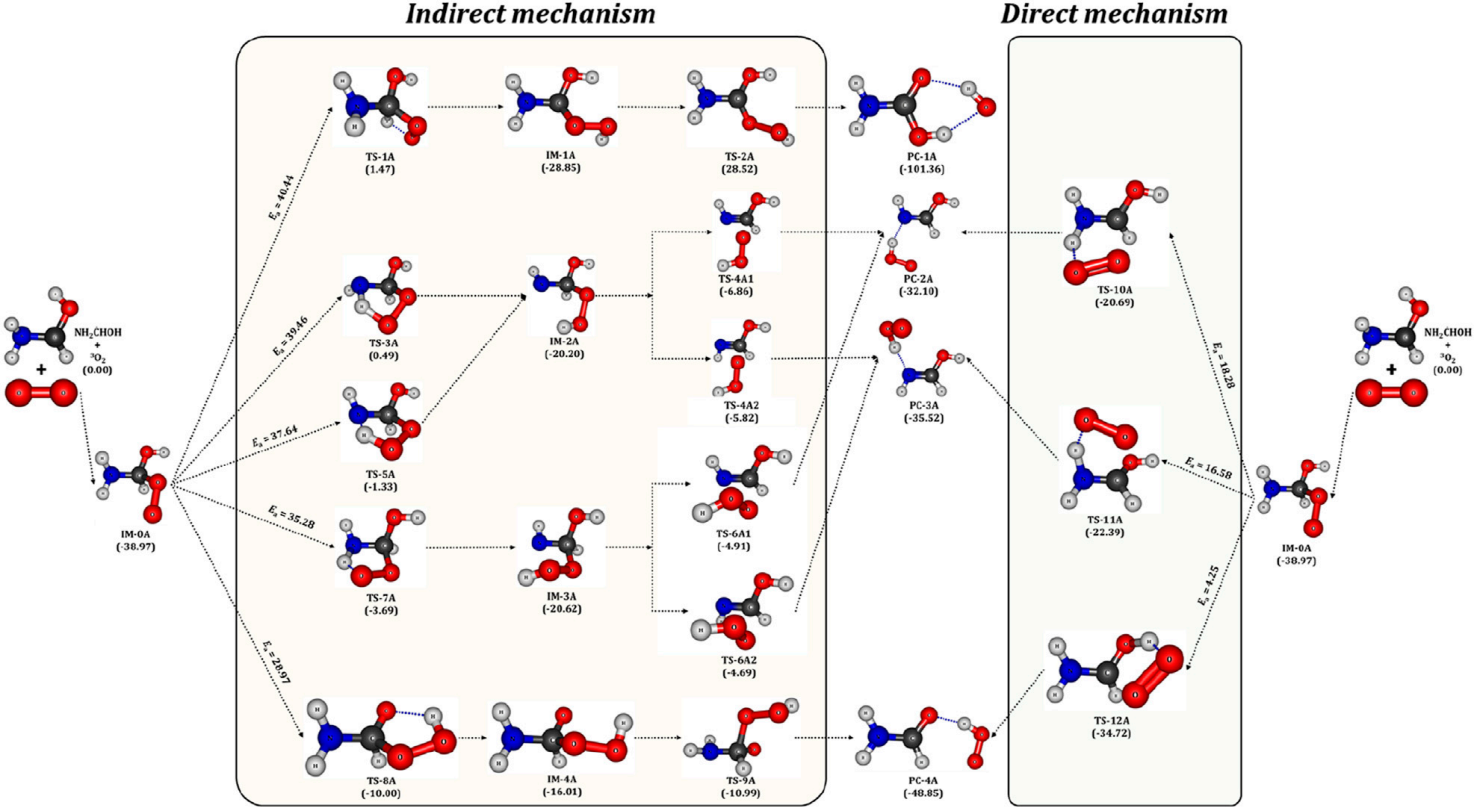
Optimized geometries of intermediates (IMs), transition states (TSs) and product complexes (PCs) of ^3^O_2_ + NH_2_ $\dot{C}$ HOH reaction. All the values are in kcal/mol.

The aminomethanol radicals produced in the initial reactions of aminomethanol + $\dot{O}$ H are highly reactive and undergoes subsequent reactions with atmospheric O_2._ The NH_2_ $\dot{C}$ HOH has been considered for further reactions with atmospheric oxygen due to its large branching fraction. The zero-point energy (ZPE) corrected potential energy surface of NH_2_ $\dot{C}$ HOH + O_2_ reaction has been computed and shown in Figure 6, while the optimized geometries of intermediates, transition state structures and the product complexes (PCs) are depicted in Figure 7. The triplet oxygen molecule (^3^O_2_) reacts with NH_2_ $\dot{C}$ HOH radical and barrierlessly added to the C-site to form a peroxy radical (IM-0A) intermediate, as shown in Figure 6. However, the attacking direction of O_2_ molecule on NH_2_ $\dot{C}$ HOH and the feasibility for the rotation of –NH_2_ and –OH groups around the CO and CN single bonds lead to the formation of different peroxy radical intermediate rotational conformers. Similar to aminomethanol, the IM-0A also exhibits other rotational conformations, for example, IM-0B and IM-0C as presented in Supplementary Figure S5. Previous studies have shown that the rotational conformations of the peroxy radical have a negligible effect on the formation of the end products (Xie et al., 2014). Hence, we have chosen the highly stable NH_2_C(O $\dot{O}$ )HOH radical (IM-0A) for further studies and the reactions between other rotational conformations and O_2_ have been excluded. The IM-0A strongly stabilized and located well below the reactants with a relative energy of −39 kcal/mol with respect to the reactants. The transfer of H occurred from −CH, –NH_2_ and –OH groups to the O-site within the peroxy radical intermediate (IM-0A). We consider the H transfer followed by the breaking of CO or OO bonds occurs through two consecutive steps as an indirect mechanism while the same occurs in a single step as a direct mechanism. Overall, five indirect (*via* TS-1A, TS-3A, TS-5A, TS-7A and TS-8A) and three direct reaction paths (*via* TS-10A, TS-11A and TS-12A) have been studied for the NH_2_ $\dot{C}$ HOH + O_2_ reaction as shown in Figure 7.

In detail, the transfer of H atom from –CH and –OH groups to the O-site of peroxy radical (IM-0A) is favored through the transition states TS-1A and TS-8A. On the other hand, the presence of two different H atoms in –NH_2_ group and different attacking directions of ^3^O_2_ lead to three different reaction pathways, which proceed through the TSs, TS-3A, TS-5A and TS-7A for the H abstraction from –NH_2_ group. Among all these TSs, the TS-1A and TS-3A are situated above the reactants with an energy of 1.5 and 0.5 kcal/mol followed by TS-5A, TS-7A and TS-8A (−1.3, −3.7 and −10.0 kcal/mol). It indicates that the H-transfer from –CH group to O-site of IM-0A requires a high energy of ∼40 kcal/mol to form the intermediate IM-1A. The transfer of two different H atoms from –NH_2_ group respectively requires 40 (*via* TS-3A) and 35 (*via* TS-7A) kcal/mol to form the intermediates, IM-2A and IM-3A. Further, different spatial arrangement of O_2_ also facilitates a distinct transition state, TS-5A for the H transfer from –NH_2_ group and it leads to the previous intermediate, IM-2A. The reaction proceed through TS-5A shows a barrier height more than 35 kcal/mol. The H transfer process from that of –OH group requires a relatively low energy of 29 kcal/mol *via* the TS-8A to form IM-4A. The barrier heights for these H-transfer reactions strongly comply with that of H transfer from the similar functional groups. The intermediates IM-1A to IM-4A are stabilized by ∼ -29, −20.2, −20.6 and −16 kcal/mol with respect to the reactants, respectively. The breaking of O-O and C-O bond in IM-1A and IM-4A through the transition states TS-2A and TS-9A form the product complexes, PC-1A and PC-4A, respectively. While, PC-2A and PC-3A can be formed by the breaking of C-O bond of IM-2A *via* the TSs, TS-4A1 and TS-4A2. The IM-3A also leads to the same product complexes, PC-2A and PC-4A *via* the TSs, TS-6A1 and TS-6A2. Figure 6 shows that, the IM-1A is almost barrierlessly (0.3 kcal/mol) dissociated into the product complex PC-1A, which is composed of hydrogen bonded amino formic acid (H_2_NCOOH) and $\dot{O}$ H radical (see Figure 7). The PC-1A is a highly stable product complex with an energy of ∼ -101 kcal/mol, which eventually separates into amino formic acid (NH_2_COOH) and $\dot{O}$ H radical. While, the IM-2A, IM-3A and IM-4A exhibit a barrier height of ∼13, 16 and 13 kcal/mol for the conversion into PC-2A, PC-3A and PC-4A, which are the hydrogen bond stabilized product complexes of formimidic acid (HN = C(H)-OH), formamide (H_2_N-CHO) and $\dot{O}$ _2_H radical. The formimidic acid and formamide product complexes (PC-2A, PC-3A and PC-4A) located above than that of amino formic acid (PC-1A) with an energy of −32, −36 and −49 kcal/mol, respectively (see Figure 6). Finally, these product complexes dissociate into the final products of the ^3^O_2_ + NH_2_ $\dot{C}$ HOH reaction, P-2A (HN = C(H)-OH + $\dot{O}$ _2_H), P-3A (HN = C(H)-OH + $\dot{O}$ _2_H) and P-4A (H_2_N-CHO + $\dot{O}$ _2_H) with the energies of −22, 23.5 and −38 kcal/mol, respectively.

On the other hand, the reactions proceed *via* the transition states, TS-10A, TS-11A and TS-12A directly form the product complexes. In detail, these TSs initiates the simultaneous transfer of H atom from –NH_2_ and –OH groups to the O-site of peroxy radical and the breaking of C-O bond to form the post-reactive product complexes, PC-2A, PC-3A and PC-4A, respectively. Similar to TS-3A and TS-7A, the TS-10A and TS-11A involve the transfer of inequivalent H atoms of –NH_2_ to the O-site. The energy barriers have been found to be 18, 17 and 4.3 kcal/mol for the transfer of H atom from –NH_2_ (*via* TS-10A, TS-11A) and –OH (*via* TS-12A) groups to the O-site of peroxy radical. Overall, it is evident from Figure 6 that, the reaction pathway proceeding through the transition state, TS-12A to form P-4A (NH_2_CHO) is energetically more favorable followed by that proceeding through TS-10A and TS-11A to form P-2A and P-3A (NHCHOH) by the simultaneous H transfer and CO bond breaking process rather than reaction pathways that proceeding through TS-1A, TS-3A, TS-5A, TS-7A and TS-8A.

We have compared the energetics of important reactive species of NH_2_ $\dot{C}$ H_2_OH + O_2_ reaction with that of similar species of previous studies. For example, the relative energy of IM-0A (−39 kcal/mol) is comparable to that of OH $\dot{C}$ HOH + O_2_ (−39.4 kcal/mol) (Franco et al., 2021), CH_3_ $\dot{C}$ HNH_2_+O_2_ (−36 kcal/mol) (Rissanen et al., 2014). Similarly, the barrier height values for the H transfer from–CH (40.5 kcal/mol) *via* indirect mechanism closely match with that of CH_3_ $\dot{C}$ HNH_2_+O_2_ (38 kcal/mol) (Rissanen et al., 2014), CH_2_NH_2_+O_2_ (39.5 kcal/mol) (Rissanen et al., 2014) and CH_2_OH + O_2_ (40.5 kcal/mol) reactions (Dash and Ali, 2022). On the other hand, the barrier height values for transfer of–NH hydrogen *via* an indirect mechanism (35-39 kcal/mol) are close to that of CH_2_NH_2_+O_2_ (35.8 kcal/mol) (Rissanen et al., 2014) reaction whereas *via* direct mechanism (16-18 kcal/mol) are well in agreement with the NH_2_ $\dot{C}$ HCH_2_OH + O_2_ (16.3-18.6 kcal/mol) (Silva, 2012; Xie et al., 2014) mechanistic studies. On the other hand, the direct (4.5 kcal/mol) and indirect (29 kcal/mol) –OH hydrogen transfer energy barrier values are also in similar range of OH $\dot{C}$ HOH + O_2_ (6.7 kcal/mol) (Franco et al., 2021) and CH_2_OH + O_2_ (25 kcal/mol) (Dash and Ali, 2022) reactions, respectively. These results strongly authenticate the close resemblance of energy parameters of the current study with that of previous studies.

Overall, the computed results suggest that the reaction pathways, which proceed through the TS-1A, TS-3A, TS-5A, TS-7A and TS-8A from the intermediate IM0-A exhibit high energy barriers. These energy barriers are in the range of (30-40 kcal/mol) as shown in Figure 7. Hence, these reaction pathways are excluded from the further studies of reaction kinetics and branching ratio analysis due to the high energy barriers. Relatively low energy barrier pathways which occurred *via* the transition states TS-10A, TS-11A and TS-12A were adopted for further kinetics and branching ratio calculations.

**FIGURE 8:**
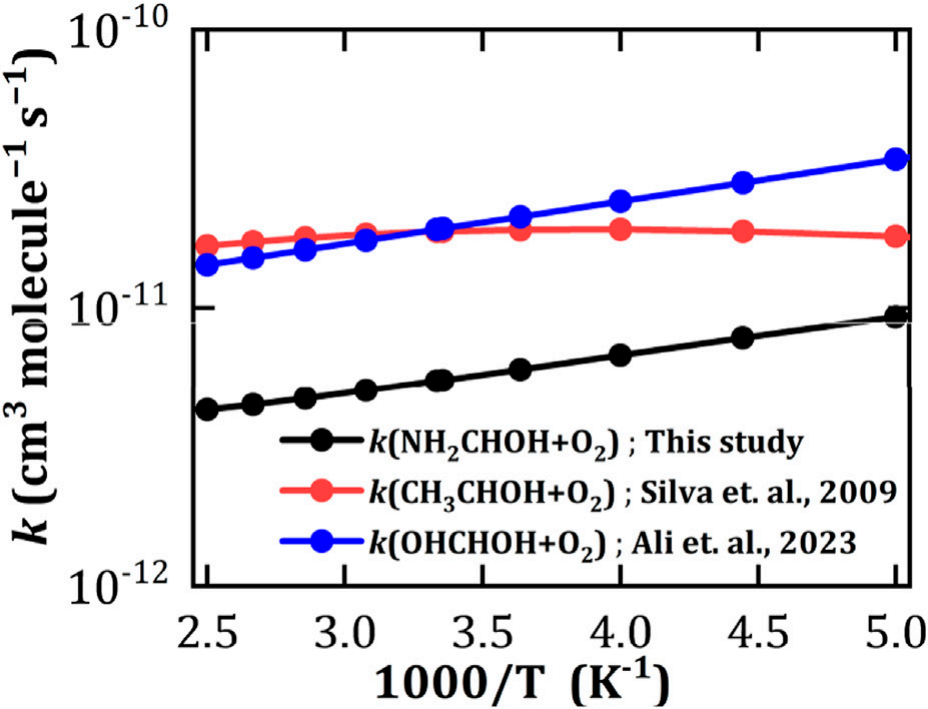
Computed rate constants for the NH_2_ $\dot{C}$ HOH + ^3^O_2_ reaction at 1 atm pressure for the formation of formamide over the temperature range 200–400 K.

The rate constants and the branching ratio values of ^3^O_2_ + NH_2_ $\dot{C}$ HOH reaction have been computed for the most favorable reaction pathways (*via* TS-10A, TS-11A and TS-12A), which form the product compounds P-2A, P-3A and P-4A. We have treated all these reaction pathways in a master equation to evaluate the temperature and pressure-dependent rate constants. The computed rate constants at the atmospheric conditions relevant to the troposphere, *i.e.*, at 1 atm pressure and 298 K for the overall reaction is 5.5 × 10^−12^ cm^3^ molecule^−1^ s^−1^. The rate constant of O_2_ + NH_2_ $\dot{C}$ HOH was compared with those of isoelectronic systems, namely, CH_3_ $\dot{C}$ HOH + O_2_ (Silva et al., 2009) and OH $\dot{C}$ HOH + O_2_ (Ali and Balaganesh, 2023) reactions. We have found that the overall rate constant of O_2_ + NH_2_ $\dot{C}$ HOH decreases with the temperature similar to that of OH $\dot{C}$ HOH + O_2_ as shown in Figure 8. The total rate constant of NH_2_ $\dot{C}$ HOH + O_2_ reaction is mainly contributed from the rate constant of P-4A reaction rather than that of P-2A and P-3A (see Supplementary Figure S6). Further, branching ratio analysis suggests that ^3^O_2_ + NH_2_ $\dot{C}$ HOH reaction predominantly leads to the formation of P-4A (NH_2_CHO) with the branching fraction of ∼99% over the temperature range 200–400 K, which is akin to the previous analysis on similar reaction system (Ali et al., 2021). The branching fraction values for the other products are negligibly small and independent of temperature and pressure.

We have also computed the ZPE corrected PES of nitrogen centered radical, namely, $\dot{N}$ HCH_2_OH + O_2_ reaction and presented in Supplementary Figure S8. The CH-direct, CH-indirect and OH-indirect reaction pathways shows the high positive energies for the TSs around 8.9 (TS5), 27.6 (TS1) and 19.5 (TS2) kcal/mol and hindered by the strong positive energy barriers, which are around 10.5, 29.2 and 22.9 kcal/mol, respectively. The large positive energies for the TSs are not encouraging and the corresponding reaction pathways will likely not be traversed in the upper atmosphere.

### 3.4 Molecular dynamics simulations

**FIGURE 9:**
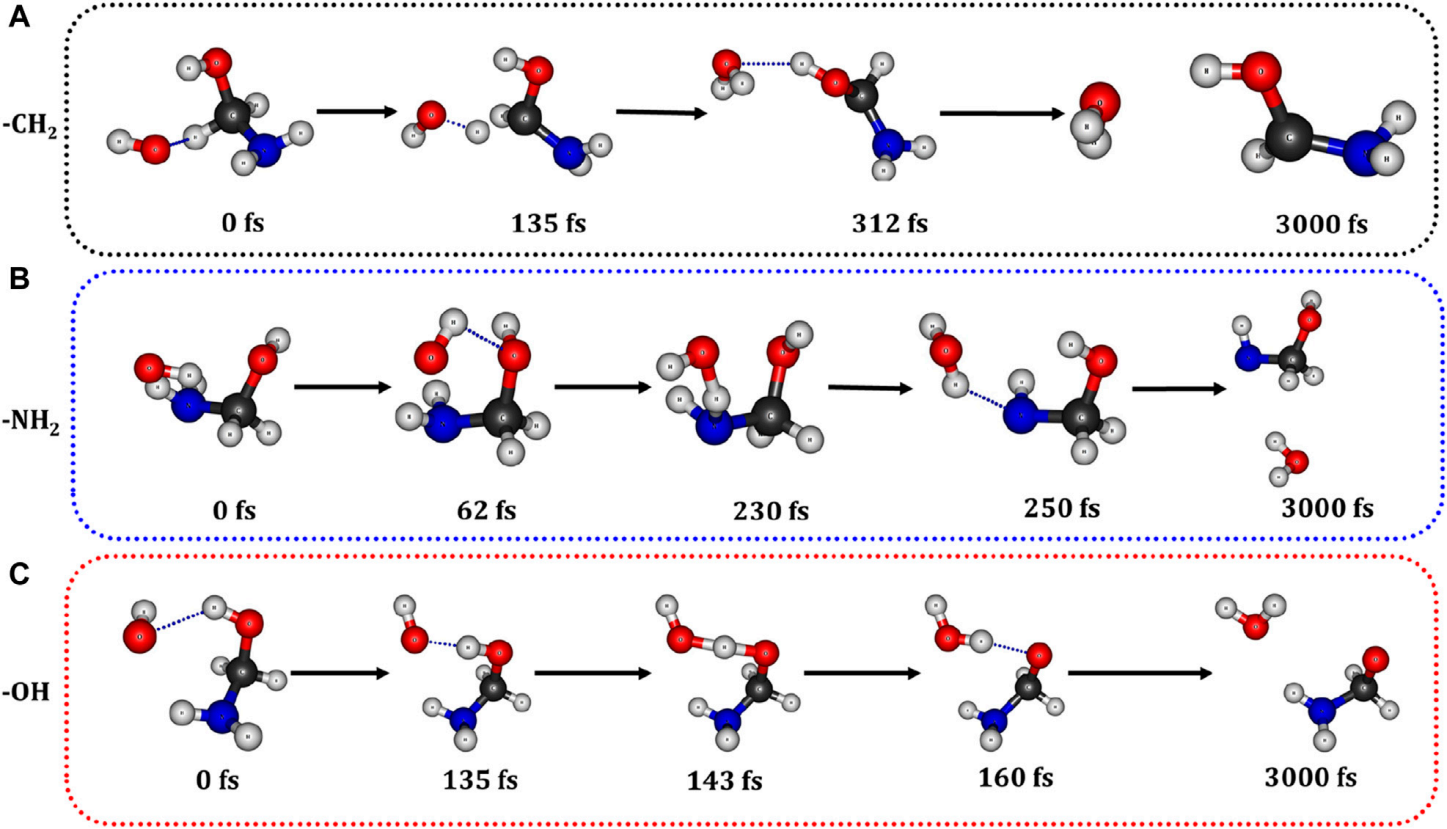
Snapshots taken at different time intervals during the BOMD simulations between $\dot{O}$ H radical and AM0. Abstraction of H from **(A)** –CH_2_, **(B)** –NH_2_ and **(C)** –OH groups by $\dot{O}$ H radical.

We have conducted Born-Oppenheimer molecular dynamics (BOMD) simulations to investigate the formation of formamide at 300 K through the reactions involving the reactive species ( $\dot{O}$ H + AM and NH_2_ $\dot{C}$ HOH radical + O_2_) using the CP2K code (VandeVondele et al., 2005). The computational methodology details are provided in the Supplementary Material. The snapshots captured at different time intervals from the trajectory of the 3ps BOMD simulation for the H abstraction from each channel of AM are shown in Figure 9. These snapshots clearly demonstrates that the abstraction of H from –CH_2_, –NH_2_ and –OH groups during the $\dot{O}$ H + AM reaction follows the similar paths as discussed in Section 3.2. For example, the initial geometry for the H abstraction by the $\dot{O}$ H radical from –NH_2_ group is depicted in Figure 9 at 0 femtoseconds (fs). During the BOMD simulation, a pre-reactive complex like geometry formed between the AM and $\dot{O}$ H radical at 62 fs. Subsequently, we have observed the transfer of H from –NH_2_ to $\dot{O}$ H radical occurring through a transition state-like geometry at 230 fs. Further, this forms a $\dot{N}$ HCH_2_OH + H_2_O complex at 250 fs. Similar mechanisms are also observed in the cases of H abstraction from –CH_2_ and –OH groups of AM as illustrated in Figure 9. Additionally, BOMD simulations have been employed to simulate the reaction between NH_2_ $\dot{C}$ HOH radical and O_2_. Snapshots captured at different time intervals during the BOMD simulations between NH_2_ $\dot{C}$ HOH and O_2_ are presented in Supplementary Figure S7, which are consistent with the earlier formamide formation reaction through TS-12A. These BOMD simulations strongly substantiate the predominant formation of formamide during the NH_2_CH_2_OH + $\dot{O}$ H/O_2_ reactions.

### 3.5 Atmospheric implications

**FIGURE 10:**
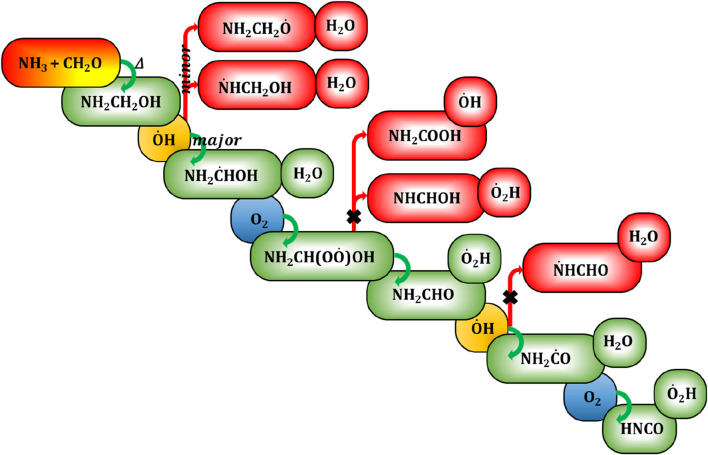
Generalized reaction mechanism for the formation of aminomethanol from ammonia and formaldehyde and its subsequent decomposition reaction pathways to potentially hazardous isocyanic acid.

Our previous investigation (Ali, 2019) has demonstrated the occurrence of a high-temperature reaction between ammonia (NH₃) and formaldehyde (H_2_CO) during biomass burning, resulting in the formation of aminomethanol (NH₂CH₂OH). This prompts a keen interest in elucidating the oxidation reaction mechanism and kinetics of aminomethanol, leading to the formation of various compounds, including potentially hazardous or carcinogenic byproducts. The possible atmospheric decomposition pathway resulting from $\dot{O}$ H radical initiated oxidation of NH₂CH₂OH is portrayed in Figure 10. We have computed the atmospheric lifetime (τ) of NH₂CH₂OH due to its interactions with $\dot{O}$ H radicals using the following the equation,

$$\tau =\frac{1}{k\times \left[\dot{O}H\right]}$$ (10)

where, *k* denotes the rate constant of NH₂CH₂OH + $\dot{O}$ H radical reaction (5.33 × 10^−11^ cm^3^ molecule^−1^ s^−1^) at 230 K, relevant to an altitude of 12 km. The averaged concentration of $\dot{O}$ H radicals in the upper troposphere is denoted by $\left[\dot{O}H\right]$ and has been taken as 1.0 × 10^6^ molecule cm^-3^ akin to that of earlier studies (Ali and Balaganesh, 2023). Computed results reveal that, the aminomethanol has a lifetime of 5 h in the presence of $\dot{O}$ H radicals and produce the NH_2_ $\dot{C}$ HOH radicals as the major product when compared to $\dot{N}$ HCH_2_OH and NH_2_CH_2_ $\dot{O}$ radicals. Under tropospheric conditions, the major radical product, *i.e.*, NH_2_ $\dot{C}$ HOH undergoes further reaction with molecular oxygen (^3^O_2_) to yield formamide (NH_2_CHO). We have employed *k* of 7.80 × 10^−12^ cm^3^ molecule^−1^ s^−1^ at 225 K to calculate the atmospheric lifetime of the NH_2_ $\dot{C}$ HOH radicals. Our results show that, the lifetime of NH_2_ $\dot{C}$ HOH is approximately 13 microseconds (μs), indicating a rapid formation of formamide under atmospheric conditions. The current study also suggests that the formations of amino formic acid (NH_2_COOH) and formimidic acid (NHCHOH) are unfavorable. It is important to mention here that the formation of formamide involves the reaction of aminomethanol with $\dot{O}$ H and O_2_. Analysis of NH_2_CH_2_OH+ $\dot{O}$ H reactions indicates that the transition states (TSs) tend to exhibit a slight positive value for the H-abstraction reactions. Consequently, these reactions pose challenges under astrochemical conditions, where temperatures typically remain below 100 K. Nevertheless, the reactions involving the carbon-centred NH_2_ $\dot{C}$ HOH radical with O_2_ may be viable in such conditions, as the corresponding aminomethanol-based peroxy radical intermediate, NH_2_CH(O $\dot{O}$ )OH required less than 5 kcal/mol for the formamide formation. Therefore, investigating the comprehensive reaction pathways for the formamide formation from aminomethanol under astrochemical conditions holds significant promise for future studies in this field.

Barnes et al., have reviewed the mechanistic details and atmospheric chemistry of amides (Barnes et al., 2010). They showed that the formamide could further react with $\dot{O}$ H radicals and produce C- and N-centered formamide radicals as shown in Figure 10. However, Zhu and co-workers have revealed that the C-centered formamide radicals have exclusively been formed and rule out the possibility for the formation of N-centered radicals using the combination of experimental and theoretical studies (Bunkan et al., 2016). They have also found that the isocyanic acid (HNCO), which is known to be a potentially hazardous compound for the human health, is only the product formed during the NH_2_CHO + OH and O_2_ reaction.

On the other hand, the branching fraction (20%) of N-centered radicals indicates the formation of $\dot{N}$ HCH_2_OH in significant quantities. It suggests that the N-centered radicals are also prone to further reactions with atmospheric gaseous compounds. However, N-centered radicals usually react slowly with the atmospheric oxygen and potentially leads to the formation of carcinogenic nitrosamines or nitramines through the bi-molecular reactions with the other trace compounds of the atmosphere (NO and NO_2_).

Overall, investigating the branching fraction and ensuing rate constants associated with the hydrogen abstraction from –CH_2_, –NH_2_, and –OH groups of aminomethanol by hydroxyl radicals offers initial insights into the predominant formation of formamide. Additionally, this study provides additional insights into the formation of N-centered radicals in significant quantities. Nevertheless, conducting further investigations on the reactions of O_2_, NO and NO_2_ with N-centered radicals would undoubtedly contribute to significantly understand their impact on the atmosphere.

## 4 Conclusion

In this study, we have comprehensively explored the mechanistic details of aminomethanol oxidation reaction initiated by atmospheric $\dot{O}$ H/O_2_ using CCSD(T)/M06-2X level of theory. The NH_2_CH_2_OH + $\dot{O}$ H reaction predominantly yields the C-centered NH_2_ $\dot{C}$ HOH radicals when compared to the N- and O-centered $\dot{N}$ HCH_2_OH and NH_2_CH $\dot{O}$ radicals. Our findings reveal that, the total rate constants ( $k_{\text{OH}}$ ) for the H transfer from aminomethanol is approximately 1.97 × 10^−11^ cm^3^ molecule^−1^ s^−1^ at 300 K. This $k_{\text{OH}}$ value closely matches with that of CH_3_NH_2_ (1.97 × 10^−11^ cm^3^ molecule^−1^ s^−1^), CH_3_NHCH_3_ (6.27 × 10^−11^ cm^3^ molecule^−1^ s^−1^), CH_3_CH_2_NH_2_ (2.50 × 10^−11^ cm^3^ molecule^−1^ s^−1^), NH_2_CH_2_CH_2_OH (7.27 × 10^−11^ cm^3^ molecule^−1^ s^−1^), indicating the accuracy of our findings. Moreover, the computed atmospheric lifetime of NH_2_ $\dot{C}$ HOH radicals is remarkably short, estimated at 13 μs, suggesting rapid reactions with atmospheric oxygen. We observed that the reaction between NH_2_ $\dot{C}$ HOH radicals and O_2_ exclusively forms formamide (NH_2_CHO) as the product, with a branching fraction of approximately 99% and a rate constant of 5.5 × 10^−12^ cm^3^ molecule^−1^ s^−1^ under the tropospheric conditions. The rate constant of NH_2_ $\dot{C}$ HOH + O_2_ reaction aligns well with that of HOCHOH + O_2_ and CH_3_CHOH + O_2_ reactions. Our Born-Oppenheimer molecular dynamics (BOMD) simulations also substantiate the formation of formamide as the prime product. Finally, we have addressed the environmental implications of exclusively formed formamide. The formamide can leads to formation of potentially hazardous compounds like HNCO on further reactions with atmospheric hydroxyl radicals. The unreacted aminomethanol $\dot{N}$ HCH_2_OH radicals may form the carcinogenic nitrosamines on reacting with trace N-oxides (*viz.*, NO and NO_2_). Consequently, it leads to increase the environmental risk factors.

## Data Availability

The original contributions presented in the study are included in the article/Supplementary Material, further inquiries can be directed to the corresponding author.

## References

[B1] Abdel-Rahman M. A. Shibl M. F. El-Nahas A. M. Abdel-Azeim S. El-demerdash S. H. Al-Hashimi N. (2021). Mechanistic insights of the degradation of an O-anisidine carcinogenic pollutant initiated by OH radical attack: theoretical investigations. New J. Chem. 45, 5907–5924. 10.1039/D0NJ06248K

[B2] Ali M. A. (2019). Theoretical study on the gas phase reaction of CH_2_O + NH_3_: the formation of CH_2_ O⋯NH_3_, NH_2_CH_2_OH, or CH_2_NH + H_2_O. Phys. Chem. Chem. Phys. 21, 19242–19251. 10.1039/C9CP02777G 31441484

[B3] Ali M. A. (2020). Computational studies on the gas phase reaction of methylenimine (CH_2_NH) with water molecules. Sci. Rep. 10, 10995. 10.1038/s41598-020-67515-3 32620911 PMC7335075

[B4] Ali M. A. Balaganesh M. (2023). Effect of formic acid on O_2_ + OH˙CHOH → HCOOH + HO_2_ reaction under tropospheric condition: kinetics of *cis* and *trans* isomers. Phys. Chem. Chem. Phys. 25, 9965–9978. 10.1039/D2CP05874J 36960665

[B5] Ali M. A. Balaganesh M. Al-Odail F. A. Lin K. C. (2021). Effect of ammonia and water molecule on OH + CH_3_OH reaction under tropospheric condition. Sci. Rep. 11, 12185. 10.1038/s41598-021-90640-6 34108500 PMC8190139

[B6] Ali M. A. Balaganesh M. Lin K. C. (2018). Catalytic effect of a single water molecule on the OH + CH_2_NH reaction. Phys. Chem. Chem. Phys. 20, 4297–4307. 10.1039/C7CP07091H 29367996

[B7] Ali M. A. Dash M. R. Al Maieli L. M. (2022). Catalytic effect of CO_2_ and H_2_O molecules on •CH_3_ + ^3^O_2_ reaction. Catalysts 12, 699. 10.3390/catal12070699

[B8] Ali M. A. Sonk J. A. Barker J. R. (2016). Predicted chemical activation rate constants for HO_2_ + CH_2_NH: the dominant role of a hydrogen-bonded pre-reactive complex. J. Phys. Chem. A 120, 7060–7070. 10.1021/acs.jpca.6b06531 27529639

[B9] Baidya B. Lily M. Chandra A. K. (2018). Theoretical insight into the kinetics of H‐abstraction reaction of CHF_2_CH_2_OH with OH radical, atmospheric lifetime and global warming potential. ChemistrySelect 3, 6136–6144. 10.1002/slct.201800491

[B10] Barker J. R. (2001). Multiple‐Well, multiple‐path unimolecular reaction systems. I. MultiWell computer program suite. Int. J. Chem. Kinet. 33, 232–245. 10.1002/kin.1017

[B11] Barker J. R. (2009). Energy transfer in master equation simulations: a new approach. Int. J. Chem. Kinet. 41, 748–763. 10.1002/kin.20447

[B12] Barker J. R. Nguyen T. L. Stanton J. F. Aieta C. Ceotto M. Gabas F. (2023) MultiWell-2023 software SuiteBarker. Ann Arbor, Michigan, USA: J. R. University of Michigan. Available at: https://multiwell.engin.umich.edu.

[B13] Barnes I. Solignac G. Mellouki A. Becker K. H. (2010). Aspects of the atmospheric chemistry of amides. ChemPhysChem 11, 3844–3857. 10.1002/cphc.201000374 20737533

[B14] Beck M. (2000). The multiconfiguration time-dependent Hartree (MCTDH) method: a highly efficient algorithm for propagating wavepackets. Phys. Rep. 324, 1–105. 10.1016/S0370-1573(99)00047-2

[B15] Ben-Nun M. Martínez T. J. (2002). *Ab initio* quantum molecular dynamics. Adv. Chem. Phys. 121, 439–512. 10.1002/0471264318.ch7

[B16] Ben-Nun M. Quenneville J. Martínez T. J. (2000). *Ab initio* multiple spawning: photochemistry from first principles quantum molecular dynamics. J. Phys. Chem. A 104, 5161–5175. 10.1021/jp994174i

[B17] Bernstein M. P. Allamandola L. J. Sandford S. A. (1997). Complex organics in laboratory simulations of interstellar/cometary ices. Adv. Space Res. 19, 991–998. 10.1016/S0273-1177(97)00340-2 11541346

[B18] Bockelée-Morvan D. Lis D. C. Wink J. E. Despois D. Crovisier J. Bachiller R. (2000). New molecules found in comet C/1995 O1 (Hale-Bopp). Investigating the link between cometary and interstellar material. Astron Astrophys. 353, 1101–1114.

[B19] Borduas N. Abbatt J. P. D. Murphy J. G. (2013). Gas phase oxidation of monoethanolamine (MEA) with OH radical and ozone: kinetics, products, and particles. Environ. Sci. Technol. 47, 6377–6383. 10.1021/es401282j 23688148

[B20] Bossa J. B. Theule P. Duvernay F. Chiavassa T. (2009). NH _2_ CH_2_ OH thermal formation in interstellar ices contribution to the 5-8 μm region toward embedded protostars. Astrophys. J. 707, 1524–1532. 10.1088/0004-637X/707/2/1524

[B21] Botta L. Saladino R. Bizzarri B. M. Cobucci-Ponzano B. Iacono R. Avino R. (2018). Formamide-based prebiotic chemistry in the phlegrean fields. Adv. Space Res. 62, 2372–2379. 10.1016/j.asr.2017.07.017

[B22] Boys S. F. Bernardi F. (1970). The calculation of small molecular interactions by the differences of separate total energies. Some procedures with reduced errors. Mol. Phys. 19, 553–566. 10.1080/00268977000101561

[B23] Bunkan A. J. C. Mikoviny T. Nielsen C. J. Wisthaler A. Zhu L. (2016). Experimental and theoretical study of the OH-initiated photo-oxidation of formamide. J. Phys. Chem. A 120, 1222–1230. 10.1021/acs.jpca.6b00032 26859252

[B24] Dai N. Shah A. D. Hu L. Plewa M. J. McKague B. Mitch W. A. (2012). Measurement of nitrosamine and nitramine formation from NO _x_ reactions with amines during amine-based carbon dioxide capture for postcombustion carbon sequestration. Environ. Sci. Technol. 46, 9793–9801. 10.1021/es301867b 22831707

[B25] Dash M. R. Ali M. A. (2022). Effect of a single water molecule on ˙CH _2_ OH + ^3^ O _2_ reaction under atmospheric and combustion conditions. Phys. Chem. Chem. Phys. 24, 1510–1519. 10.1039/D1CP03911C 34935796

[B26] Dash M. R. Ali M. A. (2023). Can a single ammonia and water molecule enhance the formation of methanimine under tropospheric conditions? kinetics of •CH_2_NH_2_ + O_2_ (+NH_3_/H_2_O). Front. Chem. 11, 1243235. 10.3389/fchem.2023.1243235 37810581 PMC10552757

[B27] Deschamps G. (1931). Preparation of formamide and its conversion into hydrocyanic acid. Chimie Industrie, 589–597.

[B28] Evans M. G. Polanyi M. (1938). Inertia and driving force of chemical reactions. Trans. Faraday Soc. 34, 11. 10.1039/tf9383400011

[B29] Fabiano E. Keal T. W. Thiel W. (2008). Implementation of surface hopping molecular dynamics using semiempirical methods. Chem. Phys. 349, 334–347. 10.1016/j.chemphys.2008.01.044

[B30] Feldmann M. T. Widicus S. L. Blake G. A. Kent D. R. Goddard W. A. (2005). Aminomethanol water elimination: theoretical examination. J. Chem. Phys. 123, 034304. 10.1063/1.1935510 16080734

[B31] Ferus M. Knížek A. Cassone G. Rimmer P. B. Changela H. Chatzitheodoridis E. (2023). Simulating asteroid impacts and meteor events by high-power lasers: from the laboratory to spaceborne missions. Front. Astron Space Sci. 10. 10.3389/fspas.2023.1186172

[B32] Ferus M. Nesvorný D. Šponer J. Kubelík P. Michalčíková R. Shestivská V. (2015). High-energy chemistry of formamide: a unified mechanism of nucleobase formation. Proc. Natl. Acad. Sci. 112, 657–662. 10.1073/pnas.1412072111 25489115 PMC4311869

[B33] Firaha D. S. Döntgen M. Berkels B. Leonhard K. (2018). Pressure-dependent rate constant predictions utilizing the inverse Laplace transform: a victim of deficient input data. ACS Omega 3, 8212–8219. 10.1021/acsomega.8b00311 31458958 PMC6644344

[B34] Franco B. Blumenstock T. Cho C. Clarisse L. Clerbaux C. Coheur P.-F. (2021). Ubiquitous atmospheric production of organic acids mediated by cloud droplets. Nature 593, 233–237. 10.1038/s41586-021-03462-x 33981052 PMC8116209

[B35] Frisch M. J. Pople J. A. Binkley J. S. (1984). Self-consistent molecular orbital methods 25. Supplementary functions for Gaussian basis sets. J. Chem. Phys. 80, 3265–3269. 10.1063/1.447079

[B36] Frisch M. J. Trucks G. W. Schlegel H. B. Scuseria G. E. Robb M. A. Cheeseman J. R. (2019) Gaussian 16, revision C.01. Wallingford CT: Gaussian, Inc.

[B37] Ge X. Wexler A. S. Clegg S. L. (2011a). Atmospheric amines – Part I. A review. Atmos. Environ. 45, 524–546. 10.1016/j.atmosenv.2010.10.012

[B38] Ge X. Wexler A. S. Clegg S. L. (2011b). Atmospheric amines – Part II. Thermodynamic properties and gas/particle partitioning. Atmos. Environ. 45, 561–577. 10.1016/j.atmosenv.2010.10.013

[B39] Gerakines P. A. Moore M. H. Hudson R. L. (2004). Ultraviolet photolysis and proton irradiation of astrophysical ice analogs containing hydrogen cyanide. Icarus 170, 202–213. 10.1016/j.icarus.2004.02.005

[B40] Goldsmith C. F. Green W. H. Klippenstein S. J. (2012). Role of O _2_ + QOOH in low-temperature ignition of propane. 1. Temperature and pressure dependent rate coefficients. J. Phys. Chem. A 116, 3325–3346. 10.1021/jp210722w 22250995

[B41] González D. Lema-Saavedra A. Espinosa S. Martínez-Núñez E. Fernández-Ramos A. Canosa A. (2022). Reaction of OH radicals with CH _3_ NH _2_ in the gas phase: experimental (11.7–177.5 K) and computed rate coefficients (10–1000 K). Phys. Chem. Chem. Phys. 24, 23593–23601. 10.1039/D2CP03414J 36134502

[B42] Grimme S. Antony J. Ehrlich S. Krieg H. (2010). A consistent and accurate *ab initio* parametrization of density functional dispersion correction (DFT-D) for the 94 elements H-Pu. J. Chem. Phys. 132, 154104. 10.1063/1.3382344 20423165

[B43] Gröger H. (2003). Catalytic enantioselective strecker reactions and analogous syntheses. Chem. Rev. 103, 2795–2828. 10.1021/cr020038p 12914481

[B44] Hammes-Schiffer S. Tully J. C. (1994). Proton transfer in solution: molecular dynamics with quantum transitions. J. Chem. Phys. 101, 4657–4667. 10.1063/1.467455

[B45] Hays B. M. Weaver W. Susanna L. (2013). Theoretical examination of O(^1^ D) insertion reactions to form methanediol, methoxymethanol, and aminomethanol. J. Phys. Chem. A 117, 7142–7148. 10.1021/jp400753r 23646865

[B46] Kapteina S. Slowik K. Verevkin S. P. Heintz A. (2005). Vapor pressures and vaporization enthalpies of a series of ethanolamines. J. Chem. Eng. Data 50, 398–402. 10.1021/je049761y

[B47] Karl M. Dye C. Schmidbauer N. Wisthaler A. Mikoviny T. D’Anna B. (2012). Study of OH-initiated degradation of 2-aminoethanol. Atmos. Chem. Phys. 12, 1881–1901. 10.5194/acp-12-1881-2012

[B48] Koike T. Kaneko T. Kobayashi K. Miyakawa S. Takano Y. (2003). Formation of organic compounds from simulated Titan atmosphere: perspectives of the Cassini mission. Biol. Sci. Space 17, 188–189. 14676367

[B49] Lee T. J. Taylor P. R. (1989). A diagnostic for determining the quality of single-reference electron correlation methods. Int. J. Quantum Chem. 36, 199–207. 10.1002/qua.560360824

[B50] Lis D. C. Mehringer D. M. Benford D. Gardner M. Phillips T. G. Bockelée-Morvan D. (1997). New molecular species in comet C/1995 O1 (Hale-Bopp) observed with the caltech ssubmillimeter observatory. Earth Moon Planets 78, 13–20. 10.1023/A:1006281802554

[B51] Manthe U. Meyer H.-D. Cederbaum L. S. (1992). Wave-packet dynamics within the multiconfiguration Hartree framework: general aspects and application to NOCl. J. Chem. Phys. 97, 3199–3213. 10.1063/1.463007

[B52] Mazarei E. Barker J. R. (2022). CH2 + O2: reaction mechanism, biradical and zwitterionic character, and formation of CH2OO, the simplest Criegee intermediate. Phys. Chem. Chem. Phys. 24, 914–927. 10.1039/D1CP04372B 34913447

[B53] Meyer H. (2012). Studying molecular quantum dynamics with the multiconfiguration time‐dependent Hartree method. WIREs Comp. Mol. Sci. 2, 351–374. 10.1002/wcms.87

[B54] Nájera C. Sansano J. M. (2007). Catalytic asymmetric synthesis of α-amino acids. Chem. Rev. 107, 4584–4671. 10.1021/cr050580o 17915933

[B55] Navarro-González R. Raulin F. (2004). Steps toward the origin(s) of life: endogenous sources and chemistry. Adv. Space Res. 33, 79–80. 10.1016/j.asr.2003.11.002

[B56] Nguyen T. L. Ruscic B. Stanton J. F. (2019). A master equation simulation for the •OH + CH_3_OH reaction. J. Chem. Phys. 150, 084105. 10.1063/1.5081827 30823757

[B57] Nielsen A. T. Moore D. W. Ogan M. D. Atkins R. L. (1979). Structure and chemistry of the aldehyde ammonias. 3. Formaldehyde-ammonia reaction. 1,3,5-Hexahydrotriazine. J. Org. Chem. 44, 1678–1684. 10.1021/jo01324a021

[B58] Onel L. Blitz M. A. Seakins P. W. (2012). Direct determination of the rate coefficient for the reaction of OH radicals with monoethanol amine (MEA) from 296 to 510 K. J. Phys. Chem. Lett. 3, 853–856. 10.1021/jz300200c 26286410

[B59] Onel L. Thonger L. Blitz M. A. Seakins P. W. Bunkan A. J. C. Solimannejad M. (2013). Gas-phase reactions of OH with methyl amines in the presence or absence of molecular oxygen. An experimental and theoretical study. J. Phys. Chem. A 117, 10736–10745. 10.1021/jp406522z 24059646

[B60] Pastorek A. Hrnčířová J. Jankovič L. Nejdl L. Civiš S. Ivanek O. (2019). Prebiotic synthesis at impact craters: the role of Fe-clays and iron meteorites. Chem. Comm. 55, 10563–10566. 10.1039/C9CC04627E 31417990

[B61] Priya A. M. Lakshmipathi S. (2017). DFT study on abstraction reaction mechanism of oh radical with 2-methoxyphenol. J. Phys. Org. Chem. 30, e3713. 10.1002/poc.3713

[B62] Raghavachari K. Trucks G. W. Pople J. A. Head-Gordon M. (1989). A fifth-order perturbation comparison of electron correlation theories. Chem. Phys. Lett. 157, 479–483. 10.1016/S0009-2614(89)87395-6

[B63] Rahbar A. Zahedi E. Aghaie H. Giahi M. Zare K. (2021). DFT insight into the kinetics and mechanism of the OH ^.^ ‐initiated atmospheric oxidation of catechol: OH ^.^ Addition and hydrogen abstraction pathways. Chem. Sel. 6, 3875–3883. 10.1002/slct.202100524

[B64] Rissanen M. P. Eskola A. J. Nguyen T. L. Barker J. R. Liu J. Liu J. (2014). CH _2_ NH _2_ + O _2_ and CH _3_ CHNH _2_ + O _2_ reaction kinetics: photoionization mass spectrometry experiments and master equation calculations. J. Phys. Chem. A 118, 2176–2186. 10.1021/jp411238e 24592923

[B65] Robertson S. H. Pilling M. J. Baulch D. L. Green N. J. B. (1995). Fitting of pressure-dependent kinetic rate data by master equation/inverse Laplace transform analysis. J. Phys. Chem. 99, 13452–13460. 10.1021/j100036a020

[B66] Rubin R. H. Swenson Jr G. W. Benson R. C. Tigelaar H. L. Flygare W. H. (1971). Microwave detection of interstellar formamide. Astrophysical J. 169, L39. 10.1086/180810

[B67] Saitta A. M. Saija F. (2014). Miller experiments in atomistic computer simulations. Proc. Natl. Acad. Sci. 111, 13768–13773. 10.1073/pnas.1402894111 25201948 PMC4183268

[B68] Saladino R. Botta G. Pino S. Costanzo G. Di Mauro E. (2012a). Genetics first or metabolism first? The formamide clue. Chem. Soc. Rev. 41, 5526. 10.1039/c2cs35066a 22684046

[B69] Saladino R. Crestini C. Ciciriello F. Costanzo G. Di Mauro E. (2007). Formamide chemistry and the origin of informational polymers. Chem. Biodivers. 4, 694–720. 10.1002/cbdv.200790059 17443884

[B70] Saladino R. Crestini C. Costanzo G. DiMauro E. (2005). “On the prebiotic synthesis of nucleobases, nucleotides, oligonucleotides, pre-RNA and pre-DNA molecules,” in Prebiotic chemistry. Editor Walde P. (Berlin/Heidelberg: Springer-Verlag), 29–68. 10.1007/b136152

[B71] Saladino R. Crestini C. Pino S. Costanzo G. Di Mauro E. (2012b). Formamide and the origin of life. Phys. Life Rev. 9, 84–104. 10.1016/j.plrev.2011.12.002 22196896

[B72] Schade G. W. Crutzen P. J. (1995). Emission of aliphatic amines from animal husbandry and their reactions: potential source of N2O and HCN. J. Atmos. Chem. 22, 319–346. 10.1007/BF00696641

[B73] Schutte W. A. Allamandola L. J. Sandford S. A. (1993). An experimental study of the organic molecules produced in cometary and interstellar ice analogs by thermal formaldehyde reactions. Icarus 104, 118–137. 10.1006/icar.1993.1087 11540089

[B74] Schutte W. A. Boogert A. C. A. Tielens A. Whittet D. C. B. Gerakines P. A. Chiar J. E. (1999). Weak ice absorption features at 7.24 and 7.41 MU M in the spectrum of the obscured young stellar object W 33A. Astron Astrophys. 343, 966–976.

[B75] Silva G. D. (2012). Atmospheric chemistry of 2-aminoethanol (MEA): reaction of the NH_2_ •CHCH_2_ OH radical with O_2_ . J. Phys. Chem. A 116, 10980–10986. 10.1021/jp307726w 22994372

[B76] Silva G. da Bozzelli J. W. Liang L. Farrell J. T. (2009). Ethanol oxidation: kinetics of the α-hydroxyethyl radical + O2 reaction. J. Phys. Chem. A 113, 8923–8933. 10.1021/jp903210a 19594149

[B77] Simon S. Duran M. Dannenberg J. J. (1996). How does basis set superposition error change the potential surfaces for hydrogen-bonded dimers? J. Chem. Phys. 105, 11024–11031. 10.1063/1.472902

[B78] Singh S. K. Zhu C. La Jeunesse J. Fortenberry R. C. Kaiser R. I. (2022). Experimental identification of aminomethanol (NH2CH2OH)—the key intermediate in the Strecker Synthesis. Nat. Commun. 13, 375. 10.1038/s41467-022-27963-z 35046418 PMC8770675

[B79] Solomon P. M. (1973). Interstellar molecules. Phys. Today 26, 32–40. 10.1063/1.3127983

[B80] Strecker A. (1850). Ueber die künstliche Bildung der Milchsäure und einen neuen, dem Glycocoll homologen Körper. Justus Liebigs Ann. Chem. 75, 27–45. 10.1002/jlac.18500750103

[B81] Takano Y. Tsuboi T. Kaneko T. Kobayashi K. Marumo K. (2004). Pyrolysis of high-molecular-weight complex organics synthesized from a simulated interstellar gas mixture irradiated with 3 MeV proton beam. Bull. Chem. Soc. Jpn. 77, 779–783. 10.1246/bcsj.77.779

[B82] Tian W. Wang W. Zhang Y. Wang W. (2009). Direct dynamics study on the mechanism and the kinetics of the reaction of CH_3_NH_2_ with OH. Int. J. Quantum Chem. 109, 1566–1575. 10.1002/qua.22000

[B83] Tully J. C. Preston R. K. (1971). Trajectory surface hopping approach to nonadiabatic molecular collisions: the reaction of H+ with D_2_ . J. Chem. Phys. 55, 562–572. 10.1063/1.1675788

[B84] VandeVondele J. Krack M. Mohamed F. Parrinello M. Chassaing T. Hutter J. (2005). Quickstep: fast and accurate density functional calculations using a mixed Gaussian and plane waves approach. Comput. Phys. Commun. 167, 103–128. 10.1016/j.cpc.2004.12.014

[B85] Wang J. Liu X. Feng X. (2011). Asymmetric strecker reactions. Chem. Rev. 111, 6947–6983. 10.1021/cr200057t 21851054

[B86] Woon D. E. (2002). *Ab initio* quantum chemical studies of reactions in astrophysical ices. 4. Reactions in ices involving HCOOH, CH_2_NH, HCN, HNC, NH_3_, and H_2_O. Int. J. Quantum Chem. 88, 226–235. 10.1002/qua.10082

[B87] Xie H.-B. Li C. He N. Wang C. Zhang S. Chen J. (2014). Atmospheric chemical reactions of monoethanolamine initiated by OH radical: mechanistic and kinetic study. Environ. Sci. Technol. 48, 1700–1706. 10.1021/es405110t 24438015

[B88] Xu L. Tsona N. T. Tang S. Li J. Du L. (2019). Role of (H _2_ O) _n_ (*n* = 1–2) in the gas-phase reaction of ethanol with hydroxyl radical: mechanism, kinetics, and products. ACS Omega 4, 5805–5817. 10.1021/acsomega.9b00145 31459732 PMC6648320

[B89] Yamada H. (2021). Amine-based capture of CO2 for utilization and storage. Polym. J. 53, 93–102. 10.1038/s41428-020-00400-y

[B90] Yang S. Martínez T. J. (2011). “ *Ab initio* multiple spawning: first principles dynamics around conical intersections,” in Conical intersections (Singapore: World Scientific), 347–374. 10.1142/9789814313452_0009

[B91] Zhao Y. Truhlar D. G. (2008). The M06 suite of density functionals for main group thermochemistry, thermochemical kinetics, noncovalent interactions, excited states, and transition elements: two new functionals and systematic testing of four M06-class functionals and 12 other functionals. Theor. Chem. Acc. 120, 215–241. 10.1007/s00214-007-0310-x

