# Supplementary material for: Unveiling the chemical kinetics of aminomethanol (NH2CH2OH): insights into O. H and O2 photo-oxidation reactions and formamide dominance
Source: Front Chem. 2024 May 30;12:1407355. doi: 10.3389/fchem.2024.1407355 (PMC11169873; doi:10.3389/fchem.2024.1407355)
Supplement: Supplementary file 1 [file DataSheet1.docx]

**Supporting Information for**

**Unveiling the Chemical Kinetics of Aminomethanol (NH_2_CH_2_OH): Insights into** $\dot{\mathbf{O}}$**H and O_2_ Photo-Oxidation Reactions and Formamide Dominance**

Naga Venkateswara Rao Nulakani,^a,b^ and Mohamad Akbar Ali^a,b*^

^a^Department of Chemistry, Khalifa University of Science and Technology, P.O. Box 127788, Abu Dhabi, UAE.

^b^Center for the Catalyst and Separations, Khalifa University of Science and Technology, P.O. Box 127788, Abu Dhabi, UAE.

***Corresponding Author**:

**E-mail**: [akbar.mohamad@ku.ac.ae](mailto:akbar.mohamad@ku.ac.ae)

| **S. No** | **Table of Contents** | | **Page** |
| --- | --- | --- | --- |
| 1 |  | 1. Unimolecular and bi-molecular rate constants | 3 |
| 2 |  | 1. RRKM/ME microscopic rate-constant | 4 |
| 3 |  | 1. Born-Oppenheimer molecular dynamics simulations | 4 |
| 4 | **Figure S1.** | Zero-point energy corrected potential energy surface of AM1 + $\dot{O}$H radical. | 5 |
| 5 | **Figure S2.** | Equilibrium geometries of aminomethanol (AM1), PRC, TSs, PORCs and the radicals of $\dot{O}$H initiated AM1 oxidation reaction. All the values in the parenthesis are in kcal/mol. | 5 |
| 6 | **Figure S3.** | Zero-point energy corrected potential energy surface of AM2 + $\dot{O}$H radical. | 6 |
| 7 | **Figure S4.** | Equilibrium geometries of aminomethanol (AM1), PRC, TSs, PORCs and the radicals of $\dot{O}$H initiated AM2 oxidation reaction. All the values in the parenthesis are in kcal/mol. | 6 |
| 8 | **Figure S5.** | Different rotational conformations of aminomethanol bases peroxyradical intermediate. The values in the parenthesis indicate the relative energies in kcal/mol. | 7 |
| 9 | **Figure S6**. | Computed rate constants for the NH_2_$\dot{C}$HOH + ^3^O_2_ reaction at 1 atm pressure for the formation of formamide (*k*_P-4A_) and formimidic acid (*k*_P-2A and_ *k*_P-3A_) over the temperature range 200–400 K. | 7 |
| 10 | **Figure S7**. | Snapshots taken at different time intervals during the BOMD simulations between the NH_2_$\dot{C}$HOH and O_2_. | 7 |
| 11 | **Figure S8.** | The zero-point energy (ZPE) corrected potential energy surface of AM radical ($\dot{N}$HCH_2_OH) + ^3^O_2_ radical. All the energy values are in kcal/mol. | 8 |
| 12 | **Table S1**. | Calculated energy parameters of PRC, TS, PORC, products (PROD) and the activation energy (*E*_a_) for hydrogen abstraction reaction from different H-bearing groups of three rotational conformers of aminomethanol. | 9 |
|  |  |  |  |
|  |  |  |  |
| 17 | **Table S2**. | Calculated temperature dependent rate constants for the $\dot{O}$H initiated oxidation reaction of aminomethanol using the rotational conformation, AM0. | 10 |
| 18 | **Table S3**. | Calculated temperature dependent rate constants for the $\dot{O}$H initiated oxidation reaction of aminomethanol using the rotational conformation, AM1. | 11 |
| 15 | **Table S4**. | Calculated temperature dependent rate constants for the $\dot{O}$H initiated oxidation reaction of aminomethanol using the rotational conformation, AM2. | 12 |
| 16 | **Table S5.** | Calculated temperature dependent total rate constants for AM0+$\dot{O}$H, AM1+$\dot{O}$H and AM2+$\dot{O}$H radical reactions with and without BSSE corrections. | 13 |
| 13 | **Table S6.** | Cartesian coordinates of the equilibrium geometries of aminomethanol (AM1, AM2 and AM3), PRCs, TSs, PORCs, carbon, nitrogen and oxygen-centred radicals of $\dot{O}$H + aminomethanol oxidation reaction. | 14 |
| 14 | **Table S7**. | Vibrational frequencies and rotational constants of aminomethanol (AM0, AM1, AM2) + $\dot{O}$H radical oxidation reaction. | 18 |
| 18 | **Table S8**. | Cartesian coordinates of the equilibrium geometries of NH_2_$\dot{C}$HOH, O_2_, intermediates (IMs), transition states (TSs), product complexes (PCs) and the products (Ps) of O_2_ + carbon cantered NH_2_$\dot{C}$HOH aminomethanol radical reaction. | 22 |
| 19 | **Table S9**. | Vibrational frequencies and rotational constants of of O_2_ + carbon cantered NH_2_$\dot{C}$HOH aminomethanol radical reaction. | 25 |
| 20 | **Table S10**. | Bimolecular (*k*_e_) and unimolecular (*k*_uni_) rate constants for AM0 + $\dot{O}$H radical reaction. | 28 |
| 21 | **Table S11**. | Bimolecular (*k*_e_) and unimolecular (*k*_uni_) rate constants for AM0 + $\dot{O}$H radical reaction. | 29 |
| 22 | **Table S12**. | Equilibrium Constant (*K*_e_) and unimolecular (*k*_uni_) rate constants for AM0 + $\dot{O}$H radical reaction. | 30 |
| 23 |  | References | 31 |

**1. Unimolecular and bi-molecular rate constants**

The kinetic rate constants for these bimolecular (*k*_b,_ in cm^3^ molecule^-1^ s^-1^) and unimolecular (*k*_uni,_ s^-1^) reactions in the high-pressure limit defined by transition state theory.

**(a) bi-molecular rate constant (*k*_e_)**

$k_{e}=\Gamma(T)\times\frac{\sigma k_{B}T}{h}\times\frac{Q_{IM}}{Q_{R}}\times\frac{V_{m}\left( T \right)}{N_{AV}}\times exp(-\frac{{\Delta E}_{0}}{RT})$

where,

| $\Gamma(T)$ | = | quantum mechanical tunnelling correction |
| --- | --- | --- |
| σ | = | tunnelling factor |
| *k_b_* | = | Boltzmann’s constant |
| T | = | temperature |
| *h* | = | Planck's constant |
| *Q_IM_* | = | total molecular partition functions for the pre-reactive molecular complex (IM) |
| *Q_R_* | = | total molecular partition functions for the isolated reactants |
| *V_m_(T)* | = | molar volume of an ideal gas |
| *N_AV_* | = | Avogadro number |
| *R* | = | ideal gas constant |
| ${\Delta E}_{0}$ | = | *E*_R_-*E*_IM_ (Energy difference of pre-reactive molecular complex (IM) and its reactants) |

**(b) uni-molecular rate constant (*k*_uni_)**

$$k_{uni}=\Gamma(T)\times\frac{\sigma k_{B}T}{h}\times\frac{Q_{TS}}{Q_{IM}}\times exp(-\frac{{\Delta E}_{0}}{RT})$$

where,

| $\Gamma(T)$ | = | quantum mechanical tunnelling correction |
| --- | --- | --- |
| σ | = | tunnelling factor |
| *k_b_* | = | Boltzmann’s constant |
| T | = | temperature |
| *h* | = | Planck's constant |
| *Q_TS_* | = | total molecular partition functions for transition state (TS) |
| *Q_IM_* | = | total molecular partition functions for the pre-reactive molecular complex (IM) |
| *V_m_(T)* | = | molar volume of an ideal gas |
| *N_AV_* | = | Avogadro number |
| *R* | = | ideal gas constant |
| ${\Delta E}_{0}$ | = | *E*_IM_-*E*_TS_ (Energy difference of pre-reactive molecular complex (IM) and its transition state) |

**2. RRKM/ME microscopic rate-constant**

The RRKM/ME microscopic rate-constant *k*(E) is defined as follows,

$$k\left( E \right)=\left[ \frac{m^{\neq}}{m}\frac{\sigma_{ext}}{\sigma_{ext}^{\neq}} \right]\frac{g_{e}^{\neq}}{g_{e}}\frac{1}{h}\frac{G^{\neq}\left( E-E_{0} \right)}{\rho\left( E \right)}$$

where**,**

| m^≠^ | = | number of optical isomers of transition state |
| --- | --- | --- |
| m | = | number of optical isomers of reactant |
| $\sigma_{ext}^{\neq}$ | = | external rotation symmetry numbers of the transition state |
| σ*_ext_* | = | external rotation symmetry numbers of reactant |
| $g_{e}^{\neq}$ | = | electronic state degeneracy for the transition state |
| $g_{e}$ | = | electronic state degeneracy for the reactant |
| *h* | = | Planck's constant |
| *G^≠^(E-E_0_)* | = | sum of states of the transition state |
| *E*_0_ | = | reaction critical energy |
| *ρ*(*E)* | = | density of states of the reactants |

**3. Born-Oppenheimer molecular dynamics simulations**

The Born-Oppenheimer molecular dynamics (BOMD) simulation utilizing the DFT method was conducted using the CP2K 6.1.0 program (VandeVondele et al., 2005). We employed the Becke-Lee-Yang-Parr exchange-correlation functional (BLYP-D3) (Becke, 1988; Lee et al., 1988) with dispersion correction to address weak interactions in this study (Grimme et al., 2010). Additionally, the resolution of valence and core electrons was achieved using the double-ζ valence polarization (DZVP) Gaussian basis set(VandeVondele and Hutter, 2007) and Goedecker-Teter-Hutter (GTH) norm-conserved pseudopotentials (Goedecker et al., 1996; Hartwigsen et al., 1998), respectively. The energy cutoff for the plane wave basis set is 280 Ry, while it's 40 Ry for the Gaussian basis set. Constant volume and 300K temperature NVT ensemble were utilized in BOMD simulations, with a simulation time step of 1.0 fs. Periodic supercells with side lengths of 15 Å were employed for the gas-phase reaction.

**
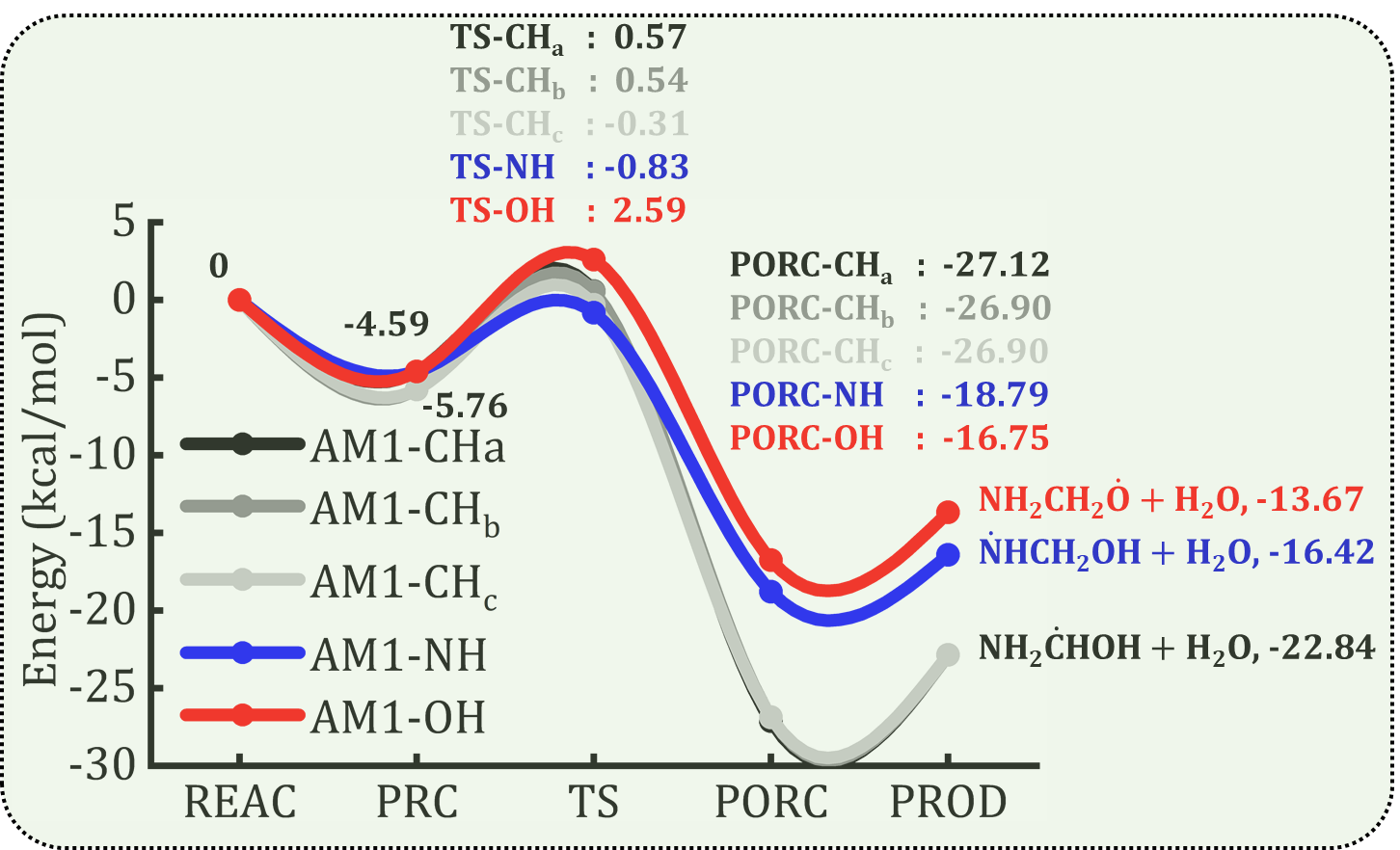
**

**Figure S1.** Zero-point energy corrected potential energy surface of AM1 + $\dot{O}$H radical.


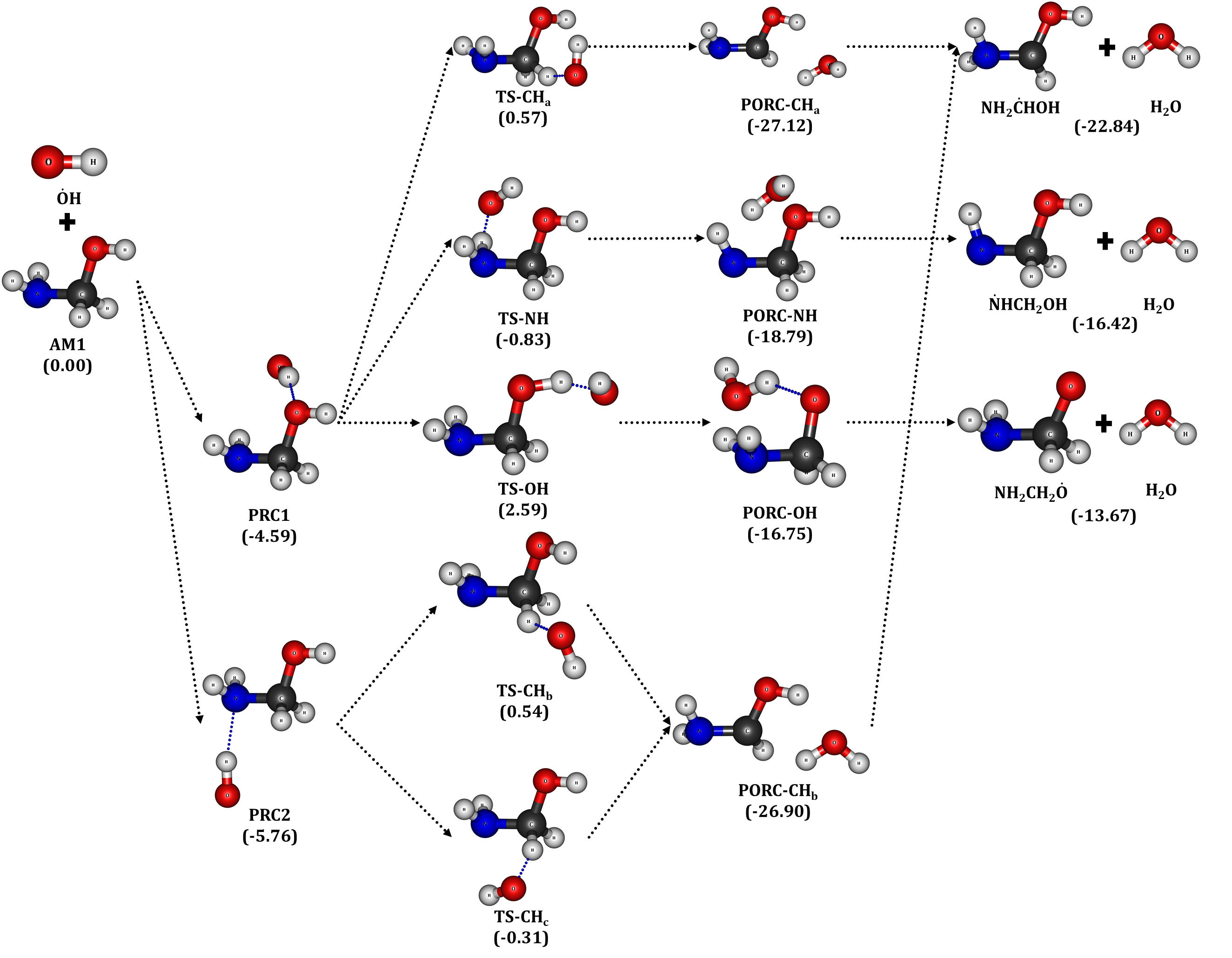


**Figure S2.** Equilibrium geometries of aminomethanol (AM1), PRC, TSs, PORCs and the radicals of $\dot{O}$H initiated AM1 oxidation reaction. All the values in the parenthesis are in kcal/mol.


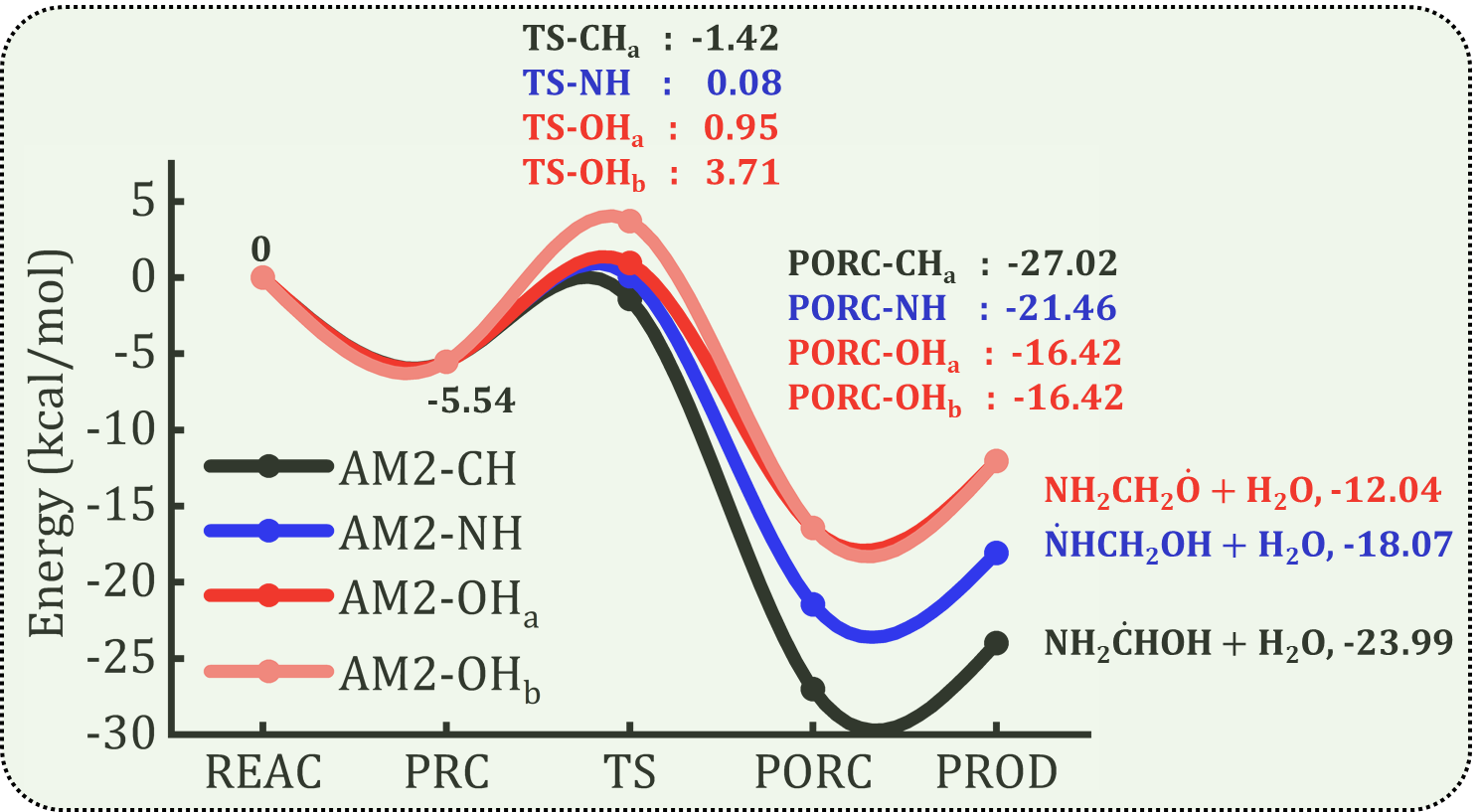


**Figure S3.** Zero-point energy corrected potential energy surface of AM2 + $\dot{O}$H radical.

**
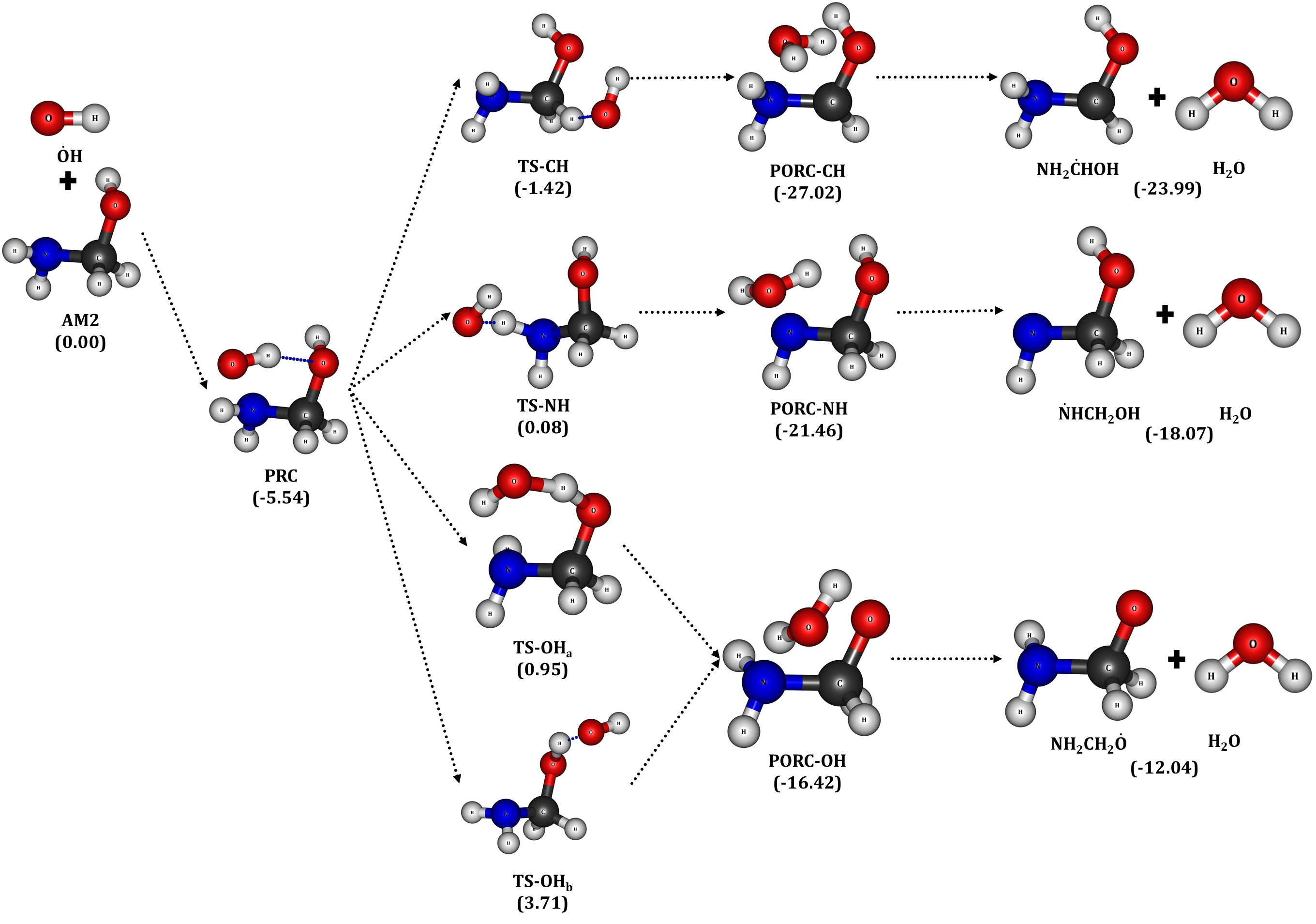
**

**Figure S4.** Equilibrium geometries of aminomethanol (AM1), PRC, TSs, PORCs and the radicals of $\dot{O}$H initiated AM2 oxidation reaction. All the values in the parenthesis are in kcal/mol.

**
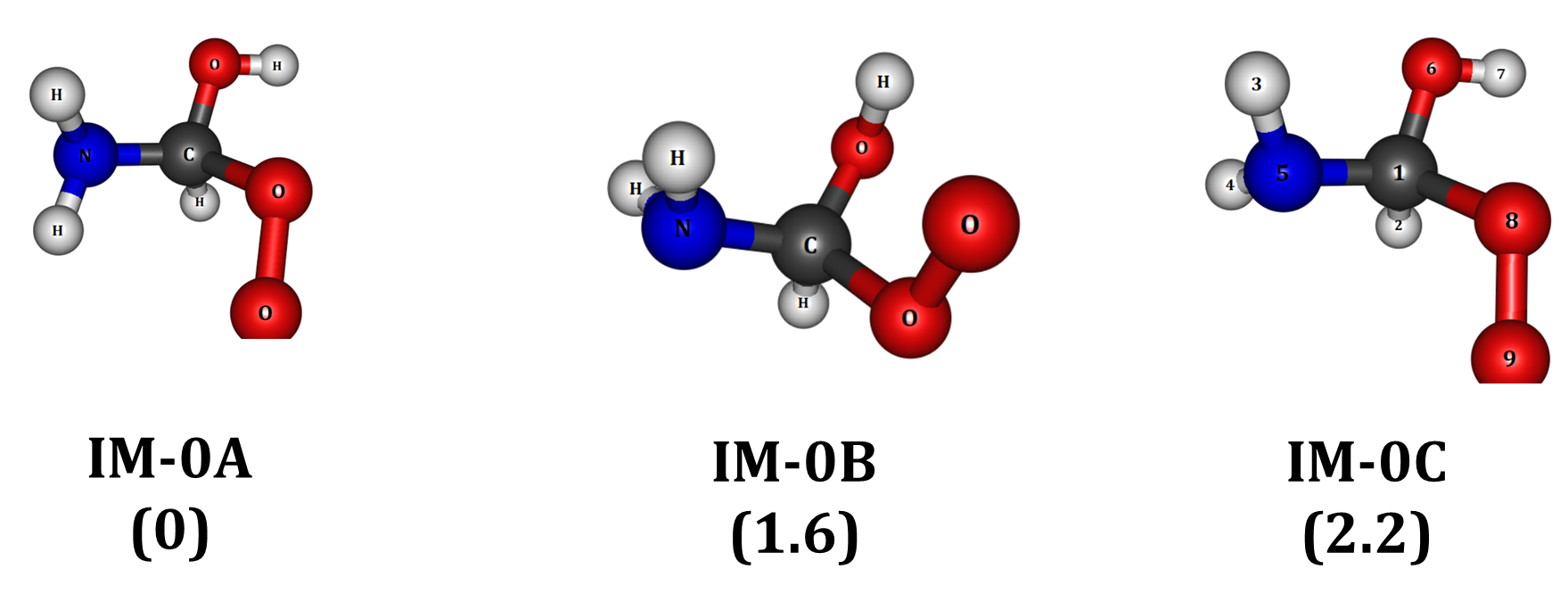
**

**Figure S5.** Different rotational conformations of aminomethanol bases peroxyradical intermediate. The values in the parenthesis indicate the relative energies in kcal/mol.


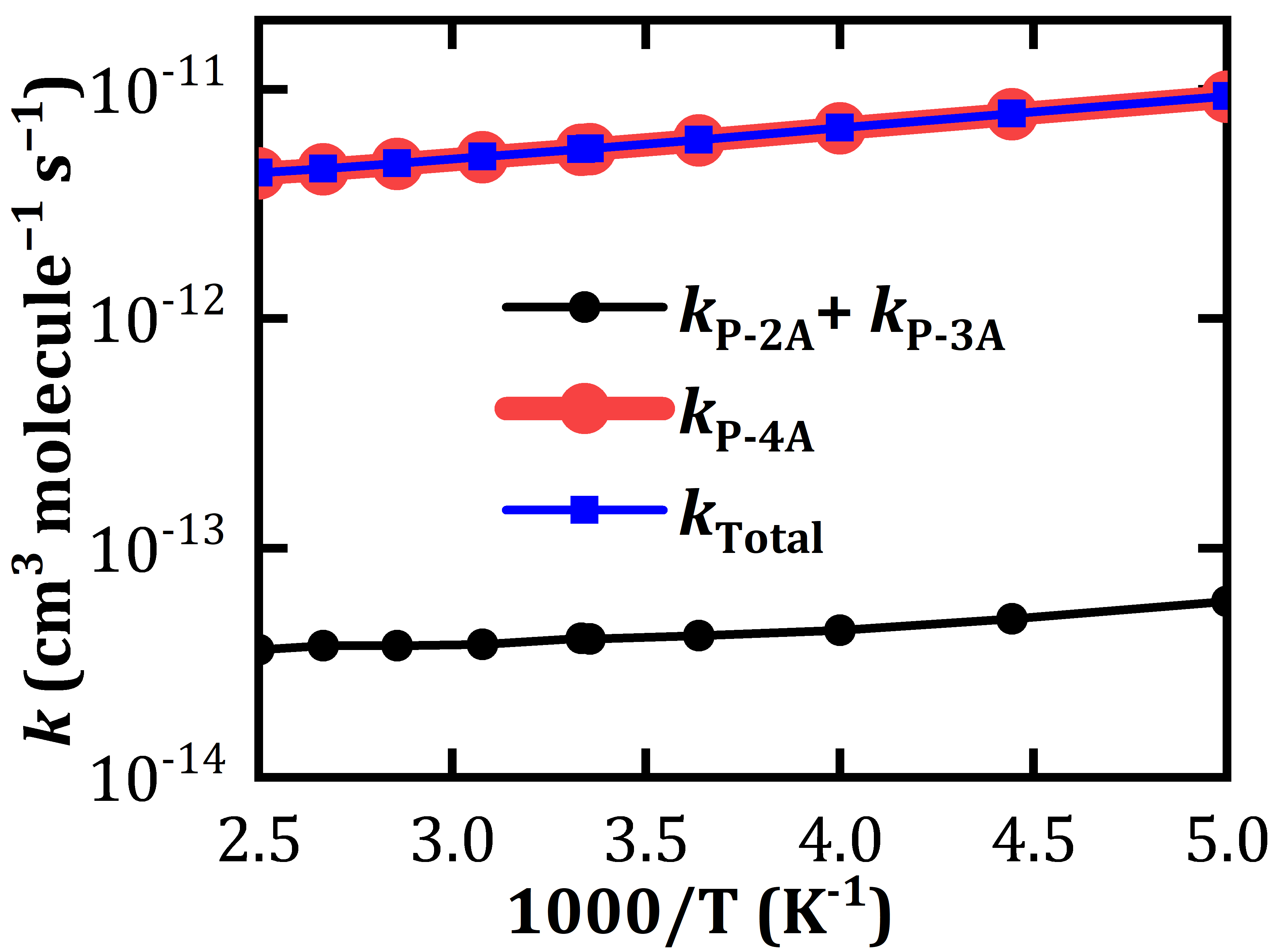


**Figure S6**. Computed rate constants for the NH_2_$\dot{C}$HOH + ^3^O_2_ reaction at 1 atm pressure for the formation of formamide (*k*_P-4A_) and formimidic acid (*k*_P-2A and_ *k*_P-3A_) over the temperature range 200–400 K.


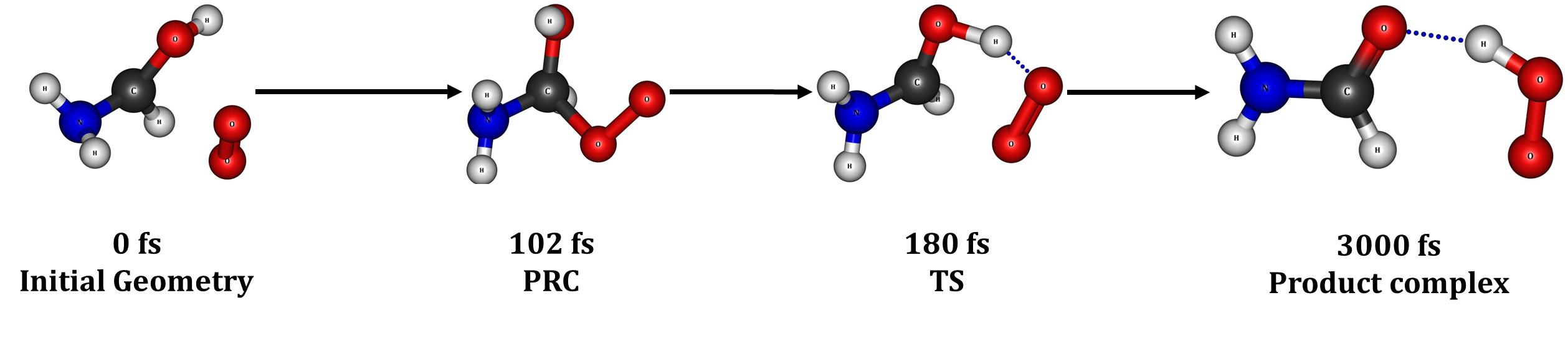


**Figure S7**. Snapshots taken at different time intervals during the BOMD simulations between the NH_2_$\dot{C}$HOH and O_2_.


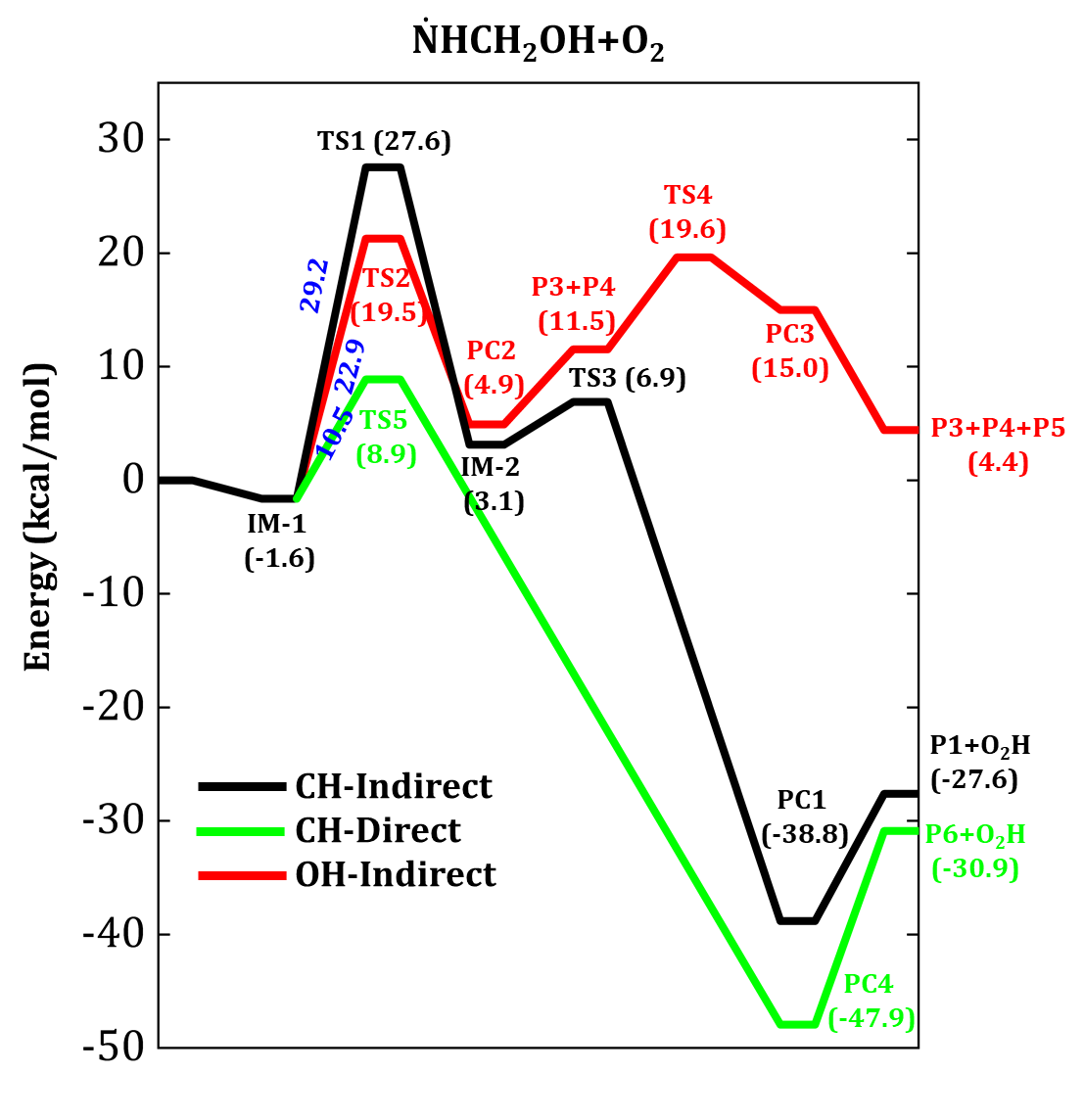


**Figure S8**. The zero-point energy (ZPE) corrected potential energy surface of AM radical ($\dot{N}$HCH_2_OH) + ^3^O_2_ radical. All the energy values are in kcal/mol.

We have computed the potential energy surface (PES) of $\dot{N}$HCH_2_OH+O_2_ reaction and presented Figure S8. The reaction between the $\dot{N}$HCH_2_OH + O_2_ forms an intermediate (IM1), which is stabilized by -1.6 kcal/mol with respect to the reactants. Similar to $\dot{N}$H_2_CHOH+O_2_ reaction, the IM1 undergoes further hydrogen transfer reactions through two indirect (CH-indirect, OH-indirect) and one direct (CH-direct) pathways. The CH-direct reaction pathway proceeds through the TS1 and forms another intermediate, IM2. The IM2 undergoes C-O bond breaking via the TS3 and forms the product complex PC1, which is composed of NHCHOH and O_2_H. The OH-indirect reaction pathway occurred through the TS, namely TS2, and forms product complex PC2. The PC2 is composed of formaldehyde, H_2_CO (P3) and NHOOH (P4). The P4 undergoes further dissociation via the TS4 and form the products P5 (NHO) and P6 ($\dot{O}$H). The simultaneous N-O bond breaking and H transfer from the -CH_2_ group to O of IM1 occurred through TS5. It leads to the formation of product complex PC4 and the corresponding products, P7 (NHCHOH) and P8 (O_2_H).

However, the CH-direct, CH-indirect and OH-indirect reaction pathways shows the high positive energies for the TSs around 8.9 (TS5), 27.6 (TS1) and 19.5 (TS2) kcal/mol and hindered by the strong positive energy barriers, which are around 10.5, 29.2 and 22.9 kcal/mol, respectively. The large positive energies for these TSs are not encouraging and the corresponding reaction pathways will likely not be traversed in the upper atmosphere.

**Table S1**. Calculated energy parameters of PRC, TS, PORC, products (PROD) and the activation energy (*E*_a_) for hydrogen abstraction reaction from different H-bearing groups of three rotational conformers of aminomethanol.

| **(AM)-XH** | **PRC** | **TS** | **PORC** | **PROD** | ***E*_a_** |
| --- | --- | --- | --- | --- | --- |
| AM0-CH | -5.26 | 1.24 | -26.01 | -21.26 | 6.50 |
| AM0-NH_a_ | -5.26 | 0.44 | -21.18 | -16.39 | 5.70 |
| AM0-NH_b_ | -5.26 | -0.56 | -20.63 | -16.39 | 4.70 |
| AM0-OH | -5.26 | 1.67 | -17.44 | -13.48 | 6.93 |
|  |  |  |  |  |  |
| AM1-CH_a_ | -4.59 | 0.57 | -27.12 | -22.84 | 5.16 |
| AM1-CH_b_ | -5.76 | 0.54 | -26.90 | -22.84 | 6.30 |
| AM1-CH_c_ | -5.76 | -0.31 | -26.88 | -22.84 | 5.45 |
| AM1-NH | -4.59 | -0.83 | -18.79 | -16.42 | 3.76 |
| AM1-OH | -4.59 | 2.59 | -16.75 | -13.67 | 7.19 |
|  |  |  |  |  |  |
| AM2-CH | -5.54 | -1.42 | -27.02 | -23.99 | 4.12 |
| AM2-NH | -5.54 | 0.08 | -21.46 | -18.07 | 5.62 |
| AM2-OH_a_ | -5.54 | 0.95 | -16.42 | -12.04 | 6.49 |
| AM2-OH_b_ | -5.54 | 3.71 | -16.42 | -12.04 | 9.25 |

**Table S2**. Calculated temperature dependent rate constants for the $\dot{O}$H initiated oxidation reaction of aminomethanol using the rotational conformation, AM0.

| **1000/T(K^-1^)** | **AM0-CH** | **AM0-NH_a_** | **AM0-NH_b_** | **AM0-OH** | **Total-*k*_OH_** |
| --- | --- | --- | --- | --- | --- |
| 5.00 | 1.71 × 10^-13^ | 1.77 × 10^-13^ | 4.43 × 10^-12^ | 2.20 × 10^-13^ | 5.00 × 10^-12^ |
| 4.76 | 1.65 × 10^-13^ | 1.63 × 10^-13^ | 3.42 × 10^-12^ | 1.72 × 10^-13^ | 3.92 × 10^-12^ |
| 4.55 | 1.63 × 10^-13^ | 1.53 × 10^-13^ | 2.73 × 10^-12^ | 1.43 × 10^-13^ | 3.19 × 10^-12^ |
| 4.35 | 1.64 × 10^-13^ | 1.45 × 10^-13^ | 2.25 × 10^-12^ | 1.23 × 10^-13^ | 2.68 × 10^-12^ |
| 4.17 | 1.66 × 10^-13^ | 1.40 × 10^-13^ | 1.90 × 10^-12^ | 1.10 × 10^-13^ | 2.32 × 10^-12^ |
| 4.00 | 1.70 × 10^-13^ | 1.35 × 10^-13^ | 1.64 × 10^-12^ | 1.01 × 10^-13^ | 2.04 × 10^-12^ |
| 3.85 | 1.76 × 10^-13^ | 1.32 × 10^-13^ | 1.44 × 10^-12^ | 9.44 × 10^-14^ | 1.84 × 10^-12^ |
| 3.70 | 1.83 × 10^-13^ | 1.3 0× 10^-13^ | 1.28 × 10^-12^ | 9.01 × 10^-14^ | 1.69 × 10^-12^ |
| 3.57 | 1.91 × 10^-13^ | 1.28 × 10^-13^ | 1.16 × 10^-12^ | 8.73 × 10^-14^ | 1.56 × 10^-12^ |
| 3.45 | 1.99 × 10^-13^ | 1.27 × 10^-13^ | 1.06 × 10^-12^ | 8.56 × 10^-14^ | 1.47 × 10^-12^ |
| 3.33 | 2.09 × 10^-13^ | 1.26 × 10^-13^ | 9.76 × 10^-13^ | 8.47 × 10^-14^ | 1.40 × 10^-12^ |
| 3.23 | 2.19 × 10^-13^ | 1.25 × 10^-13^ | 9.08 × 10^-13^ | 8.45 × 10^-14^ | 1.34 × 10^-12^ |
| 3.13 | 2.30 × 10^-13^ | 1.25 × 10^-13^ | 8.51 × 10^-13^ | 8.48 × 10^-14^ | 1.29 × 10^-12^ |
| 2.50 | 3.44 × 10^-13^ | 1.33 × 10^-13^ | 6.16 × 10^-13^ | 9.97 × 10^-14^ | 1.19 × 10^-12^ |
| 2.22 | 4.39 × 10^-13^ | 1.43 × 10^-13^ | 5.61 × 10^-13^ | 1.16 × 10^-13^ | 1.26 × 10^-12^ |
| 2.00 | 5.54 × 10^-13^ | 1.55 × 10^-13^ | 5.37 × 10^-13^ | 1.37 × 10^-13^ | 1.38 × 10^-12^ |
| 1.82 | 6.90 × 10^-13^ | 1.69 × 10^-13^ | 5.32 × 10^-13^ | 1.61 × 10^-13^ | 1.55 × 10^-12^ |
| 1.67 | 8.48 × 10^-13^ | 1.85 × 10^-13^ | 5.38 × 10^-13^ | 1.90 × 10^-13^ | 1.76 × 10^-12^ |
| 1.54 | 1.03 × 10^-12^ | 2.04 × 10^-13^ | 5.53 × 10^-13^ | 2.22 × 10^-13^ | 2.01 × 10^-12^ |
| 1.43 | 1.24 × 10^-12^ | 2.24 × 10^-13^ | 5.76 × 10^-13^ | 2.58 × 10^-13^ | 2.30 × 10^-12^ |
| 1.33 | 1.48 × 10^-12^ | 2.46 × 10^-13^ | 6.05 × 10^-13^ | 3.00 × 10^-13^ | 2.63 × 10^-12^ |
| 1.25 | 1.74 × 10^-12^ | 2.70 × 10^-13^ | 6.39 × 10^-13^ | 3.45 × 10^-13^ | 3.00 × 10^-12^ |
| 1.18 | 2.04 × 10^-12^ | 2.95 × 10^-13^ | 6.77 × 10^-13^ | 3.96 × 10^-13^ | 3.41 × 10^-12^ |
| 1.11 | 2.37 × 10^-12^ | 3.23 × 10^-13^ | 7.21 × 10^-13^ | 4.51 × 10^-13^ | 3.87 × 10^-12^ |
| 1.05 | 2.74 × 10^-12^ | 3.53 × 10^-13^ | 7.69 × 10^-13^ | 5.13 × 10^-13^ | 4.38 × 10^-12^ |
| 1.00 | 3.15 × 10^-12^ | 3.85 × 10^-13^ | 8.22 × 10^-13^ | 5.79 × 10^-13^ | 4.93 × 10^-12^ |

**Table S3**. Calculated temperature dependent rate constants for the $\dot{O}$H initiated oxidation reaction of aminomethanol using the rotational conformation, AM1.

| **1000/T(K^-1^)** | **AM1-CH_a_** | **AM1-CH_b_** | **AM1-CH_c_** | **AM1-NH** | **AM1-OH** |
| --- | --- | --- | --- | --- | --- |
| 5.00 | 5.93E-13 | 3.35E-13 | 2.68E-12 | 2.59E-11 | 4.55E-14 |
| 4.76 | 5.31E-13 | 3.35E-13 | 2.27E-12 | 1.77E-11 | 3.79E-14 |
| 4.55 | 4.87E-13 | 3.38E-13 | 1.98E-12 | 1.26E-11 | 3.31E-14 |
| 4.35 | 4.56E-13 | 3.42E-13 | 1.77E-12 | 9.38E-12 | 3.01E-14 |
| 4.17 | 4.33E-13 | 3.48E-13 | 1.60E-12 | 7.21E-12 | 2.82E-14 |
| 4.00 | 4.17E-13 | 3.55E-13 | 1.47E-12 | 5.72E-12 | 2.71E-14 |
| 3.85 | 4.06E-13 | 3.64E-13 | 1.37E-12 | 4.64E-12 | 2.66E-14 |
| 3.70 | 3.98E-13 | 3.73E-13 | 1.29E-12 | 3.85E-12 | 2.65E-14 |
| 3.57 | 3.94E-13 | 3.84E-13 | 1.23E-12 | 3.26E-12 | 2.68E-14 |
| 3.45 | 3.91E-13 | 3.95E-13 | 1.17E-12 | 2.80E-12 | 2.73E-14 |
| 3.33 | 3.91E-13 | 4.07E-13 | 1.13E-12 | 2.45E-12 | 2.81E-14 |
| 3.23 | 3.92E-13 | 4.20E-13 | 1.09E-12 | 2.17E-12 | 2.91E-14 |
| 3.13 | 3.95E-13 | 4.34E-13 | 1.06E-12 | 1.94E-12 | 3.03E-14 |
| 2.50 | 4.48E-13 | 5.70E-13 | 9.68E-13 | 1.07E-12 | 4.51E-14 |
| 2.22 | 5.03E-13 | 6.78E-13 | 9.79E-13 | 8.74E-13 | 5.88E-14 |
| 2.00 | 5.70E-13 | 8.03E-13 | 1.02E-12 | 7.71E-13 | 7.60E-14 |
| 1.82 | 6.49E-13 | 9.46E-13 | 1.08E-12 | 7.15E-13 | 9.68E-14 |
| 1.67 | 7.39E-13 | 1.11E-12 | 1.16E-12 | 6.88E-13 | 1.22E-13 |
| 1.54 | 8.42E-13 | 1.29E-12 | 1.25E-12 | 6.79E-13 | 1.51E-13 |
| 1.43 | 9.57E-13 | 1.50E-12 | 1.36E-12 | 6.84E-13 | 1.85E-13 |
| 1.33 | 1.08E-12 | 1.72E-12 | 1.48E-12 | 6.97E-13 | 2.23E-13 |
| 1.25 | 1.22E-12 | 1.97E-12 | 1.62E-12 | 7.19E-13 | 2.67E-13 |
| 1.18 | 1.38E-12 | 2.24E-12 | 1.77E-12 | 7.47E-13 | 3.17E-13 |
| 1.11 | 1.55E-12 | 2.54E-12 | 1.93E-12 | 7.81E-13 | 3.73E-13 |
| 1.05 | 1.73E-12 | 2.87E-12 | 2.10E-12 | 8.20E-13 | 4.35E-13 |
| 1.00 | 1.93E-12 | 3.22E-12 | 2.29E-12 | 8.63E-13 | 5.04E-13 |

**Table S4**. Calculated temperature dependent rate constants for the $\dot{O}$H initiated oxidation reaction of aminomethanol using the rotational conformation, AM2.

| **1000/T(K^-1^)** | **AM2-CH** | **AM2-NH** | **AM2-OH_a_** | **AM2-OH_b_** |
| --- | --- | --- | --- | --- |
| 5.00 | 7.70E-11 | 1.09E-12 | 8.92E-12 | 2.57E-14 |
| 4.76 | 5.74E-11 | 8.99E-13 | 5.44E-12 | 1.92E-14 |
| 4.55 | 4.44E-11 | 7.64E-13 | 3.53E-12 | 1.53E-14 |
| 4.35 | 3.55E-11 | 6.67E-13 | 2.43E-12 | 1.29E-14 |
| 4.17 | 2.91E-11 | 5.95E-13 | 1.75E-12 | 1.14E-14 |
| 4.00 | 2.45E-11 | 5.40E-13 | 1.31E-12 | 1.05E-14 |
| 3.85 | 2.09E-11 | 4.97E-13 | 1.02E-12 | 1.00E-14 |
| 3.70 | 1.82E-11 | 4.64E-13 | 8.19E-13 | 9.76E-15 |
| 3.57 | 1.61E-11 | 4.37E-13 | 6.75E-13 | 9.74E-15 |
| 3.45 | 1.44E-11 | 4.15E-13 | 5.71E-13 | 9.88E-15 |
| 3.33 | 1.30E-11 | 3.98E-13 | 4.93E-13 | 1.02E-14 |
| 3.23 | 1.19E-11 | 3.84E-13 | 4.33E-13 | 1.06E-14 |
| 3.13 | 1.10E-11 | 3.72E-13 | 3.86E-13 | 1.11E-14 |
| 2.50 | 7.20E-12 | 3.34E-13 | 2.30E-13 | 1.92E-14 |
| 2.22 | 6.30E-12 | 3.35E-13 | 2.04E-13 | 2.77E-14 |
| 2.00 | 5.84E-12 | 3.47E-13 | 1.97E-13 | 3.95E-14 |
| 1.82 | 5.64E-12 | 3.67E-13 | 1.99E-13 | 5.50E-14 |
| 1.67 | 5.59E-12 | 3.92E-13 | 2.08E-13 | 7.48E-14 |
| 1.54 | 5.66E-12 | 4.22E-13 | 2.22E-13 | 9.95E-14 |
| 1.43 | 5.81E-12 | 4.56E-13 | 2.40E-13 | 1.30E-13 |
| 1.33 | 6.02E-12 | 4.95E-13 | 2.61E-13 | 1.66E-13 |
| 1.25 | 6.29E-12 | 5.39E-13 | 2.85E-13 | 2.09E-13 |
| 1.18 | 6.61E-12 | 5.86E-13 | 3.13E-13 | 2.60E-13 |
| 1.11 | 6.97E-12 | 6.38E-13 | 3.44E-13 | 3.19E-13 |
| 1.05 | 7.37E-12 | 6.95E-13 | 3.78E-13 | 3.87E-13 |
| 1.00 | 7.81E-12 | 7.55E-13 | 4.16E-13 | 4.64E-13 |

**Table S5**. Calculated temperature dependent total rate constants for AM0+$\dot{O}$H, AM1+$\dot{O}$H and AM2+$\dot{O}$H radical reactions with and without BSSE corrections.

| **Temp (K)** | **AM0** | | **AM1** | | **AM2** | |
| --- | --- | --- | --- | --- | --- | --- |
|  | **Without BSSE** | **With  BSSE** | **Without BSSE** | **With  BSSE** | **Without BSSE** | **With  BSSE** |
| 200 | 5.00E-12 | 4.48E-12 | 2.95E-11 | 2.04E-11 | 8.70E-11 | 8.02E-11 |
| 210 | 3.92E-12 | 3.58E-12 | 2.08E-11 | 1.53E-11 | 6.37E-11 | 5.97E-11 |
| 220 | 3.19E-12 | 2.96E-12 | 1.55E-11 | 1.19E-11 | 4.87E-11 | 4.62E-11 |
| 230 | 2.68E-12 | 2.52E-12 | 1.20E-11 | 9.59E-12 | 3.86E-11 | 3.70E-11 |
| 240 | 2.32E-12 | 2.20E-12 | 9.62E-12 | 7.98E-12 | 3.15E-11 | 3.04E-11 |
| 250 | 2.04E-12 | 1.96E-12 | 7.99E-12 | 6.81E-12 | 2.63E-11 | 2.55E-11 |
| 260 | 1.84E-12 | 1.77E-12 | 6.81E-12 | 5.94E-12 | 2.25E-11 | 2.19E-11 |
| 270 | 1.69E-12 | 1.63E-12 | 5.94E-12 | 5.29E-12 | 1.95E-11 | 1.91E-11 |
| 280 | 1.56E-12 | 1.52E-12 | 5.29E-12 | 4.78E-12 | 1.72E-11 | 1.69E-11 |
| 290 | 1.47E-12 | 1.43E-12 | 4.79E-12 | 4.39E-12 | 1.54E-11 | 1.51E-11 |
| 300 | 1.40E-12 | 1.37E-12 | 4.40E-12 | 4.09E-12 | 1.39E-11 | 1.37E-11 |
| 310 | 1.34E-12 | 1.31E-12 | 4.10E-12 | 3.84E-12 | 1.27E-11 | 1.26E-11 |
| 320 | 1.29E-12 | 1.27E-12 | 3.86E-12 | 3.65E-12 | 1.18E-11 | 1.16E-11 |
| 400 | 1.19E-12 | 1.18E-12 | 3.10E-12 | 3.04E-12 | 7.78E-12 | 7.73E-12 |
| 450 | 1.26E-12 | 1.25E-12 | 3.09E-12 | 3.05E-12 | 6.86E-12 | 6.83E-12 |
| 500 | 1.38E-12 | 1.38E-12 | 3.24E-12 | 3.21E-12 | 6.43E-12 | 6.40E-12 |
| 550 | 1.55E-12 | 1.55E-12 | 3.49E-12 | 3.47E-12 | 6.26E-12 | 6.24E-12 |
| 600 | 1.76E-12 | 1.76E-12 | 3.82E-12 | 3.81E-12 | 6.27E-12 | 6.25E-12 |
| 650 | 2.01E-12 | 2.01E-12 | 4.22E-12 | 4.21E-12 | 6.40E-12 | 6.39E-12 |
| 700 | 2.30E-12 | 2.29E-12 | 4.68E-12 | 4.67E-12 | 6.63E-12 | 6.62E-12 |
| 750 | 2.63E-12 | 2.62E-12 | 5.21E-12 | 5.20E-12 | 6.94E-12 | 6.93E-12 |
| 800 | 3.00E-12 | 2.99E-12 | 5.80E-12 | 5.79E-12 | 7.32E-12 | 7.32E-12 |
| 850 | 3.41E-12 | 3.41E-12 | 6.45E-12 | 6.45E-12 | 7.77E-12 | 7.76E-12 |
| 900 | 3.87E-12 | 3.87E-12 | 7.17E-12 | 7.17E-12 | 8.27E-12 | 8.26E-12 |
| 950 | 4.38E-12 | 4.38E-12 | 7.95E-12 | 7.95E-12 | 8.83E-12 | 8.82E-12 |
| 1000 | 4.93E-12 | 4.93E-12 | 8.80E-12 | 8.80E-12 | 9.45E-12 | 9.44E-12 |

**Table S6**. Cartesian coordinates of the equilibrium geometries of aminomethanol (AM1, AM2 and AM3), PRCs, TSs, PORCs, carbon, nitrogen and oxygen-centred radicals of $\dot{O}$H + aminomethanol oxidation reaction.

| **Coordinates of**  **AM0+**$\dot{\mathbf{O}}$**H radical reaction** | **Coordinates of**  **AM1+**$\dot{\mathbf{O}}$**H radical reaction** | **Coordinates of**  **AM2+**$\dot{\mathbf{O}}$**H radical reaction** |
| --- | --- | --- |
| ----------------------------------------------------  **OH**  ----------------------------------------------------  8 -0.000000 -0.000000 0.107875  1 0.000000 0.000000 -0.862998  ----------------------------------------------------  **NH_2_CH_2_OH (AM0)**  ----------------------------------------------------  6 0.034187 0.536847 0.048296  1 0.073605 1.076009 0.996362  1 0.078915 1.259495 -0.762486  1 1.275474 -0.852442 0.638752  8 1.192836 -0.263635 -0.114569  7 -1.219928 -0.158548 -0.020096  1 -1.284707 -0.702983 -0.870874  1 -1.351604 -0.782248 0.765691  ----------------------------------------------------  **PRC**  ----------------------------------------------------  6 -1.080853 -0.082281 0.352046  1 -0.924092 -0.109026 1.426715  1 -2.137400 -0.248530 0.143932  1 0.253447 1.440017 0.085085  1 -0.859072 1.311536 -1.133283  7 -0.704765 1.201784 -0.140534  8 -0.326529 -1.196172 -0.148364  1 -0.559756 -1.346973 -1.067587  8 2.099957 0.149218 0.069245  1 1.457923 -0.590198 0.049553  ----------------------------------------------------  **TS-CH**  ----------------------------------------------------  6 0.417585 0.005829 0.459890  1 -0.670003 -0.402953 0.435801  1 0.685278 0.108258 1.512298  1 1.113141 -0.993420 -1.168719  1 2.239269 -0.854925 0.056056  7 1.258732 -0.938067 -0.170554  8 0.282645 1.258982 -0.173981  1 1.150240 1.661790 -0.267829  8 -2.013123 -0.439774 -0.041779  1 -1.990728 0.459072 -0.406987  ----------------------------------------------------  **TS-NH_a_**  ----------------------------------------------------  6 -0.967176 -0.272405 0.386067  1 -1.945790 -0.743662 0.294321  1 -0.729421 -0.169001 1.447491  1 -0.068452 -1.111214 -1.219142  1 1.030016 -0.860033 0.028520  7 0.017610 -1.149896 -0.205222  8 -1.058730 0.970524 -0.251155  1 -0.155868 1.304403 -0.334326  8 1.782814 0.369952 -0.004650  1 1.756634 0.539400 0.949731  ----------------------------------------------------  **TS-NH_b_**  ----------------------------------------------------  6 -0.951269 0.365889 0.352642  1 -0.807628 0.292913 1.427005  1 -1.880901 0.906217 0.154237  1 1.113420 0.668311 0.099696  1 0.104578 1.119559 -1.216482  7 0.134148 1.131606 -0.199487  8 -1.044752 -0.968974 -0.112299  1 -1.398684 -0.977461 -1.004693  8 1.849600 -0.406436 0.086982  1 1.199005 -1.122848 0.023335  ----------------------------------------------------  **TS-OH**  ----------------------------------------------------  6 -0.872652 -0.434573 0.383095  1 -0.581287 -0.334450 1.429290  1 -1.653930 -1.194963 0.318309  1 -0.689425 1.555146 0.047711  1 -1.627352 0.778154 -1.059840  7 -1.371766 0.818468 -0.081756  8 0.200046 -0.985093 -0.355784  1 0.925440 -0.236304 -0.519950  8 1.812196 0.534904 0.045964  1 2.366885 -0.087916 0.536766  ----------------------------------------------------  **PORC-CH**  ----------------------------------------------------  6 0.967415 0.146826 0.441872  1 -1.447717 -0.335868 0.829387  1 1.869876 0.335559 1.003498  1 0.086854 -1.230973 -0.759460  1 1.714125 -1.500301 -0.526875  7 0.862869 -1.129816 -0.119732  8 0.547115 1.248123 -0.253433  1 -0.356312 1.088419 -0.554662  8 -1.909607 -0.170857 -0.001552  1 -2.811466 0.052788 0.234875  ----------------------------------------------------  **PORC-NH_a_**  ----------------------------------------------------  6 0.998177 0.046956 0.445252  1 2.076784 0.121486 0.632592  1 0.496619 0.006474 1.416485  1 0.682462 1.050854 -1.224872  1 -1.576312 0.818740 0.102117  7 0.599217 1.251742 -0.223575  8 0.779069 -1.124748 -0.288351  1 -0.177814 -1.237592 -0.338634  8 -1.888200 -0.093365 0.096135  1 -2.812272 -0.058988 -0.156445  ----------------------------------------------------  **PORC-NH_b_**  ----------------------------------------------------  6 -1.289550 -0.036587 0.219117  1 -1.423856 -0.038023 1.307128  1 -2.273886 -0.215988 -0.223899  1 2.789030 0.075651 0.624278  1 0.167819 1.213680 -0.326223  7 -0.842604 1.256412 -0.157144  8 -0.419942 -1.124891 -0.066018  1 -0.451014 -1.306680 -1.008668  8 2.093518 0.152546 -0.030056  1 1.438826 -0.525236 0.181283  ----------------------------------------------------  **PORC-OH**  ----------------------------------------------------  6 1.236142 0.149980 0.216741  1 1.339996 0.298451 1.304067  1 2.245837 0.296168 -0.190053  1 -0.157397 -1.322643 0.303288  1 0.761787 -1.360074 -1.050914  7 0.779556 -1.176555 -0.056136  8 0.467572 1.208096 -0.199721  1 -1.418032 0.516201 -0.249355  8 -2.065285 -0.138313 0.040966  1 -2.864228 0.349637 0.245508  ----------------------------------------------------  **NH_2_**$\dot{\mathbf{C}}$**HOH**  ----------------------------------------------------  6 0.048156 0.507867 -0.162392  1 0.127467 1.534219 0.162101  1 -1.313475 -0.953996 -0.557877  1 -1.498983 -0.266079 0.945270  7 -1.193276 -0.113017 -0.011144  8 1.211811 -0.181109 0.069200  1 1.054502 -1.121354 -0.050735  ----------------------------------------------------  $\dot{\mathbf{N}}$**HCH_2_OH**  ----------------------------------------------------  6 -0.055963 0.508620 0.007715  1 -0.096805 1.159012 -0.874239  1 -0.030908 1.166655 0.883417  1 -1.044539 -1.190286 -0.248869  7 -1.269861 -0.234743 0.040930  8 1.132210 -0.239143 -0.104899  1 1.339380 -0.630752 0.746079  ----------------------------------------------------  **NH2CH2**$\dot{\mathbf{O}}$  ----------------------------------------------------  6 -0.133512 0.469759 -0.000004  1 -0.249321 1.128191 0.873487  1 -0.249321 1.128195 -0.873490  1 1.323010 -0.684400 0.816744  1 1.322981 -0.684451 -0.816718  7 1.184923 -0.102589 -0.000004  8 -1.205092 -0.373496 0.000003  ----------------------------------------------------  **H_2_O**  ----------------------------------------------------  8 0.000000 0.000000 0.116660  1 -0.000000 0.760849 -0.466641  1 -0.000000 -0.760849 -0.466641  ---------------------------------------------------- | ----------------------------------------------------  **NH_2_CH_2_OH (AM1)**  ----------------------------------------------------  6 0.033391 0.535408 -0.000000  1 0.091157 1.168233 -0.887902  1 0.091136 1.168166 0.887932  1 -1.307322 -0.730190 -0.816350  1 -1.307313 -0.730198 0.816344  7 -1.224734 -0.137107 0.000000  8 1.107720 -0.400195 -0.000007  1 1.943375 0.072851 0.000035  ----------------------------------------------------  **PRC1**  ----------------------------------------------------  6 -1.120569 0.143674 0.338028  1 -2.150986 0.412102 0.104942  1 -0.972418 0.183826 1.417638  1 -0.997400 -1.257618 -1.115112  1 0.083067 -1.466467 0.109656  7 -0.859961 -1.173887 -0.116650  8 -0.241430 1.096754 -0.293996  1 -0.466720 1.986424 -0.009320  8 2.214075 -0.208925 0.092571  1 1.466437 0.394260 -0.108023  ----------------------------------------------------  **PRC2**  ----------------------------------------------------  6 0.674320 -0.397618 -0.000002  1 0.397513 -0.970645 0.886967  1 0.397517 -0.970640 -0.886977  1 0.160536 1.380760 -0.815686  1 0.160528 1.380753 0.815693  7 -0.076564 0.827590 0.000000  8 2.061813 -0.113672 -0.000000  1 2.558827 -0.935229 0.000013  8 -2.725370 -0.320558 0.000001  1 -1.876433 0.181420 -0.000007  ----------------------------------------------------  **TS-CH_a_**  ----------------------------------------------------  6 0.458074 0.045828 0.483830  1 -0.650057 -0.251139 0.597082  1 0.845553 0.223916 1.488714  1 0.707981 -1.267311 -1.022725  1 2.105222 -0.870383 -0.244543  7 1.115879 -1.048123 -0.122941  8 0.515514 1.218695 -0.300663  1 0.185633 1.961690 0.212498  8 -2.008893 -0.345779 0.004908  1 -1.806896 0.281786 -0.707385  ----------------------------------------------------  **TS-CH_b_**  ----------------------------------------------------  6 0.427545 -0.068059 0.380278  1 -0.544417 -0.589901 0.088343  1 0.481558 -0.100168 1.470702  1 1.601396 -0.600275 -1.166268  1 2.397494 -0.543584 0.276141  7 1.521791 -0.775294 -0.172923  8 0.318109 1.244917 -0.098510  1 -0.615090 1.481688 -0.099389  8 -2.083661 -0.354510 -0.172760  1 -2.414328 -0.935594 0.529420  ----------------------------------------------------  **TS-CH_c_**  ----------------------------------------------------  6 -0.410381 -0.064006 0.406567  1 0.635595 -0.504230 0.219733  1 -0.564728 -0.080898 1.487533  1 -0.291238 1.286851 -1.073506  1 -1.231666 1.770985 0.178311  7 -0.381280 1.277297 -0.064021  8 -1.364893 -0.838104 -0.270064  1 -1.333162 -1.744237 0.048682  8 2.091996 -0.393810 -0.089966  1 2.099620 0.569798 0.028229  ----------------------------------------------------  **TS-NH**  ----------------------------------------------------  6 0.934570 0.379095 0.355915  1 1.859672 0.941240 0.193781  1 0.761119 0.269743 1.427114  1 -0.100380 1.077189 -1.220818  1 -1.117302 0.662978 0.095769  7 -0.144957 1.126959 -0.205166  8 1.025953 -0.895964 -0.267801  1 1.771470 -1.380696 0.094919  8 -1.845160 -0.429329 0.119113  1 -1.213642 -1.131387 -0.100584  ----------------------------------------------------  **TS-OH**  ----------------------------------------------------  6 -0.640328 0.475359 -0.054985  1 -0.486162 1.016951 -0.987833  1 -0.398485 1.127454 0.783631  1 -2.265196 -0.517307 -0.754644  1 -2.177538 -0.455528 0.881694  7 -2.008593 0.066859 0.031157  8 0.183864 -0.678928 -0.025264  1 1.154621 -0.424976 0.329613  8 2.257943 0.190806 0.101879  1 2.540423 -0.161784 -0.753563  ----------------------------------------------------  **PORC-CH_a_**  ----------------------------------------------------  6 0.649325 -0.223591 0.270625  1 -1.618899 -0.908798 -0.275734  1 0.435593 -0.660099 1.241341  1 2.175024 0.186266 -0.964640  1 2.659659 -0.578155 0.437921  7 1.916947 -0.466828 -0.239120  8 0.166996 1.034950 0.028637  1 -0.795674 1.006180 0.111762  8 -2.316698 -0.299303 -0.003378  1 -2.972660 -0.321229 -0.702632  ----------------------------------------------------  **PORC-CH_b_**  ----------------------------------------------------  6 0.657004 -0.216185 0.303755  1 -1.610139 -0.889663 -0.316541  1 0.480138 -0.618523 1.296532  1 2.136849 0.143293 -1.001034  1 2.673492 -0.563902 0.413394  7 1.906364 -0.478579 -0.239674  8 0.167682 1.034148 0.036267  1 -0.794570 1.009738 0.123106  8 -2.319880 -0.251796 -0.168622  1 -2.954761 -0.692601 0.398577  ----------------------------------------------------  **PORC-CH_c_**  ----------------------------------------------------  6 -0.729357 -0.315356 0.093873  1 2.734802 -0.829909 -0.745146  1 -0.337874 -0.987680 0.847059  1 -0.531471 1.505458 -0.719838  1 -0.061616 1.454375 0.864813  7 -0.104953 0.929965 -0.005113  8 -2.087109 -0.298817 -0.100091  1 -2.419914 -1.199592 -0.108250  8 2.581532 -0.296243 0.035510  1 1.771504 0.200211 -0.149436  ----------------------------------------------------  **PORC-NH**  ----------------------------------------------------  6 0.982795 0.053489 0.457492  1 2.054100 0.052676 0.708113  1 0.417060 0.034760 1.395404  1 0.584549 1.049306 -1.204205  1 -1.820487 0.881118 0.056471  7 0.708692 1.270694 -0.211755  8 0.652942 -1.064977 -0.349765  1 0.825128 -1.878927 0.130496  8 -2.096640 -0.037708 0.093209  1 -1.368377 -0.533246 -0.296494  ----------------------------------------------------  **PORC-OH**  ----------------------------------------------------  6 1.026315 -0.209304 0.509500  1 2.083208 -0.401195 0.698010  1 0.457373 -0.191212 1.440309  1 1.341451 1.077955 -1.054016  1 -0.116471 1.287749 -0.282721  7 0.868066 1.069581 -0.158475  8 0.490869 -1.072872 -0.421392  1 -1.396900 -0.566210 -0.204563  8 -1.958524 0.154265 0.114555  1 -2.861773 -0.089470 -0.089998  ----------------------------------------------------  **NH_2_**$\dot{\mathbf{C}}$**HOH**  ----------------------------------------------------  6 0.037657 0.495299 -0.153450  1 0.085815 1.507152 0.231431  1 -1.143145 -1.118052 -0.286162  1 -1.708326 0.010519 0.798317  7 -1.193677 -0.134579 -0.062029  8 1.121693 -0.319153 0.100244  1 1.921915 0.123863 -0.190629  ----------------------------------------------------  $\dot{\mathbf{N}}$**HCH_2_OH**  ----------------------------------------------------  6 0.051424 0.505351 -0.000002  1 0.043301 1.159524 0.883698  1 0.043308 1.159508 -0.883714  1 1.048803 -1.198920 -0.000021  7 1.276217 -0.200974 0.000005  8 -1.062816 -0.361857 0.000002  1 -1.874946 0.149452 -0.000003  ----------------------------------------------------  **NH2CH2**$\dot{\mathbf{O}}$  ----------------------------------------------------  6 -0.133512 0.469759 -0.000004  1 -0.249321 1.128191 0.873487  1 -0.249321 1.128195 -0.873490  1 1.323010 -0.684400 0.816744  1 1.322981 -0.684451 -0.816718  7 1.184923 -0.102589 -0.000004  8 -1.205092 -0.373496 0.000003 | ----------------------------------------------------  **NH_2_CH_2_OH (AM2)**  ----------------------------------------------------  6 -0.042841 0.538699 0.016216  1 -0.029947 1.193185 -0.859675  1 -0.087577 1.161660 0.911876  1 -1.109757 -0.924546 -0.614668  8 -1.208491 -0.240874 0.054386  7 1.124716 -0.314296 -0.060386  1 1.937912 0.190771 -0.385486  1 1.341340 -0.726200 0.838274  ----------------------------------------------------  **PRC**  ----------------------------------------------------  6 0.929349 0.165618 0.463179  1 0.428336 0.097293 1.430287  1 1.973926 0.435127 0.625184  1 1.649223 -1.663964 -0.111407  1 0.005087 -1.534421 -0.182371  7 0.880346 -1.042782 -0.318279  8 0.251133 1.209496 -0.217408  1 0.552240 1.186141 -1.131601  8 -2.109960 -0.291653 0.078489  1 -1.476715 0.442848 -0.069865  ----------------------------------------------------  **TS-CH**  ----------------------------------------------------  6 0.446614 0.029613 0.488242  1 -0.624150 -0.368263 0.554274  1 0.812553 0.135317 1.512909  1 1.607575 -1.634265 0.293662  1 0.873176 -1.154341 -1.102572  7 1.311976 -0.833554 -0.247679  8 0.302321 1.263642 -0.153790  1 1.171380 1.531036 -0.468900  8 -2.021823 -0.427956 -0.041578  1 -1.948027 0.462226 -0.422119  ----------------------------------------------------  **TS-NH**  ----------------------------------------------------  6 -0.913729 0.321987 0.403660  1 -0.602749 0.160747 1.437618  1 -1.848064 0.893365 0.406446  1 0.312440 1.920645 0.097387  1 0.962176 0.475756 -0.500924  7 0.054194 1.059965 -0.378315  8 -1.087976 -0.955334 -0.142820  1 -1.310621 -0.856507 -1.073441  8 1.877120 -0.393636 0.103449  1 1.276683 -1.153931 0.174121  ----------------------------------------------------  **TS-OH_a_**  ----------------------------------------------------  6 0.855253 0.447640 0.347984  1 0.670810 0.372545 1.422750  1 1.719607 1.106615 0.190572  1 1.392143 -0.871716 -1.121838  1 1.706531 -1.418971 0.395772  7 1.048627 -0.896629 -0.168997  8 -0.176045 1.088849 -0.311335  1 -1.116647 0.532756 -0.153102  8 -1.786807 -0.458809 0.112011  1 -1.141538 -1.170985 -0.044488  ----------------------------------------------------  **TS-OH_b_**  ----------------------------------------------------  6 0.826049 0.444370 0.394204  1 0.527194 0.223121 1.421087  1 1.504766 1.306263 0.420377  1 2.039401 -0.540395 -0.940362  1 1.838501 -1.338051 0.492108  7 1.396399 -0.737900 -0.187293  8 -0.264014 0.912049 -0.341492  1 -0.954150 0.097475 -0.486004  8 -1.885389 -0.580974 0.050139  1 -2.491582 0.102068 0.369441  ----------------------------------------------------  **PORC-CH**  ----------------------------------------------------  6 1.073088 0.106443 0.433714  1 -1.403121 0.564716 0.159884  1 2.066319 0.262679 0.837822  1 1.387837 -1.859417 0.098466  1 -0.135693 -1.312540 -0.324204  7 0.851537 -1.081769 -0.254983  8 0.486179 1.209954 -0.145898  1 0.510792 1.107823 -1.108090  8 -2.011783 -0.166704 -0.005990  1 -2.620580 -0.175533 0.733824  ----------------------------------------------------  **PORC-NH**  ----------------------------------------------------  6 -0.986743 0.010211 0.433482  1 -0.450204 0.075012 1.385742  1 -2.064893 -0.021183 0.657556  1 -0.989415 1.975366 0.140850  1 1.760474 0.746845 -0.475224  7 -0.714576 1.141561 -0.383899  8 -0.568670 -1.182163 -0.176613  1 -0.870175 -1.173730 -1.089828  8 2.078069 0.058601 0.114649  1 1.461508 -0.666010 -0.036974  ----------------------------------------------------  **PORC-OH**  ----------------------------------------------------  6 0.993261 0.177820 0.397175  1 0.558097 0.197453 1.405848  1 2.075400 0.372814 0.531421  1 1.202027 -1.218020 -1.088592  1 0.904820 -1.872461 0.389511  7 0.688189 -1.097528 -0.224679  8 0.540217 1.238613 -0.301116  1 -2.037003 0.714836 -0.371531  8 -2.062166 -0.109567 0.118240  1 -1.304641 -0.611213 -0.213941  ----------------------------------------------------  **NH_2_**$\dot{\mathbf{C}}$**HOH**  ----------------------------------------------------  6 0.038723 0.519105 -0.140865  1 0.044166 1.521739 0.269969  1 -1.943582 0.323350 0.199033  1 -1.275154 -0.946785 -0.618087  7 -1.114491 -0.242374 0.092481  8 1.210802 -0.169807 -0.021006  1 1.057248 -0.957862 0.514961  ----------------------------------------------------  $\dot{\mathbf{N}}$**HCH_2_OH**  ----------------------------------------------------  6 -0.050036 0.489132 0.026338  1 -0.092984 1.208399 -0.801891  1 -0.122709 1.072682 0.960501  1 -2.002814 0.126775 -0.007900  7 -1.138868 -0.419283 -0.026831  8 1.180744 -0.167570 -0.047995  1 1.044849 -1.067098 0.263043  ----------------------------------------------------  **NH2CH2**$\dot{\mathbf{O}}$  ----------------------------------------------------  6 0.131029 0.451696 0.053208  1 0.186401 1.279181 -0.669448  1 0.261663 0.934401 1.047364  1 -1.242965 -0.951454 0.600749  1 -1.907831 0.389134 -0.077151  7 -1.121703 -0.246797 -0.115800  8 1.221060 -0.329233 -0.051270  ---------------------------------------------------- |

**Table S7**. Vibrational frequencies and rotational constants of aminomethanol (AM0, AM1, AM2) + $\dot{O}$H radical oxidation reaction.

| **Vibrational frequencies and rotational constants of**  **AM0+**$\dot{\mathbf{O}}$**H radical reaction** | **Vibrational frequencies and Rotational constants of**  **AM1+**$\dot{\mathbf{O}}$**H radical reaction** | **Vibrational frequencies and Rotational constants of**  **AM2+**$\dot{\mathbf{O}}$**H radical reaction** |
| --- | --- | --- |
| --------------------------------------------------  **OH**  --------------------------------------------------  ***Vibrational frequencies (cm^-1^):***  3792.84870  ***Rotational constant (GHz):***  565.5157  --------------------------------------------------  **CH_2_NH_2_OH(AM0)**  --------------------------------------------------  ***Vibrational frequencies (cm^-1^):***  244.5092 377.2802 483.6988  810.6144 912.6089 1051.6192  1115.8288 1168.9726 1370.1898  1391.1463 1431.5507 1518.0279  1659.3462 3065.7995 3137.9280  3543.7856 3629.2319 3895.2867  ***Rotational constants (GHz):***  38.6564 9.6586 8.6662  --------------------------------------------------  **PRC**  --------------------------------------------------  ***Vibrational frequencies (cm^-1^):***  36.7688 88.1229 206.8893  278.9573 412.1830 427.1129  508.6282 566.5223 780.6234  910.1537 1003.5535 1125.4085  1165.0197 1371.0195 1390.8766  1426.6323 1512.9630 1651.1576  3083.9796 3152.0138 3539.2610  3621.0351 3635.9660 3891.1813  ***Rotational constants (GHz):***  8.9710 4.6560 3.3369  --------------------------------------------------  **TS-CH**  --------------------------------------------------  ***Vibrational frequencies (cm^-1^):***  839.1479$i$ 67.7986 148.3168  163.7839 272.5789 350.4520  480.3649 656.1616 728.3609  866.7595 1010.0998 1134.4149  1182.2828 1239.2777 1302.5435  1365.2028 1418.3303 1635.8718  1726.2861 3088.4937 3561.4233  3662.6460 3793.3958 3875.2533  ***Rotational constants (GHz):***  9.5005 4.5756 3.4029  --------------------------------------------------  **TS-NH_a_**  --------------------------------------------------  ***Vibrational frequencies (cm^-1^):***  672.0147$i$ 160.5616 200.6401  243.5872 383.7195 487.0779  579.8249 674.3981 826.6585  959.0988 1048.0743 1115.9508  1207.3571 1321.1035 1389.6702  1440.9974 1516.2588 1598.2624  2292.5311 3051.3157 3106.4560  3526.8740 3796.7304 3798.8530  ***Rotational constants (GHz):***  10.2578 5.2282 3.8932  --------------------------------------------------  **TS-NH_b_**  --------------------------------------------------  ***Vibrational frequencies (cm^-1^):***  1250.2488$i$ 142.3768 171.2323  292.3306 419.9473 439.0880  523.6106 751.1275 853.4648  939.0064 1052.7567 1099.3744  1129.6576 1335.6816 1368.8100  1422.1358 1485.2118 1505.5874  1747.1592 3046.8818 3149.2843  3529.5710 3792.0386 3894.0295  ***Rotational constants (GHz):***  10.3164 5.0979 3.7659  --------------------------------------------------  **TS-OH**  --------------------------------------------------  ***Vibrational frequencies (cm^-1^):***  1179.1052$i$ 92.4979 179.2780  222.7315 376.9878 417.1574  503.1541 777.3268 824.5010  911.2713 1017.8444 1134.4310  1210.7106 1302.9631 1373.8959  1398.7862 1485.5213 1657.8531  1791.7782 3057.7898 3100.6088  3544.5351 3631.4152 3833.3301  ***Rotational constants (GHz):***  11.4469 4.6333 3.6652  --------------------------------------------------  **PORC-CH**  --------------------------------------------------  ***Vibrational frequencies (cm^-1^):***  86.1708 130.2865 171.4369  178.7096 204.4658 334.1592  352.5441 509.3863 556.8768  685.2295 760.7957 1067.4906  1143.4099 1225.3273 1362.4578  1441.7075 1617.1505 1636.9999  3221.0065 3504.8466 3630.4345  3804.4831 3825.0400 3959.1827  ***Rotational constants (GHz):***  9.1689 4.6791 3.3648  --------------------------------------------------  **PORC-NH_a_**  --------------------------------------------------  ***Vibrational frequencies (cm^-1^):***  122.3753 136.0103 141.6241  186.7578 292.1640 304.0034  412.1866 510.0217 568.2671  917.3804 1002.1613 1116.8503  1209.3516 1269.5582 1411.8660  1417.8324 1471.5428 1618.2471  3007.7685 3058.5873 3443.8957  3821.3712 3832.6766 3961.0115  ***Rotational constants (GHz):***  9.3069 4.6841 3.4857  --------------------------------------------------  **PORC-NH_b_**  --------------------------------------------------  ***Vibrational frequencies (cm^-1^):***  64.2472 131.8594 163.9562  193.2938 247.8626 362.2217  410.8791 525.8186 577.0331  915.9422 993.3085 1105.1670  1196.6029 1275.8072 1373.7543  1403.5328 1463.2546 1615.4071  3016.2070 3053.6042 3439.2595  3790.2531 3890.8118 3959.4954  ***Rotational constants (GHz):***  9.9926 4.0574 3.0349  --------------------------------------------------  **PORC-OH**  --------------------------------------------------  ***Vibrational frequencies (cm^-1^):***  63.4288 140.8463 154.6652  180.7807 278.4272 362.8707  471.4586 533.8285 751.1445  868.9267 982.9788 1083.7496  1163.6601 1343.0201 1358.0835  1403.4983 1625.3890 1666.7687  2961.8563 3007.2783 3538.0568  3622.6845 3804.4033 3958.4636  ***Rotational constants (GHz):***  9.7568 4.1868 3.0838  --------------------------------------------------  **NH2CHOH**  --------------------------------------------------  ***Vibrational frequencies (cm^-1^):***  209.4208 261.5173 487.0733  647.3946 716.3698 1072.0307  1120.8163 1240.6167 1293.9828  1427.5856 1632.3339 3220.7534  3484.6026 3613.3295 3890.7687  ***Rotational constants (GHz):***  50.4261 10.0798 8.8648  --------------------------------------------------  $\dot{\mathbf{N}}$**HCH_2_OH**  --------------------------------------------------  ***Vibrational frequencies (cm^-1^):***  272.7795 328.5371 508.5303  909.9010 989.8401 1120.2623  1196.0551 1236.6626 1373.9047  1400.2540 1461.3669 3009.3136  3040.8889 3454.8677 3902.5320  ***Rotational constants (GHz):***  43.2921 10.4389 9.1189  --------------------------------------------------  **NH_2_CH_2_**$\dot{\mathbf{O}}$  --------------------------------------------------  ***Vibrational frequencies (cm^-1^):***  236.8183 507.3466 654.0908  841.4129 996.9817 1026.0177  1145.2796 1347.7302 1348.8310  1402.5292 1659.3439 2973.6909  2997.5357 3544.5129 3626.2204  ***Rotational constants (GHz):***  44.8725 10.1725 9.1590  --------------------------------------------------  **H_2_O**  --------------------------------------------------  ***Vibrational frequencies (cm^-1^):***  1623.0861 3887.1501 3988.0256  ***Rotational constants (GHz):***  829.7790 433.1164 284.5769 | --------------------------------------------------  **CH_2_NH_2_OH(AM1)**  --------------------------------------------------  ***Vibrational frequencies (cm^-1^):***  185.6377 353.0714 472.1103  858.3565 945.7151 1040.9756  1106.6104 1255.9495 1270.3062  1393.5671 1448.3110 1536.8367  1668.3654 3045.0984 3086.4762  3544.0224 3623.3221 3891.8786  ***Rotational constants (GHz):***  39.2441 9.9150 8.7158  --------------------------------------------------  **PRC1**  --------------------------------------------------  ***Vibrational frequencies (cm^-1^):***  53.1549 103.2974 204.9639  264.8706 390.8425 442.5543  490.4736 660.1137 840.0086  949.6503 988.6255 1114.5325  1259.5815 1274.6640 1396.5051  1444.9113 1534.3769 1669.9937  3063.1223 3110.3697 3542.6861  3576.0147 3630.1117 3885.3922  ***Rotational constants (GHz):***  9.3644 4.2123 3.1337  --------------------------------------------------  **PRC2**  --------------------------------------------------  ***Vibrational frequencies (cm^-1^):***  48.2877 55.6507 189.3455  190.6637 353.9879 485.0539  601.0811 704.2616 940.8526  948.2870 1065.8161 1104.4507  1254.1252 1269.3022 1393.7102  1456.1344 1539.3282 1663.4412  3051.8008 3095.2674 3442.3405  3532.1865 3608.2488 3895.6553  ***Rotational constants (GHz):***  22.0967 2.4559 2.2683  --------------------------------------------------  **TS-CH_a_**  --------------------------------------------------  ***Vibrational frequencies (cm^-1^):***  840.7905$i$ 78.4138 130.8352  222.3359 294.6213 395.7738  476.3613 718.0117 796.7563  875.0684 1030.2919 1126.7196  1191.7936 1272.0031 1351.4384  1370.5399 1439.2120 1650.2646  1842.0036 3076.2487 3542.6891  3629.4613 3791.9527 3878.3115  ***Rotational constants (GHz):***  9.0054 4.7264 3.4662  --------------------------------------------------  **TS-CH_b_**  --------------------------------------------------  ***Vibrational frequencies (cm^-1^):***  637.2077$i$ 96.5239 119.6071  141.3632 274.3381 370.8509  494.4109 628.1140 756.5124  905.1815 958.2242 1103.9267  1145.2842 1275.4189 1324.6372  1419.6523 1457.0997 1650.4857  2093.3044 3065.9700 3551.4386  3640.1514 3805.7064 3860.8591  ***Rotational constants (GHz):***  10.9799 3.9626 3.1541  --------------------------------------------------  **TS-CH_c_**  --------------------------------------------------  ***Vibrational frequencies (cm^-1^):***  706.5968$i$ 115.1103 127.8165  213.4278 297.0125 383.2458  485.6900 681.6007 832.8195  910.9691 1066.0228 1115.5561  1145.7136 1247.6961 1322.8768  1405.5623 1449.1718 1652.7056  1853.3428 3070.7473 3533.8338  3614.9556 3788.6321 3884.6541  ***Rotational constants (GHz):***  9.7448 4.2403 3.2078  --------------------------------------------------  **TS-NH**  --------------------------------------------------  ***Vibrational frequencies (cm^-1^):***  1195.3688$i$ 143.9512 173.7436  273.9623 380.6696 430.0618  517.5294 773.2606 861.2597  957.1487 1033.7960 1100.6839  1221.4347 1250.3574 1340.7819  1433.7978 1491.6848 1517.3959  1788.3340 3029.1686 3095.5276  3531.6065 3798.1723 3893.1572  ***Rotational constants (GHz):***  10.4962 5.1007 3.7962  --------------------------------------------------  **TS-OH**  --------------------------------------------------  ***Vibrational frequencies (cm^-1^):***  1249.7791$i$ 61.2804 138.9075  230.1058 363.3912 371.7111  497.1679 780.7514 837.0166  944.5963 1033.1739 1134.4202  1230.8595 1282.2430 1377.6354  1413.8301 1530.3804 1664.7271  1687.4891 3070.5727 3121.4981  3546.2461 3630.7183 3837.6312  ***Rotational constants (GHz):***  28.7431 3.0533 2.8811  --------------------------------------------------  **PORC-CH_a_**  --------------------------------------------------  ***Vibrational frequencies (cm^-1^):***  75.8936 99.1826 175.8575  198.1804 209.0792 271.3423  404.2222 504.3463 615.4453  660.1052 812.1221 1065.3836  1191.6956 1220.4790 1361.0180  1457.6998 1614.9986 1628.7777  3140.3338 3538.7144 3654.4809  3778.3862 3807.0831 3949.4798  ***Rotational constants (GHz):***  16.9039 2.9378 2.6310  --------------------------------------------------  **PORC-CH_b_**  --------------------------------------------------  ***Vibrational frequencies (cm^-1^):***  72.1479 103.5645 130.4785  192.5839 210.3810 250.1916  431.5178 503.9930 613.2155  655.8822 811.3006 1065.7109  1188.3021 1219.9560 1354.9561  1452.3010 1619.4556 1630.0832  3133.9030 3540.2687 3656.0638  3782.4219 3808.7728 3953.4323  ***Rotational constants (GHz):***  16.6281 2.9354 2.6371  --------------------------------------------------  **PORC-CHc**  --------------------------------------------------  ***Vibrational frequencies (cm^-1^):***  44.1289 48.3519 133.9909  170.3480 205.9132 349.6842  416.4145 489.3028 602.2765  737.6690 815.9680 1078.5626  1140.1009 1215.6932 1307.9355  1435.5059 1635.9637 1645.5085  3182.4852 3478.6641 3608.7207  3735.4524 3898.4230 3953.0584  ***Rotational constants (GHz):***  19.0151 2.4936 2.2609  --------------------------------------------------  **PORC-NH**  --------------------------------------------------  ***Vibrational frequencies (cm^-1^):***  77.1302 100.9791 162.1390  201.1974 228.1937 336.7229  368.8155 458.0753 505.3124  963.2707 985.7581 1111.9007  1171.1659 1243.7144 1312.2457  1430.9389 1471.4824 1646.2756  2973.2224 3036.3466 3450.9871  3833.6838 3892.5421 3939.6694  ***Rotational constants (GHz):***  9.0678 4.3679 3.3020  --------------------------------------------------  **PORC-OH**  --------------------------------------------------  ***Vibrational frequencies (cm^-1^):***  102.9990 128.0943 157.4604  213.5778 362.4311 397.7056  434.6995 553.0048 842.1300  961.5144 989.2457 1164.5152  1223.8541 1352.3913 1360.2121  1537.4152 1618.1633 1648.0717  3054.5873 3111.3333 3514.2146  3619.1648 3756.0976 3963.4288  ***Rotational constants (GHz):***  10.3162 4.3840 3.5130  --------------------------------------------------  **NH_2_**$\dot{\mathbf{C}}$**HOH**  --------------------------------------------------  ***Vibrational frequencies (cm^-1^):***  221.3749 358.6369 483.8598  678.6598 759.2191 1063.0577  1135.3019 1210.3927 1314.3578  1438.7816 1634.4768 3163.9369  3516.9438 3640.5751 3904.8662  ***Rotational constants (GHz):***  52.7279 10.2328 8.9375  --------------------------------------------------  $\dot{\mathbf{N}}$**HCH_2_OH**  --------------------------------------------------  ***Vibrational frequencies (cm^-1^):***  142.0427 281.3721 499.6134  966.5188 988.2051 1121.3250  1175.4762 1248.3372 1306.2022  1429.6089 1472.4352 2973.1498  2994.3534 3461.4583 3901.3938  ***Rotational constants (GHz):***  44.3818 10.7112 9.1189  --------------------------------------------------  **NH_2_CH_2_**$\dot{\mathbf{O}}$  --------------------------------------------------  ***Vibrational frequencies (cm^-1^):***  236.8183 507.3466 654.0908  841.4129 996.9817 1026.0177  1145.2796 1347.7302 1348.8310  1402.5292 1659.3439 2973.6909  2997.5357 3544.5129 3626.2204  ***Rotational constants (GHz):***  44.8725 10.1725 9.1590 | --------------------------------------------------  **CH_2_NH_2_OH(AM2)**  --------------------------------------------------  ***Vibrational frequencies (cm^-1^):***  245.8977 376.3549 540.8600  813.9427 971.2386 1033.8312  1145.2202 1226.8264 1309.5281  1391.3996 1461.6354 1542.5546  1642.5352 3034.7402 3074.6239  3549.1369 3639.0327 3873.0562  ***Rotational constants (GHz):***  39.1781 9.9521 8.7315  --------------------------------------------------  **PRC**  --------------------------------------------------  ***Vibrational frequencies (cm^-1^):***  88.2743 119.1598 204.1426  269.2105 343.8336 467.8566  581.0814 641.3008 801.2859  958.7353 1035.8968 1132.2729  1218.3837 1318.8381 1387.0936  1458.3917 1534.4517 1639.9139  3054.8112 3097.7757 3546.0072  3586.4205 3645.5732 3860.9336  ***Rotational constants (GHz):***  9.0504 4.5391 3.3705  --------------------------------------------------  **TS-CH**  --------------------------------------------------  ***Vibrational frequencies (cm^-1^):***  765.6022$i$ 62.9606 139.8178  260.0603 280.7640 355.6558  530.9975 701.9561 802.6655  896.7519 1001.7781 1171.8690  1234.2426 1271.3430 1344.4620  1401.6907 1443.0990 1642.5475  1948.6219 3057.0676 3546.0040  3641.8140 3789.2323 3864.2985  ***Rotational constants (GHz):***  9.4799 4.5334 3.4172  --------------------------------------------------  **TS-NH**  --------------------------------------------------  ***Vibrational frequencies (cm^-1^):***  838.5127$i$ 114.3509 186.5734  326.9909 361.0789 490.6801  515.3982 666.6283 844.9190  1003.6420 1032.4382 1135.7573  1227.5345 1231.9575 1386.6984  1432.3022 1526.1487 1548.4409  1924.4566 3021.7677 3076.4520  3542.2500 3778.0020 3870.6429  ***Rotational constants (GHz):***  10.3113 4.9707 3.7582  --------------------------------------------------**TS-OH_a_**  --------------------------------------------------  ***Vibrational frequencies (cm^-1^):***  1652.2492$i$ 166.7692 212.5771  266.1288 429.2737 477.2058  556.7006 702.0590 844.0296  892.1537 994.0216 1042.7450  1133.7444 1244.4397 1301.5110  1434.3713 1493.4404 1574.0999  1634.7009 2993.9524 3063.9908  3538.3379 3628.2005 3720.7459  ***Rotational constants (GHz):***  10.3343 5.4007 3.9388  --------------------------------------------------**TS-OH_b_**  --------------------------------------------------  ***Vibrational frequencies (cm^-1^):***  1426.7754$i$ 101.9637 143.8314  204.4096 237.1401 423.4678  527.1411 709.2611 798.1638  938.1466 1033.8871 1140.7062  1221.7891 1287.0633 1341.6381  1421.4278 1483.3347 1645.8406  1656.1046 3002.3321 3070.4911  3575.8973 3671.0213 3835.5916  ***Rotational constants (GHz):***  12.4769 4.2580 3.5264  --------------------------------------------------  **PORC-CH**  --------------------------------------------------  ***Vibrational frequencies (cm^-1^):***  116.8425 150.8782 161.6068  201.2369 235.0843 350.2465  466.2487 521.6697 552.2648  755.7232 825.2781 1022.1391  1196.2820 1229.1746 1333.7882  1417.6286 1623.6498 1639.5596  3161.5081 3507.3667 3654.9654  3739.5304 3797.3583 3957.9016  ***Rotational constants (GHz):***  9.4783 4.4300 3.2569  --------------------------------------------------  **PORC-NH**  --------------------------------------------------  ***Vibrational frequencies (cm^-1^):***  92.3499 110.3514 172.8647  212.7915 252.7425 301.7602  453.2435 525.4557 545.6051  887.8592 991.7923 1111.5945  1175.5027 1237.4908 1364.6522  1420.4234 1464.6192 1649.6638  2957.6670 3046.8257 3466.6952  3839.8694 3875.8542 3931.7340  ***Rotational constants (GHz):***  9.0617 4.4572 3.3324  --------------------------------------------------  **PORC-OH**  --------------------------------------------------  ***Vibrational frequencies (cm^-1^):***  79.5301 101.6353 176.8472  188.5665 204.7302 352.5710  538.6770 615.7476 742.4955  831.5511 979.3824 1120.6471  1131.7747 1273.9865 1367.6763  1431.3256 1634.3851 1647.7149  2894.6490 3003.2262 3543.5189  3632.1079 3752.1956 3938.5000  ***Rotational constants (GHz):***  9.0461 4.5052 3.3247  --------------------------------------------------  **NH_2_**$\dot{\mathbf{C}}$**HOH**  --------------------------------------------------  ***Vibrational frequencies (cm^-1^):***  182.3585 380.0916 500.7823  725.4845 805.9434 1027.3927  1169.2208 1238.4126 1340.1365  1422.5169 1634.6357 3161.2570  3530.9182 3648.7606 3786.9675  ***Rotational constants (GHz):***  51.1005 10.4350 8.9952  --------------------------------------------------  $\dot{\mathbf{N}}$**HCH_2_OH**  --------------------------------------------------  ***Vibrational frequencies (cm^-1^):***  164.0920 460.5479 521.1441  888.6337 989.2624 1120.5045  1184.5816 1243.2827 1346.9831  1425.6242 1465.3518 2933.2435  3004.7378 3473.8912 3879.8945  ***Rotational constants (GHz):***  45.2745 10.6627 9.1595  --------------------------------------------------  **NH_2_CH_2_**$\dot{\mathbf{O}}$  --------------------------------------------------  ***Vibrational frequencies (cm^-1^):***  174.3928 514.3799 697.8634  784.2664 973.6745 1108.8381  1149.2380 1262.9672 1363.2136  1432.2389 1633.4609 2838.3774  2981.0562 3544.3861 3633.9951  ***Rotational constants (GHz):***  47.1379 10.3126 9.1132 |

**Table S8**. Cartesian coordinates of the equilibrium geometries of NH_2_$\dot{C}$HOH, O_2_, intermediates (IMs), transition states (TSs), product complexes (PCs) and the products (Ps) of O_2_ + carbon cantered NH_2_$\dot{C}$HOH aminomethanol radical reaction.

| ----------------------------------------------------  **O2**  ----------------------------------------------------  8 0.000000 0.000000 0.593873  8 0.000000 0.000000 -0.593873  ----------------------------------------------------  **NH2CHOH**  ----------------------------------------------------  6 -0.038723 0.519105 0.140865  1 -0.044166 1.521739 -0.269969  1 1.943582 0.323350 -0.199033  1 1.275154 -0.946785 0.618087  7 1.114491 -0.242374 -0.092481  8 -1.210802 -0.169807 0.021006  1 -1.057248 -0.957862 -0.514961  ----------------------------------------------------  **IM-0A**  ----------------------------------------------------  6 0.425902 -0.018728 0.341303  1 0.198816 0.047359 1.406033  1 -0.186073 -1.915028 0.186201  1 0.809280 -1.406184 -1.046846  7 0.613921 -1.345839 -0.054779  8 1.487465 0.735543 -0.074420  1 1.343583 1.655191 0.165003  8 -0.765853 0.619358 -0.307061  8 -1.848921 0.039088 0.084636  ----------------------------------------------------  **TS-1A**  ----------------------------------------------------  6 -0.285937 0.018717 -0.107682  1 0.539674 0.032121 -1.094256  8 -0.791184 1.280466 0.049748  1 -0.050784 1.894527 0.104130  8 0.770988 -0.317916 0.696461  8 1.716917 -0.108183 -0.390376  7 -1.285145 -0.932759 -0.194403  1 -1.124214 -1.768137 0.343038  1 -2.226806 -0.576429 -0.192652  ----------------------------------------------------  **IM-1A**  ----------------------------------------------------  6 -0.437644 0.035547 -0.270799  1 2.014522 -0.492912 -0.888716  8 -0.457443 1.345654 0.061889  1 0.454658 1.656704 0.070589  8 0.594340 -0.700794 0.321971  8 1.819949 -0.086150 -0.033758  7 -1.671154 -0.570552 -0.123330  1 -1.629260 -1.574486 -0.205591  1 -2.170755 -0.278400 0.711004  ----------------------------------------------------  **TS-2A**  ----------------------------------------------------  6 0.416877 0.021390 -0.105108  1 -2.500916 -0.042098 0.318300  8 0.701480 1.341195 0.080035  1 -0.087664 1.842176 -0.145965  8 -0.659537 -0.482221 0.492231  8 -1.827491 -0.126790 -0.369096  7 1.536416 -0.796487 -0.043895  1 1.317857 -1.759309 -0.251447  1 2.298941 -0.451165 -0.608337  ----------------------------------------------------  **PC-1A**  ----------------------------------------------------  6 -0.617912 -0.078477 -0.000004  1 1.826410 -0.692399 -0.000048  8 -0.084696 1.145208 -0.000015  1 0.885704 1.053148 -0.000015  8 0.031560 -1.107144 -0.000014  8 2.511086 0.018579 0.000021  7 -1.964143 -0.039296 0.000009  1 -2.450616 0.837910 0.000043  1 -2.468625 -0.905865 0.000049  ----------------------------------------------------  **P-1A (NH_2_COOH)**  ----------------------------------------------------  6 0.038989 0.122815 -0.001452  8 0.841395 -0.963617 0.001688  1 1.745689 -0.631543 0.003775  8 0.452395 1.251692 0.004040  7 -1.264982 -0.241327 -0.032139  1 -1.524085 -1.201670 0.099141  1 -1.950990 0.481003 0.084940  ----------------------------------------------------  **OH**  ----------------------------------------------------  8 -0.000000 -0.000000 0.107875  1 0.000000 0.000000 -0.862998  ----------------------------------------------------  **TS-3A**  ----------------------------------------------------  6 -0.451211 0.004159 0.320524  1 -0.421623 -0.040400 1.414829  8 0.501295 -0.913543 -0.192830  8 1.682152 -0.200736 0.079746  1 -2.170728 -0.846397 0.395314  8 -1.696244 -0.245359 -0.184166  7 0.044849 1.264065 -0.213453  1 -0.118701 2.031415 0.435437  1 1.206746 0.859080 -0.296564 | ----------------------------------------------------  **IM-2A**  ----------------------------------------------------  6 -0.429620 0.040801 0.291486  1 -0.246809 0.049172 1.377868  8 0.635301 -0.655030 -0.324329  8 1.829906 -0.088072 0.160334  1 -1.486197 -1.558138 0.144627  8 -1.589136 -0.623766 -0.061313  7 -0.455280 1.391662 -0.155106  1 -1.408521 1.735597 -0.018447  1 1.897648 0.721871 -0.364763  ----------------------------------------------------  **TS-4A1**  ----------------------------------------------------  6 -0.656818 0.122666 0.390992  1 -0.280442 -0.087731 1.387090  8 0.800826 -0.485199 -0.469635  8 1.906079 -0.126666 0.234981  1 -1.455785 -1.605352 0.254858  8 -1.603230 -0.717703 -0.084780  7 -0.604073 1.361359 -0.041972  1 -1.183016 1.438076 -0.881419  1 2.259262 0.626032 -0.257203  ----------------------------------------------------  **PC-2A**  ----------------------------------------------------  6 -1.093784 -0.174838 -0.000010  1 -0.531839 -1.104452 -0.000038  8 1.929802 -0.863245 0.000016  8 2.136620 0.422729 -0.000012  1 -2.708626 -1.187688 -0.000020  8 -2.431079 -0.267777 0.000006  7 -0.481991 0.924771 0.000000  1 -1.114910 1.722143 0.000056  1 1.209262 0.811984 -0.000017  ----------------------------------------------------  **P-2A (NHCHOH)**  ----------------------------------------------------  6 0.091652 0.408966 0.000010  7 1.233371 -0.102113 0.000007  1 1.174256 -1.120640 -0.000016  1 -0.032892 1.490051 0.000033  8 -1.063007 -0.299780 -0.000015  1 -1.820818 0.289821 -0.000008  ----------------------------------------------------  **O_2_H**  ----------------------------------------------------  8 0.054974 0.705972 -0.000000  8 0.054974 -0.597832 0.000000  1 -0.879587 -0.865120 0.000000  ----------------------------------------------------  **TS-4A2**  ----------------------------------------------------  6 -0.688103 0.157731 0.348582  1 -0.337788 -0.012995 1.365762  8 0.776744 -0.569208 -0.421672  8 1.883472 -0.143104 0.253902  1 -1.569371 -1.520959 0.349411  8 -1.652975 -0.668281 -0.085449  7 -0.635739 1.335744 -0.232286  1 0.073830 1.891039 0.243658  1 2.354185 0.391059 -0.398564  ----------------------------------------------------  **PC-3A**  ----------------------------------------------------  6 -1.486613 0.182516 0.228674  1 -2.338022 0.240707 0.907126  8 1.780295 -0.491958 0.599122  8 1.783667 0.210819 -0.497644  1 -1.870672 -1.670366 0.024395  8 -1.274418 -1.007961 -0.331933  7 -0.726338 1.143516 -0.053882  1 -0.992156 1.991931 0.430818  1 0.888550 0.650823 -0.513566  ----------------------------------------------------  **P-3A (NHCHOH)**  ----------------------------------------------------  6 -0.093193 0.360462 -0.000000  7 -1.151134 -0.309768 -0.000000  1 -1.958271 0.304648 -0.000000  1 -0.029198 1.451162 0.000001  8 1.099128 -0.265821 0.000000  1 1.811542 0.376361 -0.000002  ----------------------------------------------------  **TS-5A**  ----------------------------------------------------  6 -0.509225 0.017274 0.479769  1 -0.960570 -0.016764 1.474942  8 0.662056 -0.741699 0.533444  8 1.402491 -0.212310 -0.549258  1 -1.596380 -1.334445 -0.368162  8 -1.397977 -0.402821 -0.501890  7 -0.019462 1.343679 0.147030  1 1.035664 0.945893 -0.303128  1 -0.619689 1.750555 -0.569842  ----------------------------------------------------  **TS-7A**  ----------------------------------------------------  6 -0.515490 0.068874 0.368020  1 -0.571950 -0.086792 1.459101  8 0.627601 -0.870567 0.047629  8 1.720110 -0.114674 -0.157155  1 -1.878879 -1.254720 0.044612  8 -1.618602 -0.395071 -0.299233  7 -0.106181 1.333796 0.048738  1 -0.782791 1.796183 -0.549596  1 1.236948 0.838012 -0.233342  ----------------------------------------------------  **IM-3A**  ----------------------------------------------------  6 -0.542429 0.014722 0.427230  1 -0.993670 -0.002273 1.431273  8 0.814962 -0.179638 0.704908  8 1.544504 -0.042128 -0.498679  1 -0.623857 -1.803048 -0.197960  8 -1.065313 -0.972902 -0.401123  7 -0.766905 1.296623 -0.156889  1 1.598285 0.919381 -0.590805  1 -1.711071 1.278590 -0.548513 | ----------------------------------------------------  **TS-6A1**  ----------------------------------------------------  6 -0.825928 0.092948 0.378049  1 -1.064571 0.049030 1.436407  8 0.938910 0.063067 0.712871  8 1.611548 -0.200055 -0.440339  1 -0.847566 -1.807705 0.203430  8 -1.050528 -1.034941 -0.332377  7 -0.905646 1.261717 -0.221480  1 -0.832335 1.104230 -1.230109  1 2.040123 0.640167 -0.648906  ----------------------------------------------------  **TS-6A2**  ----------------------------------------------------  6 0.830528 -0.038229 0.335651  1 1.075787 -0.088041 1.395514  8 -0.907272 -0.394295 0.627485  8 -1.634231 0.223457 -0.341565  1 0.577022 1.840555 0.427054  8 0.871975 1.185780 -0.211910  7 1.054756 -1.038624 -0.499180  1 1.157514 -1.893661 0.044126  1 -1.820557 -0.478645 -0.978420  ----------------------------------------------------  **TS-8A**  ----------------------------------------------------  6 0.498880 0.151850 0.300212  8 -0.620383 -0.843578 -0.020926  8 -1.789912 -0.152205 -0.076014  1 -1.480970 0.755372 -0.306006  8 0.195712 1.333081 -0.065552  7 1.732763 -0.399122 -0.094885  1 1.811652 -1.380288 0.135693  1 1.898637 -0.246428 -1.080853  1 0.364718 0.055717 1.414017  ----------------------------------------------------  **IM-4A**  ----------------------------------------------------  6 0.434911 0.105929 0.291629  8 -0.583488 -0.703343 -0.281231  8 -1.826658 -0.183782 0.137303  1 -1.963207 0.550196 -0.475861  8 0.360837 1.386821 -0.129745  7 1.701250 -0.490075 0.024675  1 1.703046 -1.453220 0.335838  1 1.888766 -0.477188 -0.971024  1 0.247652 0.177596 1.377935  ----------------------------------------------------  **TS-9A**  ----------------------------------------------------  6 0.575311 0.140250 0.377153  1 0.258822 -0.048865 1.405099  8 0.394776 1.313639 -0.127467  8 -0.691330 -0.501756 -0.451882  8 -1.853316 -0.243311 0.227466  1 -2.292173 0.412969 -0.328984  7 1.719943 -0.548051 0.016666  1 1.753790 -1.513393 0.306190  1 1.987056 -0.404428 -0.946821  ----------------------------------------------------  **026-PC-4A**  ----------------------------------------------------  6 1.069966 -0.171807 -0.000024  1 0.524968 -1.121657 -0.000053  8 0.490028 0.899520 -0.000021  8 -1.947671 -0.846836 -0.000004  8 -2.098061 0.447770 0.000024  1 -1.165174 0.796183 0.000003  7 2.405997 -0.286479 0.000003  1 2.849576 -1.186963 0.000085  1 2.974481 0.545007 0.000104  ----------------------------------------------------  **P-4A (NH_2_CHO)**  **----------------------------------------------------**  6 0.163602 0.386729 -0.000005  1 0.145677 1.487939 0.000015  8 1.186720 -0.246722 -0.000001  7 -1.077968 -0.156406 0.000012  1 -1.175059 -1.158160 -0.000016  1 -1.900209 0.418463 -0.000044  ----------------------------------------------------  **TS-10A**  ----------------------------------------------------  6 -0.772801 0.101078 0.410678  7 -0.291540 1.263012 0.059693  1 -0.624860 1.528844 -0.863376  1 -0.628895 -0.262886 1.419846  1 0.881537 1.022841 0.006079  8 1.045846 -0.917609 0.015052  8 1.803734 0.095830 -0.137111  8 -1.720250 -0.480393 -0.317415  1 -1.984840 -1.318976 0.071322  ----------------------------------------------------  **TS-11A**  ----------------------------------------------------  6 -0.855110 0.170409 0.397229  7 -0.408043 1.286559 -0.113931  1 0.656167 0.897718 -0.546580  1 -1.074592 0.042639 1.451465  1 -0.202822 1.958188 0.616571  8 1.039241 -0.737159 0.450207  8 1.614454 0.024367 -0.387514  8 -1.389856 -0.707885 -0.448586  1 -1.502502 -1.561493 -0.020161  ----------------------------------------------------  **TS-12A**  ----------------------------------------------------  6 0.711246 0.158447 0.432032  1 0.717006 -0.062854 1.497425  8 0.203303 1.252306 -0.003615  1 -0.871246 0.958382 -0.205911  8 -0.926320 -0.887485 0.180126  8 -1.729898 -0.002887 -0.261914  7 1.685377 -0.445721 -0.280001  1 2.028163 -1.337285 0.034730  1 1.684283 -0.284352 -1.275203 |
| --- | --- | --- |

**Table S9**. Vibrational frequencies and rotational constants of of O_2_ + carbon cantered NH_2_$\dot{C}$HOH aminomethanol radical reaction.

| --------------------------------------------------  **O2**  --------------------------------------------------  ***Vibrational frequencies (^cm-1^):***  1771.3940  ***Rotational constants (GHz):***  44.7938  --------------------------------------------------  **NH2CHOH**  --------------------------------------------------  ***Vibrational frequencies (cm^-1^):***  182.3585 380.0916 500.7823  725.4845 805.9434 1027.3927  1169.2208 1238.4126 1340.1365  1422.5169 1634.6357 3161.2570  3530.9182 3648.7606 3786.9675  ***Rotational constants (GHz):***  51.1005 10.4350 8.9952  --------------------------------------------------  **IM-0A**  --------------------------------------------------  ***Vibrational frequencies (cm^-1^):***  105.2703 256.3720 338.9327  356.0681 468.3125 537.0850  589.8343 803.4959 867.2661  1041.7142 1169.3906 1242.1419  1314.5905 1367.7394 1374.8708  1490.0030 1645.5878 3103.7586  3552.4204 3650.8800 3893.3226  ***Rotational constants (GHz):***  9.2103 4.3685 3.1976  --------------------------------------------------  **TS-1A**  --------------------------------------------------  ***Vibrational frequencies (cm^-1^):***  1349.1977$i$ 134.1235 232.5825  344.3535 414.1399 451.0854  459.5435 575.8209 747.4881  980.5865 1008.7082 1057.4184  1150.9847 1181.3453 1351.1566  1463.2888 1611.1036 1997.0176  3604.8212 3728.8056 3866.2984  ***Rotational constants (GHz):***  8.5104 4.6065 3.4958  --------------------------------------------------  **IM-1A**  --------------------------------------------------  ***Vibrational frequencies (cm^-1^):***  131.8349 244.5953 267.6870  320.5812 422.7821 443.1576  598.3918 610.4301 688.2089  968.5458 1038.4680 1045.2983  1167.1088 1363.8915 1409.0291  1439.8411 1621.5446 3504.5656  3668.4432 3804.9370 3860.4126  ***Rotational constants (GHz):***  9.8955 4.2333 3.1146  --------------------------------------------------  **TS-2A**  --------------------------------------------------  ***Vibrational frequencies (cm^-1^):***  724.4969$i$ 110.6535 227.5288  267.9794 326.6311 352.7331  460.6117 561.3116 655.1564  808.8325 917.3691 1079.8828  1165.4794 1326.4092 1380.0496  1458.4543 1621.1983 3576.1185  3681.5825 3827.5337 3888.0588  ***Rotational constants (GHz):***  9.1837 4.0825 3.0591  --------------------------------------------------  **PC-1A**  --------------------------------------------------  ***Vibrational frequencies (cm^-1^):***  43.8156 125.0358 175.4913  203.7096 389.4227 515.3988  525.4447 615.6353 638.4683  721.0606 807.4220 1012.2066  1104.3693 1331.2189 1484.6461  1619.4823 1816.8296 3477.5006  3645.3349 3653.3164 3787.1410  ***Rotational constants (GHz):***  11.5334 2.8735 2.3003  --------------------------------------------------  **P-1A (NH_2_COOH)**  --------------------------------------------------  ***Vibrational frequencies (cm^-1^):***  207.6713 480.5410 503.7365  580.0925 602.7897 801.5539  987.4314 1091.6073 1245.2869  1446.4334 1618.3088 1871.2965  3644.8647 3780.2725 3870.7369  ***Rotational constants (GHz):***  11.7558 10.9238 5.6660  --------------------------------------------------  **OH**  --------------------------------------------------  ***Vibrational frequencies (cm^-1^):***  3792.84870  ***Rotational constants (GHz):***  565.5157  --------------------------------------------------  **TS-3A**  --------------------------------------------------  ***Vibrational frequencies (cm^-1^):***  2338.2439$i$ 210.1493 288.9286  406.3965 460.3616 596.7563  669.7768 744.1912 968.5367  1005.5048 1038.4017 1101.0492  1219.6624 1245.3537 1311.6665  1369.6037 1446.9814 1947.5090  3033.7972 3531.7579 3900.9020  ***Rotational constants (GHz):***  10.4680 4.6037 3.4356 | --------------------------------------------------  **IM-2A**  --------------------------------------------------  ***Vibrational frequencies (cm^-1^):***  162.5871 266.2626 300.5175  326.4947 416.0711 447.4597  570.8652 604.3466 974.2161  1024.6158 1078.6427 1160.1968  1215.0189 1259.6319 1369.4469  1427.9252 1456.3983 2975.5527  3481.8913 3795.0338 3874.9586  ***Rotational constants (GHz):***  9.7419 4.2468 3.1627  --------------------------------------------------  **TS-4A1**  --------------------------------------------------  ***Vibrational frequencies (cm^-1^):***  764.4738$i$ 133.0153 210.4972  335.4094 366.2852 421.8689  448.4141 596.0476 734.8376  1029.9045 1100.3469 1137.5476  1197.8187 1292.1316 1395.9987  1441.6914 1505.7765 3162.9381  3478.2507 3803.1631 3879.9719  ***Rotational constants (GHz):***  9.5967 3.6699 2.9362  --------------------------------------------------  **PC-2A**  --------------------------------------------------  ***Vibrational frequencies (cm^-1^):***  73.1840 81.3348 123.0184  195.8761 289.1284 439.5859  614.3910 798.6201 888.0060  1084.3269 1143.2504 1188.5725  1310.1819 1329.8877 1431.6581  1651.6114 1781.9094 3037.4363  3183.7208 3527.7997 3895.5693  ***Rotational constants (GHz):***  14.6342 1.9815 1.7452  --------------------------------------------------  **P-2A (NHCHOH)**  --------------------------------------------------  ***Vibrational frequencies (cm^-1^):***  414.8738 587.7101 887.9768  1077.1501 1098.3532 1179.0397  1317.5886 1429.3026 1800.6393  3144.8051 3504.9838 3900.2774  ***Rotational constants (GHz):***  71.4501 11.4097 9.8386  --------------------------------------------------  **O_2_H**  --------------------------------------------------  ***Vibrational frequencies (cm^-1^):***  1269.8394 1468.4698 3702.0144  ***Rotational constants (GHz):***  630.4409 34.9207 33.0880  --------------------------------------------------  **TS-4A2**  --------------------------------------------------  ***Vibrational frequencies (cm^-1^):***  777.5239$i$ 108.2380 205.5553  347.4195 375.7474 423.6055  453.6409 618.4234 706.9573  1006.3055 1093.9202 1117.9777  1199.6221 1308.2212 1370.9892  1426.5709 1535.9898 3109.6554  3518.4239 3806.9383 3897.7461  ***Rotational constants (GHz):***  9.6724 3.6581 2.9100  --------------------------------------------------  **PC-3A**  --------------------------------------------------  ***Vibrational frequencies (cm^-1^):***  39.8289 71.0109 87.3428  178.9498 279.0163 412.3630  610.4801 765.0868 829.6063  1057.2054 1109.3634 1210.9858  1303.4506 1339.4883 1445.0541  1597.0628 1788.0369 3114.2986  3173.8460 3595.9605 3912.9072  ***Rotational constants (GHz):***  8.4008 2.7250 2.3082  --------------------------------------------------  **P-3A (NHCHOH)**  --------------------------------------------------  ***Vibrational frequencies (cm^-1^):***  391.9387 625.2952 833.6835  1057.5908 1071.9859 1201.0016  1335.9849 1432.4383 1796.5293  3082.2952 3571.3544 3919.2676  ***Rotational constants (GHz):***  80.3165 11.1920 9.8231  --------------------------------------------------  **TS-5A**  --------------------------------------------------  ***Vibrational frequencies (cm^-1^):***  2305.9690$i$ 164.3992 234.8611  433.6782 471.7190 569.7297  742.0977 838.3049 959.2869  1010.2746 1041.9192 1063.3507  1156.0338 1216.1652 1300.1191  1397.7244 1408.5085 1939.8769  3073.2566 3519.4673 3879.7886  ***Rotational constants (GHz):***  8.1173 5.2069 4.1780  --------------------------------------------------  **TS-7A**  --------------------------------------------------  ***Vibrational frequencies (cm^-1^):***  2984.5109$i$ 135.9181 276.8318  315.1203 415.6626 462.8385  572.0154 760.6731 846.0042  959.7229 1057.2586 1152.4612  1192.8002 1230.7069 1257.6488  1310.6646 1433.3511 2120.7285  2944.6842 3575.2784 3879.5961  ***Rotational constants (GHz):***  9.8551 4.5564 3.3634  --------------------------------------------------  **IM-3A**  --------------------------------------------------  ***Vibrational frequencies (cm^-1^):***  156.7229 221.1967 272.7301  300.7617 386.7799 475.6306  524.3265 771.2677 926.2861  1017.1296 1071.7269 1111.7938  1221.8241 1244.6749 1357.4999  1436.0758 1447.6745 2994.1472  3479.6975 3800.1515 3880.5271  ***Rotational constants (GHz):***  7.8030 4.7977 3.8687 | --------------------------------------------------  **TS-6A1**  --------------------------------------------------  ***Vibrational frequencies (cm^-1^):***  821.3139$i$ 97.7712 239.7073  276.7500 398.2908 413.3863  515.2419 610.3827 700.7720  967.7984 1097.6475 1168.6000  1206.4454 1289.1622 1392.3785  1436.2549 1497.9374 3157.0778  3466.2060 3803.5994 3876.9195  ***Rotational constants (GHz):***  7.8318 4.2442 3.4829  --------------------------------------------------  **TS-6A2**  --------------------------------------------------  ***Vibrational frequencies (cm^-1^):***  780.6271$i$ 106.0889 256.3863  272.4847 382.1119 421.5219  512.7655 639.5872 682.0633  967.6404 1099.6829 1148.8328  1199.9365 1303.2800 1367.2073  1438.4644 1525.1327 3109.0566  3527.0239 3805.9065 3892.3690  ***Rotational constants (GHz):***  7.7957 4.3992 3.4513  --------------------------------------------------  **TS-8A**  --------------------------------------------------  ***Vibrational frequencies (cm^-1^):***  1315.0426$i$ 136.8275 285.8717  308.2062 401.9520 557.2973  614.7593 653.7795 738.3970  890.9287 1056.7738 1128.1041  1183.0025 1265.5314 1427.4071  1487.5319 1628.9869 2694.5091  3355.2909 3558.6893 3657.2024  ***Rotational constants (GHz):***  10.1755 4.3132 3.2090  --------------------------------------------------  **IM-4A**  --------------------------------------------------  ***Vibrational frequencies (cm^-1^):***  165.9191 227.7177 283.4112  364.6420 435.0389 543.0600  593.1245 821.4135 943.8716  1040.3740 1060.9040 1117.3204  1235.7748 1287.3794 1371.1272  1437.2076 1649.0169 2948.2756  3546.4932 3633.0416 3817.1182  ***Rotational constants (GHz):***  9.8747 4.1952 3.1684  --------------------------------------------------  **TS-9A**  --------------------------------------------------  ***Vibrational frequencies (cm^-1^):***  694.8713$i$ 148.8313 240.8491  384.4649 394.0678 407.7905  482.4240 609.1889 641.0465  1021.0759 1068.6768 1203.7599  1280.8977 1315.8455 1436.9368  1478.3614 1633.4038 3088.7262  3577.3090 3689.2566 3815.3950  ***Rotational constants (GHz):***  10.4072 3.8209 3.1097  --------------------------------------------------  **PC-4A**  --------------------------------------------------  ***Vibrational frequencies (cm^-1^):***  58.8218 63.9256 120.2920  182.8728 264.8220 344.3143  599.1371 663.4268 739.8594  1080.2019 1084.5641 1305.4208  1312.1073 1427.5648 1605.4704  1622.0498 1788.4461 3085.2955  3220.1156 3610.2301 3752.2228  ***Rotational constants (GHz):***  15.4614 2.0363 1.7993  --------------------------------------------------  **P-4A (NH_2_CHO)**  --------------------------------------------------  ***Vibrational frequencies (cm^-1^):***  193.0662 573.1537 630.1559  1060.5143 1061.1713 1279.0341  1430.3004 1614.8009 1846.4114  3008.5542 3615.1101 3758.8238  ***Rotational constants (GHz):***  73.8418 11.4863 9.9401  --------------------------------------------------  **TS-10A**  --------------------------------------------------  ***Vibrational frequencies (cm^-1^):***  869.839$i$ 146.1976 207.0073  423.6897 499.2496 536.3460  562.4289 687.2764 903.1458  1040.4022 1137.7201 1199.8990  1310.0248 1376.0105 1432.4791  1453.5830 1683.9733 1860.0283  3199.9146 3544.4810 3888.8456  ***Rotational constants (GHz):***  9.7799 3.6762 2.8752  --------------------------------------------------  **TS-11A**  --------------------------------------------------  ***Vibrational frequencies (cm^-1^):***  940.0718$i$ 168.0012 271.7670  427.9933 445.0027 541.6991  648.9244 684.0267 774.5674  1056.6304 1114.1888 1198.6912  1323.3411 1337.8604 1406.1158  1493.0509 1666.3576 1847.9025  3182.1681 3585.4219 3886.7554  ***Rotational constants (GHz):***  8.2953 4.2601 3.3195  --------------------------------------------------  **TS-12A**  --------------------------------------------------  ***Vibrational frequencies (cm^-1^):***  690.5873$i$ 122.1107 277.5747  446.7249 499.1982 549.1594  581.6118 657.3799 924.7215  1048.1193 1086.6510 1189.3649  1314.0534 1373.0403 1386.1241  1600.4061 1662.5349 1951.0029  3135.0485 3600.5229 3726.8052  ***Rotational constants (GHz):***  9.6748 4.0317 3.1625 |
| --- | --- | --- |

**Table S10**. Equilibrium Constant (*K*_e_) and unimolecular (*k*_uni_) rate constants for AM0 + $\dot{O}$H radical reaction.

| 1000/T | AM0-CH | | AM0-NH_a_ | | AM0-NH_b_ | | AM0-OH | |
| --- | --- | --- | --- | --- | --- | --- | --- | --- |
|  | K_e_ | k_uni_ | K_e_ | k_uni_ | K_e_ | k_uni_ | k_e_ | k_uni_ |
| 5.00 | 1.76E-19 | 9.72E+05 | 1.76E-19 | 1.00E+06 | 1.76E-19 | 2.51E+07 | 1.76E-19 | 1.25E+06 |
| 4.76 | 9.26E-20 | 1.78E+06 | 9.26E-20 | 1.76E+06 | 9.26E-20 | 3.69E+07 | 9.26E-20 | 1.86E+06 |
| 4.55 | 5.17E-20 | 3.16E+06 | 5.17E-20 | 2.96E+06 | 5.17E-20 | 5.29E+07 | 5.17E-20 | 2.76E+06 |
| 4.35 | 3.04E-20 | 5.38E+06 | 3.04E-20 | 4.78E+06 | 3.04E-20 | 7.40E+07 | 3.04E-20 | 4.04E+06 |
| 4.17 | 1.88E-20 | 8.87E+06 | 1.88E-20 | 7.45E+06 | 1.88E-20 | 1.01E+08 | 1.88E-20 | 5.85E+06 |
| 4.00 | 1.20E-20 | 1.42E+07 | 1.20E-20 | 1.12E+07 | 1.20E-20 | 1.36E+08 | 1.20E-20 | 8.36E+06 |
| 3.85 | 8.02E-21 | 2.20E+07 | 8.02E-21 | 1.65E+07 | 8.02E-21 | 1.80E+08 | 8.02E-21 | 1.18E+07 |
| 3.70 | 5.52E-21 | 3.32E+07 | 5.52E-21 | 2.35E+07 | 5.52E-21 | 2.33E+08 | 5.52E-21 | 1.63E+07 |
| 3.57 | 3.90E-21 | 4.89E+07 | 3.90E-21 | 3.27E+07 | 3.90E-21 | 2.97E+08 | 3.90E-21 | 2.24E+07 |
| 3.45 | 2.83E-21 | 7.03E+07 | 2.83E-21 | 4.47E+07 | 2.83E-21 | 3.74E+08 | 2.83E-21 | 3.02E+07 |
| 3.33 | 2.11E-21 | 9.92E+07 | 2.11E-21 | 5.97E+07 | 2.11E-21 | 4.64E+08 | 2.11E-21 | 4.02E+07 |
| 3.23 | 1.60E-21 | 1.37E+08 | 1.60E-21 | 7.85E+07 | 1.60E-21 | 5.69E+08 | 1.60E-21 | 5.29E+07 |
| 3.13 | 1.24E-21 | 1.86E+08 | 1.24E-21 | 1.02E+08 | 1.24E-21 | 6.89E+08 | 1.24E-21 | 6.87E+07 |
| 2.50 | 2.63E-22 | 1.31E+09 | 2.63E-22 | 5.06E+08 | 2.63E-22 | 2.34E+09 | 2.63E-22 | 3.79E+08 |
| 2.22 | 1.37E-22 | 3.20E+09 | 1.37E-22 | 1.04E+09 | 1.37E-22 | 4.09E+09 | 1.37E-22 | 8.47E+08 |
| 2.00 | 8.34E-23 | 6.64E+09 | 8.34E-23 | 1.86E+09 | 8.34E-23 | 6.44E+09 | 8.34E-23 | 1.64E+09 |
| 1.82 | 5.66E-23 | 1.22E+10 | 5.66E-23 | 2.99E+09 | 5.66E-23 | 9.39E+09 | 5.66E-23 | 2.85E+09 |
| 1.67 | 4.17E-23 | 2.03E+10 | 4.17E-23 | 4.45E+09 | 4.17E-23 | 1.29E+10 | 4.17E-23 | 4.55E+09 |
| 1.54 | 3.27E-23 | 3.15E+10 | 3.27E-23 | 6.24E+09 | 3.27E-23 | 1.70E+10 | 3.27E-23 | 6.80E+09 |
| 1.43 | 2.68E-23 | 4.62E+10 | 2.68E-23 | 8.34E+09 | 2.68E-23 | 2.15E+10 | 2.68E-23 | 9.64E+09 |
| 1.33 | 2.29E-23 | 6.45E+10 | 2.29E-23 | 1.07E+10 | 2.29E-23 | 2.64E+10 | 2.29E-23 | 1.31E+10 |
| 1.25 | 2.01E-23 | 8.68E+10 | 2.01E-23 | 1.34E+10 | 2.01E-23 | 3.18E+10 | 2.01E-23 | 1.72E+10 |
| 1.18 | 1.81E-23 | 1.13E+11 | 1.81E-23 | 1.64E+10 | 1.81E-23 | 3.75E+10 | 1.81E-23 | 2.19E+10 |
| 1.11 | 1.66E-23 | 1.43E+11 | 1.66E-23 | 1.95E+10 | 1.66E-23 | 4.35E+10 | 1.66E-23 | 2.72E+10 |
| 1.05 | 1.55E-23 | 1.77E+11 | 1.55E-23 | 2.28E+10 | 1.55E-23 | 4.98E+10 | 1.55E-23 | 3.32E+10 |
| 1.00 | 1.46E-23 | 2.16E+11 | 1.46E-23 | 2.64E+10 | 1.46E-23 | 5.62E+10 | 1.46E-23 | 3.97E+10 |

**Table S11**. Equilibrium Constant (*K*_e_) and unimolecular (*k*_uni_) rate constants for AM1 + $\dot{O}$H radical reaction.

| 1000/T | AM1-CH_a_ | | AM1-CH_b_ | | AM1-CH_c_ | | AM1-NH | | AM1-OH | |
| --- | --- | --- | --- | --- | --- | --- | --- | --- | --- | --- |
|  | K_e_ | k_uni_ | K_e_ | k_uni_ | K_e_ | k_uni_ | K_e_ | k_uni_ | K_e_ | k_uni_ |
| 5.00 | 2.08E-20 | 2.86E+07 | 7.94E-19 | 4.22E+05 | 7.94E-19 | 3.37E+06 | 2.08E-20 | 1.25E+09 | 2.08E-20 | 2.19E+06 |
| 4.76 | 1.17E-20 | 4.54E+07 | 3.94E-19 | 8.52E+05 | 3.94E-19 | 5.78E+06 | 1.17E-20 | 1.51E+09 | 1.17E-20 | 3.25E+06 |
| 4.55 | 6.95E-21 | 7.01E+07 | 2.08E-19 | 1.62E+06 | 2.08E-19 | 9.53E+06 | 6.95E-21 | 1.82E+09 | 6.95E-21 | 4.77E+06 |
| 4.35 | 4.33E-21 | 1.05E+08 | 1.17E-19 | 2.93E+06 | 1.17E-19 | 1.52E+07 | 4.33E-21 | 2.17E+09 | 4.33E-21 | 6.96E+06 |
| 4.17 | 2.81E-21 | 1.54E+08 | 6.86E-20 | 5.07E+06 | 6.86E-20 | 2.33E+07 | 2.81E-21 | 2.57E+09 | 2.81E-21 | 1.00E+07 |
| 4.00 | 1.89E-21 | 2.21E+08 | 4.22E-20 | 8.41E+06 | 4.22E-20 | 3.49E+07 | 1.89E-21 | 3.02E+09 | 1.89E-21 | 1.43E+07 |
| 3.85 | 1.32E-21 | 3.09E+08 | 2.70E-20 | 1.35E+07 | 2.70E-20 | 5.08E+07 | 1.32E-21 | 3.53E+09 | 1.32E-21 | 2.02E+07 |
| 3.70 | 9.41E-22 | 4.23E+08 | 1.79E-20 | 2.09E+07 | 1.79E-20 | 7.22E+07 | 9.41E-22 | 4.09E+09 | 9.41E-22 | 2.82E+07 |
| 3.57 | 6.91E-22 | 5.70E+08 | 1.22E-20 | 3.14E+07 | 1.22E-20 | 1.00E+08 | 6.91E-22 | 4.72E+09 | 6.91E-22 | 3.88E+07 |
| 3.45 | 5.19E-22 | 7.54E+08 | 8.58E-21 | 4.60E+07 | 8.58E-21 | 1.37E+08 | 5.19E-22 | 5.40E+09 | 5.19E-22 | 5.27E+07 |
| 3.33 | 3.98E-22 | 9.82E+08 | 6.18E-21 | 6.59E+07 | 6.18E-21 | 1.83E+08 | 3.98E-22 | 6.15E+09 | 3.98E-22 | 7.07E+07 |
| 3.23 | 3.11E-22 | 1.26E+09 | 4.55E-21 | 9.24E+07 | 4.55E-21 | 2.40E+08 | 3.11E-22 | 6.96E+09 | 3.11E-22 | 9.36E+07 |
| 3.13 | 2.47E-22 | 1.60E+09 | 3.42E-21 | 1.27E+08 | 3.42E-21 | 3.11E+08 | 2.47E-22 | 7.84E+09 | 2.47E-22 | 1.23E+08 |
| 2.50 | 6.21E-23 | 7.22E+09 | 6.05E-22 | 9.43E+08 | 6.05E-22 | 1.60E+09 | 6.21E-23 | 1.72E+10 | 6.21E-23 | 7.26E+08 |
| 2.22 | 3.48E-23 | 1.45E+10 | 2.88E-22 | 2.35E+09 | 2.88E-22 | 3.40E+09 | 3.48E-23 | 2.51E+10 | 3.48E-23 | 1.69E+09 |
| 2.00 | 2.24E-23 | 2.55E+10 | 1.63E-22 | 4.93E+09 | 1.63E-22 | 6.26E+09 | 2.24E-23 | 3.45E+10 | 2.24E-23 | 3.40E+09 |
| 1.82 | 1.59E-23 | 4.09E+10 | 1.04E-22 | 9.13E+09 | 1.04E-22 | 1.04E+10 | 1.59E-23 | 4.51E+10 | 1.59E-23 | 6.11E+09 |
| 1.67 | 1.21E-23 | 6.11E+10 | 7.23E-23 | 1.53E+10 | 7.23E-23 | 1.60E+10 | 1.21E-23 | 5.68E+10 | 1.21E-23 | 1.01E+10 |
| 1.54 | 9.78E-24 | 8.61E+10 | 5.40E-23 | 2.39E+10 | 5.40E-23 | 2.32E+10 | 9.78E-24 | 6.95E+10 | 9.78E-24 | 1.54E+10 |
| 1.43 | 8.24E-24 | 1.16E+11 | 4.25E-23 | 3.52E+10 | 4.25E-23 | 3.20E+10 | 8.24E-24 | 8.29E+10 | 8.24E-24 | 2.24E+10 |
| 1.33 | 7.18E-24 | 1.51E+11 | 3.50E-23 | 4.92E+10 | 3.50E-23 | 4.24E+10 | 7.18E-24 | 9.71E+10 | 7.18E-24 | 3.11E+10 |
| 1.25 | 6.43E-24 | 1.90E+11 | 2.97E-23 | 6.63E+10 | 2.97E-23 | 5.45E+10 | 6.43E-24 | 1.12E+11 | 6.43E-24 | 4.16E+10 |
| 1.18 | 5.88E-24 | 2.34E+11 | 2.60E-23 | 8.65E+10 | 2.60E-23 | 6.81E+10 | 5.88E-24 | 1.27E+11 | 5.88E-24 | 5.39E+10 |
| 1.11 | 5.48E-24 | 2.82E+11 | 2.32E-23 | 1.10E+11 | 2.32E-23 | 8.31E+10 | 5.48E-24 | 1.43E+11 | 5.48E-24 | 6.81E+10 |
| 1.05 | 5.17E-24 | 3.34E+11 | 2.11E-23 | 1.36E+11 | 2.11E-23 | 9.96E+10 | 5.17E-24 | 1.59E+11 | 5.17E-24 | 8.41E+10 |
| 1.00 | 4.95E-24 | 3.90E+11 | 1.95E-23 | 1.65E+11 | 1.95E-23 | 1.17E+11 | 4.95E-24 | 1.75E+11 | 4.95E-24 | 1.02E+11 |

**Table S12**. Equilibrium Constant (*K*_e_) and unimolecular (*k*_uni_) rate constants for AM2 + $\dot{O}$H radical reaction.

| 1000/T | AM2-CH | | AM2-NH | | AM2-OH_a_ | | AM2-OH_a_ | |
| --- | --- | --- | --- | --- | --- | --- | --- | --- |
|  | K_e_ | k_uni_ | K_e_ | k_uni_ | K_e_ | k_uni_ | K_e_ | k_uni_ |
| 5.00 | 1.53E-19 | 5.03E+08 | 1.53E-19 | 7.15E+06 | 1.53E-19 | 5.83E+07 | 1.53E-19 | 1.68E+05 |
| 4.76 | 7.69E-20 | 7.46E+08 | 7.69E-20 | 1.17E+07 | 7.69E-20 | 7.07E+07 | 7.69E-20 | 2.49E+05 |
| 4.55 | 4.13E-20 | 1.08E+09 | 4.13E-20 | 1.85E+07 | 4.13E-20 | 8.56E+07 | 4.13E-20 | 3.71E+05 |
| 4.35 | 2.34E-20 | 1.52E+09 | 2.34E-20 | 2.85E+07 | 2.34E-20 | 1.04E+08 | 2.34E-20 | 5.52E+05 |
| 4.17 | 1.40E-20 | 2.09E+09 | 1.40E-20 | 4.26E+07 | 1.40E-20 | 1.25E+08 | 1.40E-20 | 8.19E+05 |
| 4.00 | 8.69E-21 | 2.82E+09 | 8.69E-21 | 6.22E+07 | 8.69E-21 | 1.51E+08 | 8.69E-21 | 1.21E+06 |
| 3.85 | 5.62E-21 | 3.73E+09 | 5.62E-21 | 8.85E+07 | 5.62E-21 | 1.82E+08 | 5.62E-21 | 1.78E+06 |
| 3.70 | 3.76E-21 | 4.85E+09 | 3.76E-21 | 1.23E+08 | 3.76E-21 | 2.18E+08 | 3.76E-21 | 2.60E+06 |
| 3.57 | 2.60E-21 | 6.20E+09 | 2.60E-21 | 1.69E+08 | 2.60E-21 | 2.60E+08 | 2.60E-21 | 3.75E+06 |
| 3.45 | 1.84E-21 | 7.83E+09 | 1.84E-21 | 2.26E+08 | 1.84E-21 | 3.10E+08 | 1.84E-21 | 5.37E+06 |
| 3.33 | 1.34E-21 | 9.75E+09 | 1.34E-21 | 2.98E+08 | 1.34E-21 | 3.68E+08 | 1.34E-21 | 7.61E+06 |
| 3.23 | 9.94E-22 | 1.20E+10 | 9.94E-22 | 3.86E+08 | 9.94E-22 | 4.35E+08 | 9.94E-22 | 1.07E+07 |
| 3.13 | 7.54E-22 | 1.46E+10 | 7.54E-22 | 4.94E+08 | 7.54E-22 | 5.13E+08 | 7.54E-22 | 1.48E+07 |
| 2.50 | 1.42E-22 | 5.08E+10 | 1.42E-22 | 2.35E+09 | 1.42E-22 | 1.62E+09 | 1.42E-22 | 1.35E+08 |
| 2.22 | 6.98E-23 | 9.02E+10 | 6.98E-23 | 4.81E+09 | 6.98E-23 | 2.93E+09 | 6.98E-23 | 3.97E+08 |
| 2.00 | 4.05E-23 | 1.44E+11 | 4.05E-23 | 8.58E+09 | 4.05E-23 | 4.87E+09 | 4.05E-23 | 9.76E+08 |
| 1.82 | 2.64E-23 | 2.13E+11 | 2.64E-23 | 1.39E+10 | 2.64E-23 | 7.55E+09 | 2.64E-23 | 2.08E+09 |
| 1.67 | 1.88E-23 | 2.97E+11 | 1.88E-23 | 2.08E+10 | 1.88E-23 | 1.11E+10 | 1.88E-23 | 3.97E+09 |
| 1.54 | 1.43E-23 | 3.95E+11 | 1.43E-23 | 2.94E+10 | 1.43E-23 | 1.55E+10 | 1.43E-23 | 6.94E+09 |
| 1.43 | 1.15E-23 | 5.05E+11 | 1.15E-23 | 3.97E+10 | 1.15E-23 | 2.09E+10 | 1.15E-23 | 1.13E+10 |
| 1.33 | 9.59E-24 | 6.28E+11 | 9.59E-24 | 5.17E+10 | 9.59E-24 | 2.72E+10 | 9.59E-24 | 1.73E+10 |
| 1.25 | 8.26E-24 | 7.61E+11 | 8.26E-24 | 6.52E+10 | 8.26E-24 | 3.45E+10 | 8.26E-24 | 2.53E+10 |
| 1.18 | 7.31E-24 | 9.04E+11 | 7.31E-24 | 8.02E+10 | 7.31E-24 | 4.28E+10 | 7.31E-24 | 3.56E+10 |
| 1.11 | 6.61E-24 | 1.06E+12 | 6.61E-24 | 9.66E+10 | 6.61E-24 | 5.20E+10 | 6.61E-24 | 4.83E+10 |
| 1.05 | 6.08E-24 | 1.21E+12 | 6.08E-24 | 1.14E+11 | 6.08E-24 | 6.22E+10 | 6.08E-24 | 6.36E+10 |
| 1.00 | 5.67E-24 | 1.38E+12 | 5.67E-24 | 1.33E+11 | 5.67E-24 | 7.33E+10 | 5.67E-24 | 8.17E+10 |

**References**

Becke, A. D. (1988). Density-functional exchange-energy approximation with correct asymptotic behavior. *Phys Rev A  (Coll Park)* 38, 3098–3100. doi: 10.1103/PhysRevA.38.3098

Goedecker, S., Teter, M., and Hutter, J. (1996). Separable dual-space Gaussian pseudopotentials. *Phys Rev B* 54, 1703–1710. doi: 10.1103/PhysRevB.54.1703

Grimme, S., Antony, J., Ehrlich, S., and Krieg, H. (2010). A consistent and accurate *ab initio* parametrization of density functional dispersion correction (DFT-D) for the 94 elements H-Pu. *J Chem Phys* 132, 154104. doi: 10.1063/1.3382344

Hartwigsen, C., Goedecker, S., and Hutter, J. (1998). Relativistic separable dual-space Gaussian pseudopotentials from H to Rn. *Phys Rev B* 58, 3641–3662. doi: 10.1103/PhysRevB.58.3641

Lee, C., Yang, W., and Parr, R. G. (1988). Development of the Colle-Salvetti correlation-energy formula into a functional of the electron density. *Phys Rev B* 37, 785–789. doi: 10.1103/PhysRevB.37.785

VandeVondele, J., and Hutter, J. (2007). Gaussian basis sets for accurate calculations on molecular systems in gas and condensed phases. *J Chem Phys* 127, 114105. doi: 10.1063/1.2770708

VandeVondele, J., Krack, M., Mohamed, F., Parrinello, M., Chassaing, T., and Hutter, J. (2005). Quickstep: Fast and accurate density functional calculations using a mixed Gaussian and plane waves approach. *Comput Phys Commun* 167, 103–128. doi: https://doi.org/10.1016/j.cpc.2004.12.014
